# Supplementary material for: Delegating Sex: Differential Gene Expression in Stolonizing Syllids Uncovers the Hormonal Control of Reproduction
Source: Genome Biol Evol. 2018 Dec 11;11(1):295–318. doi: 10.1093/gbe/evy265 (PMC6350857; doi:10.1093/gbe/evy265)
Supplement: Supplementary Data [file evy265_supp.zip › SuppFile_S1_Statistics_REFSOM_REFTOTREPRO.pdf]

General characterization of the* de novo *transcriptomes

The reference transcriptome REFSOM was constructed with 249,599,131 trimmed reads from 23 somatic tissue libraries (anterior part, proventricle and final segments) from all specimens regardless of their reproductive stage, that resulted in 637,115 unique transcripts with a total length of ~432 Mb (Table 1). The REFTOTREPRO reference transcriptome was based on 169,757,021 trimmed reads from 18 libraries of somatic (anterior part, proventricle and final segments) and reproductive (anterior half part of stolon and posterior half part of stolon) tissues from all reproductive specimens (both males and females), that generated 77,831 unique transcripts with a total length of ~242 Mb (Table 2). The spliced isoforms included in the assemblies were ~43% of the total assembly both in REFSOM and REFTOTREPRO. The N50 was over 700 nt in REFSOM and over 1000 nt in REFTOTREPRO; the GC content was ~40% in both assemblies; the median and average transcript lengths in REFSOM were 454 and 677 nt, respectively, and 374 and 679 nt in REFTOTREPRO (Tables 1 and 2). These numbers are above the average N50 values and average transcript lengths obtained from other marine annelids (e.g., Riesgo et al. 2012; Andrade et al. 2015), although smaller than the N50 values obtained for oligochaetes (Novo et al. 2016). The results obtained from BLASTX against a metazoan selection of proteins from the *nr* database, identified 157,035 transcripts with blast hits in REFSOM and 88,009 transcripts in REFTOTREPRO, which represent ~25% of the reference transcriptomes. In addition, we obtained annotations (Go terms) for 156,594 transcripts in REFSOM and 82,273 in REFTOTREPRO, which represent ~23–25% of the reference transcriptomes (see also Supplementary File S2).

The number of aligned reads from the different tissues and specimens mapped onto the *de novo* assemblies varied from ~5,000,000 to ~19,000,000 for each reference transcriptome (Tables 1 and 2). However, the reads that yielded transcripts varied from ~27,000 to ~200,000 in REFSOM (Table 1), and from ~37,000 to ~100,000 in REFTOTREPRO (Table 2).

		Total Reads	Aligned Reads	% Aligned Reads	Transcripts	Genes	% Genes	N50	Hit_Mtz	Annot	% GC	MTL	ATL	Mb
Biological Replicates	Reference transcriptome **REFSOM**	249,599,131			637,115	359,341	56.4	756	157,035	156,594	40	454	677.51	431.6
Indiv. 1	SMAG0A	44,200,803	9,650,828	21.8	111,389	76,369	68.5							
Indiv. 2	SMAG1A	57,662,501	21,362,825	37.0	124,841	95,316	73.3							
Indiv. 3	SMAG2A	44,110,854	13,454,458	30.5	27,201	20,506	75.3							
Indiv. 4	SMAG3A	35,289,501	14,775,067	41.8	128,885	94,450	73.3							
Indiv. 5	SMAG4A	34,342,939	12,194,414	35.5	108,173	77,478	71.6							
Indiv. 6	SMAG5A	20,029,097	8,692,094	43.4	142,810	100,008	70.0							
Indiv. 7	SMAG6A	28,218,714	11,697,704	41.4	154,146	108,440	70.3							
Indiv. 8	SMAG7A	17,318,639	6,332,572	36.5	92,224	87,925	95.3							
Indiv. 2	SMAG1P	15,706,492	5,937,102	37.8	89,186	62,324	69.8							
Indiv. 3	SMAG2P	18,039,837	5,056,249	28.0	50,903	34,986	68.7							
Indiv. 4	SMAG3P	43,183,317	16,805,848	38.9	74,304	53,450	71.9							
Indiv. 5	SMAG4P	33,938,109	10,845,806	31.9	97,462	67,516	69.3							
Indiv. 6	SMAG5P	36,005,489	14,264,683	39.6	106,434	75,320	70.7							
Indiv. 7	SMAG6P	29,763,664	11,640,934	39.1	101,180	72,092	71.2							
Indiv. 8	SMAG7P	39,285,447	13,926,770	35.4	187,951	139,170	74.0							
Indiv. 1	SMAG0F	30,946,192	9,787,032	31.6	118,307	81,240	68.6							
Indiv. 2	SMAG1F	20,619,876	6,691,141	32.4	84,421	74,776	88.5							
Indiv. 3	SMAG2F	11,861,695	5,582,524	47.0	17,011	15,208	89.4							
Indiv. 4	SMAG3F	39,686,245	15,165,310	38.2	149,680	107,367	71.7							
Indiv. 5	SMAG4F	21,809,808	6,121,405	28.0	104,452	71,178	68.1							
Indiv. 6	SMAG5F	42,233,329	13,956,719	33.0	165,297	117,388	71.0							
Indiv. 7	SMAG6F	28,740,151	6,891,558	23.9	135,798	94,162	69.3							
Indiv. 8	SMAG7F	25,478,901	8,763,875	34.4	208,055	156,890	75.4							

Table 1. General statistics of the ‘reference’ de novo transcriptome (REFSOM) for the somatic parts of NON-REPRO and REPRO individuals of *S. Magdalena* (SMAG), and for the 3 different tissues mapped against REFSOM (Anterior part, Proventricle and Final part).

		Total Reads	Aligned Reads	% Aligned Reads	Align. Transcr.	Genes	% Genes	N50	Hit_Mtz	Annot	% GC	MTL	ATL	Mb
Biological Replicates	Reference transcriptome **REFTOTREPRO**	169,757,021			356,464	203,068	56.9	1082	88,009	82,273	40.5	374	679.51	242.2
Indiv. 1	SMAG0A	44,200,803	9,948,328	22.5	77,831	52,098	66.9							
Indiv. 2	SMAG1A	57,662,501	18,806,237	32.6	97,882	60,496	61.8							
Indiv. 3	SMAG2A	44,110,854	14,644,348	33.2	20,022	15,443	77.1							
Indiv. 4	SMAG3A	35,289,501	12,775,973	36.2	99,961	63,991	64.0							
Indiv. 2	SMAG1P	15,706,492	5,303,443	33.7	64,101	42,762	66.7							
Indiv. 3	SMAG2P	18,039,837	6,578,256	36.4	37,061	25,107	67.7							
Indiv. 4	SMAG3P	43,183,317	14,590,640	33.7	84,073	53,450	63.5							
Indiv. 1	SMAG0F	30,946,192	10,556,603	34.1	87,273	54,183	62,1							
Indiv. 2	SMAG1F	20,619,876	5,779,527	28.0	73,683	42,762	58.0							
Indiv. 3	SMAG2F	11,861,695	5,445,306	45.9	17,349	12,645	72.8							
Indiv. 4	SMAG3F	39,686,245	12,899,516	32.5	99,193	71,156	63.4							
Indiv. 1	SMAG0AS	31,539,262	8,596,837	27.2	73,612	51,251	69.6							
Indiv. 3	SMAG2AS	36,797,621	10,062,912	27.3	67,010	44,587	66.5							
Indiv. 4	SMAG3AS	17,125,847	4,363,795	25.4	85,575	57,064	66.6							
Indiv. 1	SMAG0FS	24,933,766	6,259,604	25.1	82,481	53,119	64.4							
Indiv. 2	SMAG1FS	42,141,839	9,258,201	21.9	81,765	52,699	64.4							
Indiv. 3	SMAG2FS	20,856,398	6,512,586	31.2	62,007	39,357	63.5							
Indiv. 4	SMAG3FS	29,231,512	7,374,909	25.2	94,353	63,294	67.1							

Table 2. General statistics of the ‘reference’ de novo transcriptome (REFTOTOREPRO) for the reproductive specimens of *S. magdalena*, and for the 5 different tissues mapped against REFTOTREPRO (Anterior part, Proventricle, Final part, Anterior and Posterior half parts of Stolons).
