# Supplementary material for: Delegating Sex: Differential Gene Expression in Stolonizing Syllids Uncovers the Hormonal Control of Reproduction
Source: Genome Biol Evol. 2018 Dec 11;11(1):295–318. doi: 10.1093/gbe/evy265 (PMC6350857; doi:10.1093/gbe/evy265)
Supplement: Supplementary Data [file evy265_supp.zip › SuppFile_S6_REFSOM.pdf]

			Contig ID	Contig Length	log2FoldChange	padj	evalue2	Description	GO IDs
FEMALE REPRODUCTIVE vs NON REPRODUCTIVE	A	Upregulated in Female	157874_c0_g1	386	479.337.238.801.304	0.00561333585160386	4.0E-6	BF-DED-NACHT	P:GO:0042981
			167328_c0_g1	419	507.452.779.567.669	0.00182197593361259	6,00E-16	zinc metalloproteinase nas-13-like	F:GO:0008233
			190569_c1_g12	340	489.401.950.990.912	0.0038590982759183	0.58	probable chitinase	F:GO:0008061; P:GO:0006030; C:GO:0005576
			210174_c2_g3	629	54.861.648.130.281	3,81E+09	1,00E-16	trefoil factor 2-like	0
			211853_c7_g10	429	462.385.410.962.427	0.00814422732283239	9.1E-7	Complement receptor type 2	0
			211853_c7_g25	418	482.085.895.482.304	0.00413918082401764	3.6E-23	tyrosine-protein kinase Src42A	P:GO:0018108; F:GO:0005524; F:GO:0004713; C:GO:0016020; C:GO:0016021; F:GO:0004672; P:GO:0016310; P:GO:0006468; F:GO:0016301
		Upregulated in Non Reproductive	203518_c0_g1	1048	-454.069.939.450.147	0.0038590982759183	4.4E-17	Ribonuclease H1	0
			205772_c0_g24	308	-583.018.537.254.658	9,65E+07	5,00E-44	T-complex protein 1 subunit epsilon-like	F:GO:0005524; C:GO:0005737; F:GO:0051082; P:GO:0006457
	P	Upregulated in Female	180071_c0_g4	815	596.295.284.445.103	0.00998352582812103	5.2E-12	cubilin	F:GO:0005509; P:GO:0007155; F:GO:0005540
			204078_c1_g1	898	515.515.097.616.284	0.00440244452124048	3.8E-18	serine protease 27	0
			212550_c6_g3	1868	419.043.727.347.284	0.00367978955766449	3.0E-23	MAM and LDL-receptor class A domain-containing	0
		Upregulated in Non Reproductive	186330_c2_g1	535	-700.909.445.742.978	0.00607101048508667	3.00E-90	Myosin heavy chain	C:GO:0016459; F:GO:0005524; F:GO:0003779; P:GO:0008152; F:GO:0016597; F:GO:0003774
			186732_c5_g4	563	-568.592.068.141.675	0.0081423910048116	3.4E-79	glutamine synthetase	F:GO:0005524; P:GO:0006542; F:GO:0004356
			189243_c0_g6	357	-728.501.800.310.916	0.00695654534715852	4.4E-79	actin isoform zwei	0
			203272_c0_g2	1489	-900.545.820.071.575	0.00695654534715852	2.0E-103	chaperonin containing TCP1 subunit 5 S	0
			209862_c1_g3	424	-445.356.640.702.451	0.00695654534715852	8.5E-41	T-box 2-like isoform X2	C:GO:0005634; F:GO:0003677; F:GO:0003700; P:GO:0006351; P:GO:0006355
			211073_c1_g2	325	-720.845.790.874.088	0.00695654534715852	1.3E-10	PREDICTED: obscurin-like	0
	F	Upregulated in Female	174615_c0_g1	553	328.789.520.363.522	0.00459383201324909	1.3E-52	Microsomal glutathione S-transferase partial	C:GO:0005739; C:GO:0016020; P:GO:0008152; P:GO:0042221; F:GO:0016740; P:GO:0009987
			176942_c0_g1	1194	650.621.201.236.462	0.000246844476300749	9.6E-12	Multiple epidermal growth factor-like domains 10	0
			178073_c0_g2	1361	527.747.295.234.848	0.00459383201324909	9,00E-13	zinc finger MYM-type 2-like	F:GO:0003677; P:GO:0006811; P:GO:0034220; C:GO:0016020; C:GO:0016021; P:GO:0015074; P:GO:0006310; F:GO:0005230; P:GO:0006810
			178607_c0_g6	603	433.425.323.750.416	0.00892754783636636	2,00E-05	protocadherin beta-12	C:GO:0016020
			179140_c0_g1	1139	694.411.209.315.545	8,82E+09	1,00E-22	collagen alpha-1(XII)æ	F:GO:0005509
			181366_c0_g1	2362	854.025.321.754.027	1,91E+06	1,00E-22	collagen alpha-1(XII)æ	0
			181381_c0_g1	1484	7.150.721.759.767	8,16E+09	9.6E-12	transcription factor HES-1 isoform X1	F:GO:0003677; C:GO:0005634; P:GO:0006351; P:GO:0006355; F:GO:0046983
			181461_c1_g1	549	679.118.065.655.128	0.000204843682103449	1.5E-34	15-hydroxyprostaglandin dehydrogenase [NAD+]	F:GO:0016491; P:GO:0055114
			181496_c1_g3	579	656.804.255.944.089	0.000672172189569433	9.9E-20	15-hydroxyprostaglandin dehydrogenase	F:GO:0016491; P:GO:0055114
			181584_c1_g4	1478	323.034.603.691.277	0.00263754586560203	7,00E-33	proton-coupled folate transporter-like	P:GO:0055085; C:GO:0016020; C:GO:0016021
			185921_c0_g2	1500	691.563.872.002.897	0.000340202958054035	5.2E-13	tripartite motif-containing 16-like	0
			187269_c1_g3	3512	544.645.370.880.233	6,22E+09	2,00E-12	tripartite motif-containing protein 52	F:GO:0008270; F:GO:0046872; C:GO:0016020; C:GO:0016021; C:GO:0005622
			187817_c0_g7	809	889.495.766.148.914	4,63E-01	5.0E-11	Sortilin-related receptor	C:GO:0016020; C:GO:0016021
			190860_c0_g2	1492	892.988.363.175.569	3,74E+06	1.8E-40	ascorbate peroxidase-like	0
			191325_c0_g1	1494	7.341.184.083.981	3,60E+09	8,00E-15	collagen alpha-1(XII)æ	0
			191370_c1_g2	1453	774.842.269.526.025	2,61E+08	3,00E-13	collagen alpha-1(XII)æ	0
			191413_c1_g1	2397	495.157.605.059.411	0.00497045097834525	1,65E-35	tolloid 2 isoform X4	0
			192451_c0_g1	1602	883.558.162.441.722	6,12E+04	2,00E-46	G2/mitotic-specific cyclin-B-like	C:GO:0005634
			192543_c0_g1	3239	878.204.270.418.925	6,50E+06	1,00E-92	collagen alpha-1(XII)æ	F:GO:0005509
			193741_c0_g1	2293	998.383.678.670.932	8,00E-01	2,00E-116	collagen alpha-1(XII)æ	F:GO:0005509
			194105_c0_g1	1332	771.815.910.359.099	4,21E+08	7,00E-21	fibulin-5	F:GO:0000166; F:GO:0005524; F:GO:0005509; F:GO:0004672; P:GO:0016310; F:GO:0016740; F:GO:0004674; P:GO:0006468; F:GO:0016301
			194318_c0_g2	2825	683.135.582.263.481	0.000242187736225485	6.1E-91	Ovochymase-1	P:GO:0006508; F:GO:0004252; F:GO:0008236; F:GO:0008233; F:GO:0016787
			194722_c1_g9	815	312.945.882.312.834	0.00580459188566755	1,00E-11	collagen alpha-1(XII)æ	F:GO:0005509
			194761_c1_g1	1018	719.628.276.620.959	1,86E+09	2,61E-10	aldo keto reductase	F:GO:0016491; P:GO:0055114
			195242_c0_g1	2269	815.827.796.489.712	1,11E+07	1.0E-11	tripartite motif-containing protein 2-like	F:GO:0008270; F:GO:0046872; C:GO:0005622
			195261_c1_g1	805	749.047.863.193.782	1,53E+06	3.6E-41	fibrillin-1- partial	F:GO:0005509
			195285_c0_g1	1019	469.462.413.712.386	0.000754737570984938	1,47E-61	organic cation transporter -like isoform X2	P:GO:0055085; F:GO:0022857; C:GO:0016020; C:GO:0016021
			195919_c0_g8	1300	440.857.128.641.457	0.00200303001787336	4,03E-87	uncharacterized oxidoreductase -like	F:GO:0016491; P:GO:0008152; P:GO:0055114
			196174_c0_g1	1019	695.704.302.541.738	1,06E+08	2,50E-55	pre-B-cell leukemia transcription factor 4-like	F:GO:0003677; C:GO:0005634; P:GO:0006355
			196525_c0_g1	2141	823.296.585.497.034	3,29E+06	5.5E-38	cell division cycle 20 homolog	0
			197079_c3_g7	1610	507.716.827.063.679	0.0040887499055627	1.0E-43	Williams-Beuren syndrome chromosomal	C:GO:0005739; F:GO:0008757; P:GO:0032259
			197614_c0_g2	302	311.195.175.590.891	0.00685961498440997	2,51E-31	ribosome biogenesis TSR3 homolog	0
			197994_c1_g2	921	22.319.310.602.854	0.00670428375314351	4.0E-30	delta-like protein C isoform X1	P:GO:0007275; P:GO:0007154; F:GO:0005509; C:GO:0016020; C:GO:0016021
			198379_c0_g2	2587	761.542.526.079.145	4,58E+08	1,51E-74	sodium- and chloride-dependent glycine	0
			198862_c0_g1	2748	547.142.107.035.291	0.000697512544869332	1,08E-128	neuronal acetylcholine receptor subunit alpha-9-	0
			201115_c3_g4	624	458.615.017.055.015	0.000688869791273366	1,21E-33	NHP2 1 homolog	F:GO:0003723; C:GO:0030529; P:GO:0042254; C:GO:0005730
			201302_c0_g1	675	509.410.333.139.113	0.00071018917876565	1,13E-22	Histone-binding N1 N2	0
			201334_c0_g3	1782	477.473.660.401.469	0.00766050746659344	1,70E-26	transcription factor Sox-2 isoform X4	0
			201713_c1_g5	3907	612.726.477.815.643	4,90E+09	1,10E-122	vitellogenin	P:GO:0006869; F:GO:0005319
			201980_c1_g1	1154	910.646.508.992.767	9,03E-01	5,85E-23	collagen alpha-1(XII) chain-like	F:GO:0005509
			202159_c0_g3	3941	583.823.739.001.727	1,14E+09	2,15E-31	vitellogenin	P:GO:0006869; F:GO:0005319
			202621_c0_g3	2296	546.144.949.678.181	0.00357781069573743	1,16E-62	solute carrier organic anion transporter family	P:GO:0006811; C:GO:0016020; C:GO:0016021; P:GO:0006810; C:GO:0005886; F:GO:0005215
			202881_c2_g2	1767	718.867.986.576.135	0.000132101062627503	6.0E-54	collagen alpha-1(XII) chain-like	0
			202881_c2_g3	561	685.742.268.654.043	0.000353356426452959	3,71E-26	Ribosome biogenesis regulatory	F:GO:0005509
			203057_c0_g3	2833	61.502.881.255.637	3,08E+09	1.2E-129	cyclin A	P:GO:0044763
			203062_c1_g17	919	386.736.308.664.673	0.0047391686976734	1.0E-131	phosphoethanolamine N-methyltransferase-like	F:GO:0008168; P:GO:0032259
			203292_c2_g1	2102	95.342.344.655.273	1,00E+03	9.0E-18	tripartite motif-containing protein 2-like	F:GO:0008270; F:GO:0046872; C:GO:0005622
			203318_c0_g2	4142	977.985.912.367.864	3,07E+02	1.4E-56	leucine-rich repeat-containing 1	F:GO:0005524; P:GO:0018108; F:GO:0004713; C:GO:0016020; C:GO:0016021; F:GO:0004672; P:GO:0016310; P:GO:0006468; F:GO:0016301
			203318_c0_g3	2822	991.227.118.153.637	6,09E+01	9.6E-35	insulin-like growth factor-binding complex acid labile	P:GO:0018108; F:GO:0005524; F:GO:0004713; C:GO:0016020; C:GO:0016021; F:GO:0004672; P:GO:0016310; P:GO:0006468; F:GO:0016301
			204442_c1_g3	2376	627.542.998.699.775	0.000183722049739255	9,06E-08	vitelline envelope zona pellucida domain 5	C:GO:0016020; C:GO:0016021
			204448_c0_g1	1008	678.302.280.155.002	0.000384811969152122	1,15E-04	vitelline envelope zona pellucida domain 16	C:GO:0016020; C:GO:0016021
			206303_c0_g3	3985	371.001.570.043.456	0.00616757078406841	1,65E-06	zinc finger 501-like isoform X2	0
			206479_c0_g8	1323	386.913.743.275.329	8,67E+08	8,79E-148	ammonium transporter Rh type A-like	P:GO:0015696; C:GO:0016020
			206490_c0_g13	310	664.927.671.198.243	0.000190617374627958	4,82E-13	regulatory P domain-containing	C:GO:0016020; C:GO:0016021
			207067_c0_g1	3981	632.432.649.939.147	6,05E+08	2,21E-31	transcriptional regulatory ASH1-like isoform X1	F:GO:0005488
			207249_c2_g8	1315	553.379.520.555.804	8,67E+08	2.0E-45	insulin-like growth factor-binding complex acid labile	0
			207595_c0_g1	856	636.508.773.720.567	5,28E+06	4.2E-15	VKT2_ANESU ame: Full=Kunitz-type protease	F:GO:0030414; F:GO:0004867; C:GO:0042151; P:GO:0010951; C:GO:0005576; P:GO:0010466
			207828_c3_g1	1082	971.613.472.849.982	5,72E+01	4.0E-118	cytoplasmic polyadenylation element-	F:GO:0000166; F:GO:0003676
			208153_c0_g4	2729	171.402.582.637.717	0.00929018985738107	2,38E-26	zinc finger CCCH domain-containing 13-like isoform	F:GO:0046872
			208573_c1_g10	508	560.486.409.148.265	0.00685961498440997	1.1E-13	C1q domain containing 1q37	0
			208573_c1_g10	524	560.486.409.148.265	0.00685961498440997	1.7E-11	C1q domain containing 1q37	0
			208759_c0_g3	386	565.173.584.513.716	0.00799725050194009	2,21E-09	collagen alpha-3(VI) chain	F:GO:0005509; C:GO:0005581; C:GO:0016020; C:GO:0016021; C:GO:0005578
			208759_c0_g4	1947	746.738.656.501.033	3,28E+08	3,94E-18	collagen alpha-3(VI) chain-like isoform X1	F:GO:0005509; C:GO:0005581
			208759_c0_g5	3424	760.671.185.563.687	1,13E+08	1,23E-97	collagen alpha-1(I) chain-like	C:GO:0005615; C:GO:0005581; F:GO:0005509; C:GO:0016020; C:GO:0016021; C:GO:0005576; C:GO:0005578
			208759_c0_g6	1257	756.474.349.030.558	3,28E+08	2.0E-31	Cochlin	F:GO:0005509
			209493_c1_g1	1387	318.304.435.125.481	3,33E+07	2,69E-31	von Willebrand factor D and EGF domain-	0
			209584_c1_g12	321	252.010.627.469.991	0.00685961498440997	5.4E-48	signal recognition particle subunit SRP68-like	0
			209838_c1_g5	1479	264.056.535.106.804	0.00370764796519084	2,99E-68	RNA-binding 26-like	0
			210351_c1_g7	2885	271.092.566.029.781	0.00943435245439491	8.0E-176	acid trehalase-like protein 1	P:GO:0005975; F:GO:0003824; C:GO:0005618; F:GO:0016787
			211129_c1_g11	2732	337.440.876.184.768	0.00942914157603796	7,14E-57	H ACA ribonucleo complex non-core subunit NAF1	P:GO:0001522; P:GO:0042254
			211371_c4_g13	1792	352.346.348.329.373	0.00810469973468925	2.6E-71	kelch 18	0
			211829_c1_g2	1174	896.184.847.557.543	6,04E+02	4.7E-6	cytoplasmic polyadenylation element-	0

		211853_c7_g24	418	680.031.127.832.529	1,10E+08	6.0E-22	relaxin receptor 2	P:GO:0018108; F:GO:0005524; F:GO:0004713; C:GO:0016020; C:GO:0016021; F:GO:0004672; P:GO:0016310; P:GO:0006468; F:GO:0016301
		211853_c7_g25	418	730.593.705.885.329	1,41E+09	2.6E-22	tyrosine-protein kinase Src42A	P:GO:0018108; F:GO:0005524; F:GO:0004713; C:GO:0016020; C:GO:0016021; F:GO:0004672; P:GO:0016310; P:GO:0006468; F:GO:0016301
		211884_c1_g4	2540	181.556.491.757.011	0.0011659060000883	3.0E-89	vitamin D3 receptor A-like	P:GO:0050794; F:GO:0005488; P:GO:0009987
		212448_c1_g9	2704	205.876.632.729.864	0.00987570548782275	0.0	cadherin-related tumor suppressor-like	F:GO:0005509; C:GO:0016020; C:GO:0016021; P:GO:0007155; P:GO:0007156; C:GO:0005886
		212494_c4_g2	2598	217.211.079.195.528	8,16E+09	0.0	heat shock 75 mitochondrial-like	F:GO:0005524; F:GO:0051082; P:GO:0006457; P:GO:0006950
		212706_c5_g2	2825	232.242.410.660.868	0.000475539019063041	3.5E-68	follistatin-related 1	F:GO:0005509; F:GO:0008201; C:GO:0005576
		212912_c6_g13	556	538.289.081.834.067	5,54E+08	1.7E-85	diphthamide biosynthesis 2-like	P:GO:0017183
	Upregulated in Non Reproductive	196735_c0_g3	2008	-580.705.536.531.542	0.000717790387555743	3.8E-30	Dopamine receptor	P:GO:0007154; P:GO:0044700; F:GO:0004871
		197529_c0_g11	1769	-237.270.556.613.267	0.00116915540603387	4.3E-115	isoforms D E-like	C:GO:0016020; C:GO:0016021
		203468_c0_g1	3517	-441.540.934.166.374	0.00297465629469648	3,02E-130	multidrug resistance 1A-like	F:GO:0000166; F:GO:0016887; C:GO:0016020; P:GO:0006810
		205179_c1_g6	1877	-595.681.601.611.409	0.00123117918653153	7.5E-35	Transforming growth factor-beta-induced ig-h3	C:GO:0016020; C:GO:0016021
		205529_c4_g3	5582	-186.119.577.840.205	0.00978893847561384	9,88E-149	transcriptional enhancer factor TEF-1 isoform X2	0
		205772_c0_g24	308	-437.545.647.087.092	0.00674163878909597	9.0E-48	chaperonin containing TCP1 subunit 5 S	F:GO:0005524; C:GO:0005737; F:GO:0051082; P:GO:0006457
		206799_c1_g3	1865	-443.693.881.361.618	0.00227132959602301	1.4E-12	CD63 antigen	0
		208346_c2_g4	407	-511.728.656.737.872	0.00535611801171517	3.7E-9	ribonucleoside-diphosphate reductase	F:GO:0005524; F:GO:0004748; P:GO:0006260; P:GO:0055114
		210822_c0_g6	1525	-233.704.476.303.989	0.00509444340060381	1,24E-14	formin CG32138	F:GO:0003779; F:GO:0017048; P:GO:0030036
		212308_c1_g1	2113	-292.433.515.684.671	0.00294539509022163	2,95E-116	pappalysin-1-like	F:GO:0008270; P:GO:0006508; F:GO:0008237
A	Upregulated in Male	175720_c0_g2	472	642.575.996.615.803	0.001046634	7,32E-58	transcript antisense to ribosomal RNA	0
		183453_c1_g6	496	642.006.850.512.688	0.004643449	2,91E-29	sugar transporter STL1	0
	Upregulated in Non Reproductive	206390_c1_g4	311	-619.034.224.753.503	0.004643449	3.20E-14	lysosomal-associated transmembrane protein	C:GO:0016021
		208504_c2_g6	592	-605.410.547.343.974	0.004643449	3.40E-46	signal peptide, CUB and EGF-like domain-	F:GO:0005509
P	Upregulated in Male	175720_c0_g2	472	340.154.670.054.153	0.005911388	7,32E-58	transcript antisense to ribosomal RNA	0
		178264_c0_g13	317	389.172.885.707.627	0.007215549	2.30E-58	glycogen phosphorylase	0
		201170_c1_g10	430	622.536.415.137.616	0.009017072	9.80E-51	Diaphanous-like protein	F:GO:0003779; P:GO:0007015; F:GO:0017048; P:GO:0007049
		211853_c7_g19	790	552.270.091.128.396	0.001743639	3.00E-22	leucine-rich repeat transmembrane neuronal	P:GO:0018108; F:GO:0005524; F:GO:0004713; C:GO:0016020; C:GO:0016021; F:GO:0004672; P:GO:0016310; P:GO:0006468; F:GO:0016301
		211853_c7_g25	418	632.886.417.367.154	0.001883516	3.60E-23	tyrosine-protein kinase Src42A	P:GO:0018108; F:GO:0005524; F:GO:0004713; C:GO:0016020; C:GO:0016021; F:GO:0004672; P:GO:0016310; P:GO:0006468; F:GO:0016301
	Upregulated in Non Reproductive	181504_c0_g1	619	-644.778.980.957.667	0.00188351623490179	8.6E-12	PREDICTED: obscurin-like	0
		193764_c0_g12	326	-576.901.197.635.816	0.00591138752177324	2,91E-07	zinc finger and BTB domain-containing 17	F:GO:0003676; F:GO:0046872
		205772_c0_g24	308	-572.850.184.454.963	0.00948630458411057	9.0E-48	chaperonin containing TCP1 subunit 5 S	F:GO:0005524; C:GO:0005737; F:GO:0051082; P:GO:0006457
		208483_c0_g13	694	-561.804.270.154.586	0.00935816366422353	2,17E-85	excitatory amino acid transporter 3-like	C:GO:0016020; F:GO:0015293; P:GO:0006810
		211073_c2_g6	1523	-674.286.677.781.494	0.00061790884596273	2,14E-70	PREDICTED: obscurin	0
		144407_c0_g1	942	107.026.788.160.203	7,97E+06	8,41E-110	testis-specific serine threonine- kinase 1	F:GO:0005524; F:GO:0004674; P:GO:0006468
		163695_c0_g1	448	816.679.816.171.384	0.000369745183089735	1,18E-25	sperm-tail PG-rich repeat-containing 2-like	0
		169446_c1_g2	374	813.295.999.462.644	0.000501280885952236	6.5E-38	Histone-lysine N-methyltransferase PRDM9	F:GO:0005488; F:GO:0016740
		175720_c0_g2	472	559.082.873.424.366	0.00638579752915408	2.7E-14	transcript antisense to ribosomal RNA	0
		176698_c0_g2	784	670.827.758.959.081	0.00435701854769997	2.7E-24	rRNA promoter binding	0
		177071_c0_g3	820	615.262.352.967.929	0.00123887632126314	4,82E-85	adenylate kinase 9-like	F:GO:0005524; P:GO:0016310; F:GO:0019205; P:GO:0006139
		177833_c2_g3	403	663.216.269.204.049	0.00448123695196101	1.5E-10	protocadherin Fat 3-like	F:GO:0005509
		179431_c0_g4	1047	89.381.930.689.375	4,82E+09	3.2E-142	creatine kinase U-mitochondrial-like	F:GO:0000166; F:GO:0016772
		182063_c1_g4	1881	680.218.501.424.667	0.000132036907662178	5.4E-137	ATPase family AAA domain-containing 2B-like isoform	F:GO:0000166; F:GO:0005524
		182066_c0_g1	621	66.800.750.492.286	0.00446828010714909	3.0E-7	kinase domain containing (macronuclear)	F:GO:0016740
		183129_c0_g1	1344	101.003.152.538.807	2,15E+07	3,00E-121	testis-specific serine threonine- kinase 4-like	F:GO:0000166; C:GO:0005634; C:GO:0005737; F:GO:0004674; P:GO:0006468; P:GO:0035556
		183198_c2_g3	1841	10.453.843.584.209	7,48E+07	1,19E-75	cyclic nucleotide-binding domain-containing 2-like	0
		185652_c0_g1	2210	530.036.552.248.183	0.00839385009605524	2,00E-101	dynein heavy chain axonemal-like	0
		185706_c0_g2	1368	106.269.804.681.796	3,27E+07	4,75E-25	sperm motility kinase X	F:GO:0005524; F:GO:0004672; P:GO:0016310; P:GO:0006468; F:GO:0016301
		187109_c0_g1	821	73.975.468.945.248	0.000334405260264072	4.5E-67	Dynein heavy chain axonemal	F:GO:0003777; C:GO:0030286; P:GO:0007018
		187252_c0_g1	1223	619.880.593.653.851	0.000850303673463213	3.7E-66	coiled-coil domain-containing 178-like	0
		187645_c0_g3	449	549.749.418.494.226	0.00595402878028309	1,18E-08	sperm flagellar 1-like	0
		188849_c1_g3	1136	936.036.080.337.564	8,38E+08	6.9E-54	WD repeat-containing on Y chromosome-like	0
		188849_c1_g6	1650	103.613.237.660.167	1,22E+08	3.6E-42	WD repeat-containing on Y chromosome-like	0
		189320_c0_g3	2107	722.902.694.198.479	0.000100952116804516	1.4E-32	Deleted in malignant brain tumors 1	C:GO:0016020; F:GO:0005044; P:GO:0006898
		190274_c0_g2	980	590.699.772.560.007	0.00217387806937298	5.3E-35	Dynein heavy chain axonemal	0
		190541_c0_g9	507	545.968.370.552.792	0.00558454100005893	1.2E-16	cilia- and flagella-associated 53-like	P:GO:0003341
		190818_c1_g3	1057	678.075.089.739.431	0.00202375456894538	4,39E-83	kelch 10	0
		190818_c1_g4	1589	817.393.055.701.136	4,42E+09	0.0E0	kelch 10	0
		191209_c1_g2	1406	560.368.159.712.575	0.00358311184724237	2,50E-165	dynein regulatory complex subunit 7-like isoform X2	0
		191228_c0_g2	2240	537.359.442.081.867	0.00384128530863972	0.0	coiled-coil domain-containing 37-like isoform	0
		191523_c0_g4	1855	697.378.724.080.749	6,81E+08	1,86E-77	coiled-coil domain-containing 62-like isoform	0
		191704_c0_g1	1719	683.413.500.615.446	0.000203847850354665	1,49E-19	serine threonine- kinase ppk4-like	F:GO:0005524; P:GO:0090501; F:GO:0004672; F:GO:0004540; P:GO:0016310; P:GO:0006397; P:GO:0006468; F:GO:0016301
		191821_c0_g1	990	782.669.301.212.934	1,80E+09	1,40E-60	probable G- coupled receptor 157	F:GO:0004888; P:GO:0007165; C:GO:0016020
		192300_c0_g3	1577	584.898.541.852.883	0.00252760809215888	4,54E-56	lebercilin-like isoform X1	0
		192899_c0_g4	2782	976.423.706.022.656	3,40E+06	1,25E-04	golgin subfamily B member 1-like	0
		193315_c0_g1	1902	533.185.445.006.151	0.0042847580662995	2,85E-146	PREDICTED: protein FAM184A-like	0
		193943_c2_g1	1989	117.966.844.288.403	3,07E+05	1,06E-163	tektin-3-like isoform X1	0
		194502_c0_g6	414	697.575.968.486.279	0.00650818602352166	1,52E-16	sperm flagellar 2-like isoform X1	0
		194849_c0_g2	3366	683.792.057.946.328	0.000688602423503043	1.8E-74	WD repeat-containing on Y chromosome-like	0
		194849_c0_g3	1427	914.081.627.120.602	2,67E+09	1.6E-67	WD repeat-containing on Y chromosome	0
		195191_c1_g1	684	527.749.114.338.507	0.00266592484530237	1,05E-25	hydrocephalus-inducing -like	P:GO:0003341
		195379_c0_g5	709	584.307.883.481.488	0.0010008133109916	2,66E-116	dynein heavy chain axonemal-like isoform X1	F:GO:0003777; F:GO:0005509; C:GO:0030286; P:GO:0007018
		195927_c1_g5	437	838.281.206.716.289	0.000102822175145643	1,59E-24	glutamine-rich 2-like isoform X1	0
		195927_c1_g7	723	811.936.633.877.652	1,12E+09	1,78E-22	glutamine-rich 2-like isoform X1	0
		195927_c1_g9	329	649.374.082.156.628	0.00560887374278066	9,81E-27	nuclease subunit C isoform X5	0
		195949_c0_g2	2248	700.722.719.316.157	5,56E+09	0.0	BTB POZ domain-containing 16-like isoform	0
		196300_c0_g2	3016	493.929.423.673.421	0.00982445701302659	0.0	ankyrin repeat and EF-hand domain-containing 1-	F:GO:0005509
		196900_c2_g3	1432	641.456.926.106.353	0.00105865968250634	3,29E-71	synaptonemal complex 3	0
		197165_c1_g5	1653	670.956.674.964.241	0.000171911083751207	0.0	meiotic recombination DMC1 LIM15 homolog	C:GO:0005634; F:GO:0003677; F:GO:0005524; P:GO:0006281; F:GO:0008094; P:GO:0007131
		198010_c0_g1	571	706.546.405.323.984	0.00218903453783305	1,45E-08	HORMA domain-containing 1-like	0
		198503_c0_g1	442	604.976.539.038.075	0.00225468995503994	4,57E-49	cilia- and flagella-associated 58-like	0
		198683_c1_g1	899	773.269.515.313.572	0.000202293925971682	1,41E-94	outer dense fiber 3-like	0
		198724_c0_g4	1823	612.086.024.795.738	0.00427856029664961	1,26E-138	F-box only 47	0
		199059_c0_g2	399	592.076.919.645.671	0.00120804807761786	2,04E-48	creatine kinase U-mitochondrial-like	F:GO:0000166; F:GO:0016772
		199289_c1_g2	890	56.070.779.302.821	0.00416758202948689	1,50E-12	WD repeat-containing 87	0
		199494_c1_g2	406	752.377.707.032.246	0.00164515391114572	1,77E-03	axonemal 84 kDa isoform X1	0
		199879_c0_g2	3206	614.016.672.367.376	0.00536264545071617	2,25E-06	zona pellucida-binding 2 isoform X4	P:GO:0007339; C:GO:0016020; C:GO:0016021; C:GO:0005576
		199894_c0_g2	1775	863.279.842.053.248	8,59E+08	4,22E-114	leucine-rich repeat-containing 74B-like	0
		200046_c0_g1	2039	605.512.656.182.579	0.00085643677229565	1,01E-124	Coiled-coil domain-containing 87	0
		200427_c0_g4	2209	611.332.887.602.743	0.00090520884070768	6,41E-142	coiled-coil domain-containing 178-like	0
		201059_c0_g14	545	543.419.052.204.954	0.00644927457100538	1,23E-17	glutamate-rich 6-like	0
		201118_c0_g1	850	771.700.594.303.429	4,26E+09	5,15E-56	leucine-rich repeat soc-2-like	0
		201118_c0_g3	692	683.460.931.764.024	0.000161134646908698	3,87E-44	leucine-rich repeat-containing 1	0

MALE REPRODUCTIVE vs NON REPRODUCTIVE	F	Upregulated in Male	201604_c0_g1	2580	512.488.699.105.559	0.00748669257943635	0.0E0	Coiled-coil domain-containing 39	0
			201604_c0_g3	615	555.740.116.384.889	0.00518811180873932	2.0E-68	coiled-coil domain-containing 39-like	0
			202013_c0_g6	3922	497.196.830.647.704	0.00832646119724358	0.0	cilia- and flagella-associated 57-like	0
			202175_c1_g1	1416	100.846.840.252.662	1,60E+08	1.3E-90	Testis-specific serine threonine- kinase 1	F:GO:0016301
			202224_c0_g3	1545	54.508.434.805.342	0.00445115010397918	7.5E-26	leucine-rich repeat-containing 43-like	0
			202447_c0_g2	378	610.622.168.787.466	0.00085877176435586	6,04E-26	Sperm-associated antigen 17	0
			202588_c1_g1	690	892.408.738.929.725	8,55E+09	7,66E-07	circumsporozoite -	0
			202647_c0_g5	581	652.177.666.854.848	0.00142993883012642	4,14E-19	Leucine-rich repeat and IQ domain-containing 3	0
			203232_c0_g1	1130	599.207.530.242.081	0.0011225921064582	3,84E-165	dynein regulatory complex subunit 7-like	0
			204223_c0_g2	2727	557.053.084.020.889	0.00294882615940824	0.0	dynein heavy chain axonemal-	F:GO:0003777; P:GO:0007018
			204223_c0_g5	373	58.867.467.502.443	0.00178046417095114	2.5E-29	Dynein heavy chain axonemal	0
			204258_c0_g3	688	547.685.116.128.813	0.00396818796712813	2,73E-138	dynein heavy chain axonemal-	F:GO:0003777; F:GO:0005509; C:GO:0030286; P:GO:0007018
			204258_c0_g4	1542	697.025.010.714.375	2,32E+09	0.0	dynein heavy chain axonemal	F:GO:0003777; F:GO:0005509; C:GO:0030286; P:GO:0007018
			204468_c0_g10	364	723.141.310.092.982	0.00390707963664436	1,07E-39	leucine-rich repeat-containing 71-like isoform	0
			204625_c0_g4	2513	680.909.450.711.914	6,80E+09	0.0	EF-hand domain-containing family member	F:GO:0005509
			204843_c1_g1	2570	524.649.387.716.948	0.00651652372202861	0.0	EF-hand calcium-binding domain-containing 12-like	F:GO:0005509
			204867_c0_g5	1476	594.560.665.930.655	0.00730169062893089	1,34E-169	cilia- and flagella-associated 99-like	0
			205474_c0_g1	4023	548.678.916.183.504	0.0046858677600321	0.0	dynein beta ciliary	F:GO:0016887; F:GO:0005524; F:GO:0003777; C:GO:0030286; P:GO:0007018
			205600_c2_g6	3664	70.509.809.928.814	0.000231321576365773	2,20E-36	Serine threonine- kinase endoribonuclease IRE2	0
			206065_c0_g16	636	603.256.534.380.167	0.00251977594547695	1,48E-34	phosphatase 1 regulatory subunit 36-like	F:GO:0019902; P:GO:0010923
			206181_c1_g10	318	646.445.768.781.469	0.00573727899432901	3.0E-49	kinesin KIF9-like isoform X4	0
			206181_c1_g11	345	529.839.690.564.392	0.00384855472871788	2,46E-13	kinesin KIF9 isoform X1	F:GO:0005488
			206181_c1_g14	1019	597.730.343.100.417	0.00107199864505357	5,61E-161	kinesin KIF9	F:GO:0005524; F:GO:0003777; P:GO:0007018; C:GO:0005874; F:GO:0008017
			206219_c0_g5	619	808.657.146.429.933	9,94E+08	1,29E-20	speriolin -like	0
			206227_c1_g14	329	59.579.850.635.196	0.00779032707263389	1,05E-30	F-box only 36-like	0
			206374_c0_g3	603	603.707.502.923.948	0.00294957113817416	3,33E-31	coiled-coil domain-containing 87-like isoform	0
			207028_c0_g1	1269	61.989.573.820.821	0.00956988897139085	1,47E-02	ATP-dependent DNA helicase Q4	F:GO:0097159; F:GO:1901363; C:GO:0044424
			207141_c1_g7	913	568.747.247.682.597	0.00402855568142149	2,54E-102	coiled-coil domain-containing 42 homolog	0
			207367_c2_g6	311	520.375.236.943.674	0.00668956720251513	4,57E-12	O(6)-methylguanine-induced apoptosis 2-like	0
			207565_c1_g4	1992	62.728.853.188.298	0.000850874484812126	1,16E-42	WD repeat-containing 87	0
			207715_c0_g3	1493	528.689.697.620.435	0.00673939469180807	3,29E-70	sperm-associated antigen 17-like	0
			207723_c1_g9	862	529.006.903.817.985	0.00488026098037384	3,10E-37	ubiquitin carboxyl-terminal	P:GO:0016579; C:GO:0016020; C:GO:0016021; P:GO:0006511; F:GO:0016787; F:GO:0036459
			207854_c0_g3	1554	545.935.136.886.039	0.0059027633740682	1,92E-147	ankyrin repeat and MYND domain-containing 1-like	0
			208171_c0_g1	1585	579.866.765.971.475	0.00162669176016733	0.0E0	AMY-1-associating expressed in testis 1	0
			208299_c0_g1	586	817.049.249.816.173	8,55E+09	3,86E-84	dynein heavy chain axonemal-like	F:GO:0016887; F:GO:0005524; F:GO:0003777; C:GO:0030286; P:GO:0007018
			208339_c0_g2	1642	727.977.665.011.782	0.000164537302887813	0.0E0	Dynein heavy chain axonemal	F:GO:0016887; F:GO:0005524; F:GO:0003777; C:GO:0030286; P:GO:0007018
			208339_c1_g2	3522	734.869.852.958.847	0.00050166516058258	0.0	dynein heavy chain axonemal	F:GO:0016887; F:GO:0005524; F:GO:0003777; C:GO:0030286; P:GO:0007018
			208692_c0_g2	3338	685.751.182.009.631	0.00398231109822868	1,21E-07	tripartite motif-containing 3-like	F:GO:0008270; F:GO:0046872; C:GO:0005622
			208770_c2_g1	3185	864.409.413.829.734	1,85E+09	3,35E-45	kelch 9	0
			208901_c1_g2	2798	573.862.477.055.166	0.002477139604022	0.0E0	IQ and ubiquitin-like domain-containing	0
			208934_c3_g3	361	690.659.928.009.181	0.000693867820184624	3.7E-23	stabilizer of axonemal microtubules 2 isoform X4	F:GO:0008017
			209178_c0_g2	1093	515.843.369.943.002	0.00468148399202929	4,97E-54	dynein heavy chain axonemal-like isoform X1	F:GO:0016887; F:GO:0005524; F:GO:0003777; C:GO:0030286; P:GO:0007018
			209178_c1_g5	745	751.701.820.050.248	6,28E+09	3.4E-55	dynein heavy chain axonemal-like	0
			209416_c1_g9	1614	721.163.642.568.508	0.00053098408534715	5,26E-10	trichohyalin isoform X4	0
			209819_c0_g1	6726	571.227.748.251.032	0.00124786247506025	0.0E0	Dynein heavy chain axonemal	F:GO:0005524; F:GO:0003777; C:GO:0030286; P:GO:0007018
			209970_c1_g2	2148	606.155.263.639.424	0.000703410719297412	1,20E-116	WD repeat-containing	0
			209970_c1_g3	415	621.282.356.259.238	0.00061501008012481	2,57E-11	WD repeat-containing KIAA1875-like	0
			210013_c1_g1	3204	577.626.172.751.303	0.00221180991368804	0.0	hydrocephalus-inducing -like	P:GO:0003341
			210435_c0_g1	1706	563.509.032.996.266	0.00178453951872967	0.0	HEAT repeat-containing 4-like	0
			210435_c1_g1	1968	588.109.176.581.145	0.00160838798357989	8,18E-126	HEAT repeat-containing 4-like	0
			210475_c1_g2	3439	598.153.583.496.544	0.00114289365472438	1,82E-119	adenylate kinase 9 isoform X6	0
			210653_c1_g1	1067	598.196.924.578.305	0.00340138183631654	3,00E-112	tetratricopeptide repeat 25-	0
			210703_c0_g4	1837	51.076.394.235.437	0.00681254792721783	8,40E-122	IQ domain-containing D-like	0
			210801_c1_g9	3914	568.998.005.299.391	0.00271962017168926	1,60E-85	FAM78B-like isoform X1	0
			210824_c1_g1	323	617.347.551.697.508	0.00384855472871788	1,65E-49	IQ domain-containing G-like	0
			210893_c1_g14	1530	583.940.383.682.204	0.00200498591365559	9,81E-115	coiled-coil domain-containing 74B isoform X1	0
			211136_c1_g5	1763	481.353.932.935.953	0.00688846288491104	0.0E0	Dynein heavy chain axonemal	F:GO:0000166
			211271_c2_g3	3078	62.124.365.116.931	0.000322659054052294	1,18E-64	Dynein heavy chain axonemal	F:GO:0003777; F:GO:0005509; C:GO:0030286; P:GO:0007018
			211271_c2_g7	591	917.773.404.471.716	3,00E+09	2.9E-69	Dynein heavy chain axonemal	0
			211329_c2_g2	1723	906.470.689.619.676	1,18E+09	3,62E-13	leucine zipper 1 isoform X2	0
			211329_c2_g6	2632	702.600.815.601.971	0.000176582040321036	3,97E-12	nuclear mitotic apparatus 1-like isoform X2	0
			211599_c3_g2	1038	72.639.325.807.382	0.000121683459193696	1,92E-25	tubulin monoglycylase TTLL3-like isoform X5	0
			211599_c3_g4	3538	702.979.049.997.463	0.000144975933456708	0.0	monoglycylase TTLL8 isoform X3	0
			211649_c1_g1	2042	550.937.314.542.295	0.00248513343227684	1,76E-63	sex determining Fem-1 like	C:GO:0016020; C:GO:0016021
			211785_c0_g8	3954	540.050.491.938.876	0.00535963936800357	0.0	ankyrin repeat domain-containing 26-like isoform	0
			211869_c1_g1	3169	955.408.463.271.819	3,63E+08	3,44E-34	regulator of G- signaling -like	0
			211869_c1_g6	435	968.559.004.286.471	6,91E+08	3,93E-08	regulator of G- signaling -like	C:GO:0005737; F:GO:0005096; C:GO:0005886; P:GO:0043547
			212379_c1_g1	1618	688.664.063.191.689	0.00204825738044663	0.0	dynein heavy chain axonemal-like	0
			212379_c1_g2	4712	734.364.805.504.686	0.00147628970137323	0.0	dynein heavy chain axonemal-like	0
			212555_c0_g3	1925	852.007.258.068.384	0.000205536148905648	4,96E-13	tax1-binding 1 homolog B-like	0
			212665_c1_g1	4358	584.914.483.822.621	0.00210188654859862	0.0	dynein beta ciliary-like	F:GO:0016887; F:GO:0005524; F:GO:0003777; C:GO:0030286; P:GO:0007018
			212665_c1_g3	6759	520.787.594.884.542	0.00508222150710734	0.0	dynein beta ciliary	F:GO:0016887; F:GO:0005524; F:GO:0003777; C:GO:0030286; P:GO:0007018
			212665_c1_g5	430	586.219.718.840.625	0.00224865185634156	8,84E-73	dynein beta ciliary-like isoform X2	F:GO:0016887; F:GO:0005524; F:GO:0003777; C:GO:0030286; P:GO:0007018
			212730_c4_g5	2135	536.222.234.389.871	0.00404029289093467	0.0	armadillo repeat-containing 4 isoform X1	C:GO:0044424
			212918_c0_g2	14338	490.214.428.998.928	0.00982445701302659	0.0E0	Dynein heavy chain axonemal	0
			212918_c0_g3	964	593.892.446.720.004	0.00335856896808498	6.5E-89	Dynein heavy chain axonemal	F:GO:0000166; P:GO:0006928; C:GO:0030286; F:GO:0017111
			212923_c2_g1	10657	459.707.462.935.967	0.00813369026201676	0.0	dynein heavy chain axonemal-like	F:GO:0016887; F:GO:0005524; F:GO:0003777; C:GO:0005858; P:GO:0003341
			212945_c4_g3	2547	540.809.007.178.306	0.002477139604022	0.0	hydrocephalus-inducing -like	P:GO:0003341
			212945_c4_g5	1382	580.117.401.719.413	0.00107815193363437	0.0	Hydrocephalus-inducing -like	P:GO:0003341
			212972_c3_g2	2338	694.988.666.802.678	0.000142635487143221	1,31E-67	interaptin-like isoform X1	0
			213045_c6_g2	8853	524.998.127.681.083	0.0028055616634178	0.0	dynein heavy chain axonemal isoform X5	F:GO:0016887; F:GO:0005524; F:GO:0003777; C:GO:0030286; P:GO:0007018
			185777_c0_g1	644	-749.002.830.702.302	0.000859471	1,50E-17	Pancreatic Triacylglyceride Lipase	C:GO:0005576; F:GO:0052689
			186767_c0_g1	1259	-663.441.644.811.071	0.005708161	3,49E-63	soluble guanylate cyclase gcy-33-like isoform X3	F:GO:0016829; P:GO:0044699; P:GO:0009987
			188868_c1_g2	1735	-532.553.468.505.551	0.007703887	2,94E-38	leucine rich repeat	0
			191245_c0_g1	1958	-678.416.484.188.642	0.00390708	1,74E-122	inactive ubiquitin carboxyl-terminal hydrolase MINDY-	0
			191926_c0_g10	2187	-82.565.131.294.015	7,48E+08	5,71E-71	solute carrier organic anion transporter family	P:GO:0006811; C:GO:0016020; C:GO:0016021; P:GO:0006810; C:GO:0005886; F:GO:0005215
			194234_c0_g5	458	-601.197.052.017.634	0.003003151	3.90E-07	phosphatidylinositol 4-kinase beta-like isoform X2	P:GO:0048015; P:GO:0046854; P:GO:0016310; F:GO:0016740; F:GO:0016301; C:GO:0005622
			195762_c0_g1	2835	-684.585.694.275.425	0.003352535	0.00E+00	multidrug resistance-associated 1-like	0
			197328_c0_g4	591	-634.661.740.072.635	0.008759046	4,86E-17	gamma-glutamyl cyclotransferase venom	P:GO:0006750; F:GO:0003839
			197882_c0_g1	3005	-780.680.603.196.909	0.000168748	9,32E-67	sodium-coupled monocarboxylate	P:GO:0055085; C:GO:0016020; C:GO:0016021; P:GO:0006810; F:GO:0005215

		Upregulated in Non Reproductive	199174_c2_g5	677	-669.589.682.875.421	0.001626692	3,00E-24	solute carrier family 28 member 3-like isoform X2	0
			199400_c0_g4	2325	-571.997.783.234.794	0.008753833	1,06E-58	proton-coupled folate transporter-like	0
			200067_c0_g1	1762	-689.016.087.132.211	0.00390708	2.00E-127	epithelial chloride channel -like	C:GO:0031226
			200925_c0_g5	874	-669.887.361.991.809	0.003980417	4,29E-53	sodium- and chloride-dependent glycine	C:GO:0016020; F:GO:0015293; P:GO:0006810
			201834_c0_g6	1123	-706.385.328.949.038	0.003003151	2,63E-10	C1q and tumor necrosis factor-related 2	0
			201834_c0_g7	474	-743.643.960.001.044	0.001269795	7.70E-09	C1q and tumor necrosis factor-related 2	0
			202172_c0_g2	1478	-5.663.374.273.822	0.005362645	2,74E-36	major facilitator superfamily domain-	C:GO:0016020; C:GO:0016021
			203468_c0_g1	3517	-66.201.073.176.643	0.000594723	7.00E-131	multidrug resistance partial	F:GO:0000166; F:GO:0016887; C:GO:0016020; P:GO:0006810
			205179_c1_g6	1877	-918.803.479.729.415	2,12E+08	7.50E-35	Transforming growth factor-beta-induced ig-h3	C:GO:0016020; C:GO:0016021
			205772_c0_g24	308	-649.396.276.362.729	0.006402835	1,21E-45	chaperonin containing TCP1 subunit 5 S	F:GO:0005524; C:GO:0005737; F:GO:0051082; P:GO:0006457
			205840_c0_g3	3623	-526.563.449.175.332	0.008151797	0.0	laminin subunit alpha-like isoform X2	P:GO:0045995; P:GO:0007155; P:GO:0030155; P:GO:0030334; F:GO:0005102
			205945_c0_g6	1472	-554.789.900.884.411	0.007815678	4,32E-11	hypothetical protein CAPTEDRAFT_217914	0
			206694_c1_g2	2233	-493.876.288.077.565	0.007268576	0.0	F-box WD repeat-containing 7-like isoform	0
			206782_c1_g3	1544	-613.170.276.630.949	0.006385798	3.30E-92	phospholipid scramblase 2-like	0
			206915_c3_g1	381	-642.448.546.180.406	0.008629566	4.30E-18	isoleucyl-tRNA synthetase	F:GO:0000166; P:GO:0006428; P:GO:0006418; F:GO:0005524; F:GO:0004812; F:GO:0004822; C:GO:0005737; P:GO:0006450; F:GO:0002161
			207190_c2_g5	1375	-679.539.876.866.016	0.003121396	1,49E-94	solute carrier family 23 member 1-	P:GO:0055085; C:GO:0016020; C:GO:0016021; P:GO:0006810; F:GO:0005215
			207280_c0_g4	877	-658.392.865.105.857	0.002173878	5,55E-68	phospholipase membrane-associated-like	F:GO:0016788
			208019_c0_g2	1626	-583.045.121.784.736	0.006994263	2.30E-16	human enterokinase EC	C:GO:0016020; C:GO:0016021
			208220_c1_g12	454	-720.541.799.746.139	0.002115915	4,70E-04	Trophoblast Kunitz domain 1	0
			208483_c0_g2	648	-639.202.155.270.216	0.003525542	7,81E-11	excitatory amino acid transporter	P:GO:0006835; P:GO:0055085; C:GO:0016020; F:GO:0017153; C:GO:0016021; F:GO:0015293; P:GO:0006810
			210669_c1_g2	2160	-660.663.958.249.931	0.000928298	1,01E-21	collagen alpha-1(XII) chain-like	C:GO:0005581
			210951_c4_g12	1180	-585.736.530.016.111	0.003317158	7,57E-164	Serotonin transporter	C:GO:0016020; F:GO:0015293; P:GO:0006810
			210951_c4_g15	1102	-629.347.106.664.235	0.001238876	1,29E-68	sodium- and chloride-dependent glycine	P:GO:0006810
