# Supplementary material for: Delegating Sex: Differential Gene Expression in Stolonizing Syllids Uncovers the Hormonal Control of Reproduction
Source: Genome Biol Evol. 2018 Dec 11;11(1):295–318. doi: 10.1093/gbe/evy265 (PMC6350857; doi:10.1093/gbe/evy265)
Supplement: Supplementary Data [file evy265_supp.zip › SuppFile_S7_REFTOTREP.pdf]

Comparison	Tissue	Regulation	Contig ID	Contig Length	log2FoldChange	padj	e-Value	Description	GO IDs
FEMALE REPRODUCTIVE vs MALE REPRODUCTIVE	P	Upregulated in Female	c103739_g1	1581	580.598.859.904.818	0.00119118	2.40E-37	Signal peptide, CUB and EGF-like domain-containing protein 3	F:GO:0005509; C:GO:0016020; C:GO:0016021
			c114522_g2	957	650.650.461.547.792	0.000285753	2.40E-14	Signal peptide, CUB and EGF-like domain-containing 1	C:GO:0016020; C:GO:0016021
			c121841_g3	1243	585.577.637.963.359	0.000721302	4.90E-83	chitotriosidase	F:GO:0016787
			c121868_g1	2724	637.899.683.473.854	0.000241487	3.60E-46	ovochymase 2	P:GO:0006508; C:GO:0016020; F:GO:0008236; F:GO:0004252; F:GO:0008233; F:GO:0016787
			c122352_g1	1188	652.189.952.823.458	0.000285753	9.20E-31	Fibrillin	F:GO:0008270; F:GO:0046872; C:GO:0016020; P:GO:0006508; F:GO:0008233; F:GO:0004222; F:GO:0008237; F:GO:0016787
			c122900_g1	3555	516.258.403.452.214	0.006402108	1.30E-15	MAM and LDL-receptor class A domain-containing protein 2-like [Lingula anatina]	0
			c125074_g2	2340	573.111.451.464.598	0.003041689	1.50E-33	MAM and LDL-receptor class A domain-containing protein 2-like [Lingula anatina]	F:GO:0008270; F:GO:0046872; C:GO:0016020; P:GO:0006508; F:GO:0008233; F:GO:0004222; F:GO:0008237; F:GO:0016787
	F	Upregulated in Female	c108460_g1	1174	612.954.442.578.782	0.005659409	1,33E-35	Williams-Beuren syndrome chromosomal region 27 -like	0
			c114000_g1	1065	6.074.156.259.487	0.005907728	2,02E-34	collagen alpha-1(XII) chain-like	F:GO:0005509
			c115902_g1	875	632.257.012.309.085	0.003018462	1,31E-22	leucine-rich repeat-containing 15-like	P:GO:0035385; F:GO:0048495; C:GO:0005615; P:GO:0050770; F:GO:0008201; C:GO:0005578; P:GO:0048846
			c118765_g2	346	638.481.000.211.011	0.003018462	4.20E-11	leucine Rich repeat-containing domain	C:GO:0016020; C:GO:0016021
			c119790_g2	2245	653.845.907.088.769	0.002406208	4,55E-104	collagen alpha-1(XII) chain-like	0
			c121380_g1	461	587.838.149.502.974	0.006445059	1.10E-09	Pre-B-cell leukemia transcription factor 4	0
			c122147_g2	723	739.080.944.733.899	0.000660477	9,57E-61	cytoplasmic polyadenylation element-binding 1-like isoform X1	F:GO:0000166; F:GO:0003676
			c122147_g3	2166	732.445.038.335.192	0.000660477	4,33E-106	cytoplasmic polyadenylation element-binding 1-like isoform X2	0
			c122584_g1	687	610.776.559.586.221	0.005194172	6.00E-13	oocyte-type linker histone B4	0
			c123085_g2	1388	638.196.547.280.565	0.003630261	7.60E-40	ascorbate peroxidase-like	0
			c125021_g1	3895	665.343.235.899.155	0.00205909	1.10E-76	vitellogenin	P:GO:0006869; F:GO:0005319
			c125957_g1	1137	645.959.908.628.437	0.003018462	2,00E-27	Ovochymase-1	0
			c126797_g1	2167	59.858.370.779.415	0.00651554	1,12E-07	kelch motif	F:GO:0008270; F:GO:0046872; C:GO:0005622
			c127593_g2	3048	635.581.400.831.441	0.003488699	4,82E-64	Collagen alpha-5(VI) chain	F:GO:0005509
			c127707_g2	4028	677.045.066.330.038	0.001386737	1.80E-25	vitellogenin	0
			c127954_g1	424	646.714.645.376.674	0.002873641	5.00E-14	L-rhamnose-binding lectin CSL3-like	F:GO:0030246
			c128190_g1	3491	653.236.515.758.121	0.002384001	3,03E-108	sodium-dependent multivitamin transporter isoform X2	C:GO:0016020; P:GO:0006810
			c128630_g1	961	582.972.868.038.154	0.008390271	2,68E-17	solute carrier organic anion transporter family member 2B1	P:GO:0006811; C:GO:0016020; C:GO:0016021; P:GO:0006810; C:GO:0005886; F:GO:0005215
			c130071_g1	2454	591.241.356.673.039	0.005941965	9.00E-08	Macrophage asialoglyco -binding 1	F:GO:0030246
			c131435_g1	3179	714.524.983.386.962	0.000953002	3,00E-132	tyrosine-protein kinase Src42A	0
			c131808_g1	2535	628.345.914.539.873	0.004205335	6.60E-80	fibrillin-1-like, partial	0
			c132573_g1	773	610.955.274.906.762	0.005517017	1,03E-09	probable chitinase 3	0
			c133893_g3	1948	690.298.967.304.444	0.001372311	3,25E-99	Ribosome biogenesis regulatory	F:GO:0005509
		Upregulated in Male	c104096_g4	1030	-604.371.614.227.839	0.00651554	2,44E-108	testis-specific serine threonine- kinase 1	0
			c108778_g1	915	-588.470.519.658.584	0.007986778	5.00E-45	kelch 10-like	0
			c110345_g1	1949	-622.812.169.142.217	0.005025261	3,75E-164	tektin-3-like isoform X1	0
			c111044_g1	742	-58.674.723.613.451	0.009253791	8,71E-08	ATP-dependent RNA	0
			c111623_g1	1319	-604.511.798.213.067	0.00651554	1,50E-01	sperm motility kinase X	F:GO:0005524; F:GO:0004672; P:GO:0006468
			c112851_g1	888	-684.603.878.050.735	0.001372311	7.60E-27	glycine receptor subunit alpha-3-like	P:GO:0006811; P:GO:0034220; C:GO:0016020; C:GO:0016021; C:GO:0030054; F:GO:0005230; C:GO:0045202; P:GO:0006810; C:GO:0005886
			c112883_g3	233	-598.277.800.891.773	0.00651554	6,93E-11	fibropellin-1-like isoform X10	0
			c115193_g2	1311	-626.453.315.685.298	0.004769017	5,31E-122	testis-specific serine threonine- kinase 4-like	0
			c117134_g2	1453	-647.965.129.802.588	0.002831344	3.20E-126	Monocarboxylate transporter 10	0
			c118060_g1	1065	-623.433.215.704.256	0.004970695	1.50E-14	leucine-rich repeat-containing FAM211A-like	0
			c118334_g2	502	-610.751.991.918.382	0.005659409	1.00E-87	creatine kinase, mitochondrial	F:GO:0005524; P:GO:0016310; F:GO:0016301
			c118456_g3	1274	-612.054.681.245.958	0.004769017	2,97E-33	adenylate cyclase type 10-like	F:GO:0016849; P:GO:0035556; C:GO:0005622; P:GO:0009190
			c118531_g2	1291	-684.956.049.687.897	0.001372311	9.80E-06	calcineurin subunit B type 2	0
			c119087_g1	701	-620.742.630.920.811	0.00493182	2.70E-75	meiotic recombination DMC1 LIM15 homolog, partial	0
			c119559_g1	251	-615.279.600.246.916	0.005194172	3.00E-56	tubulin beta-4B chain	0
			c119760_g2	677	-610.097.137.458.131	0.005030657	4.50E-09	PREDICTED: aquaporin-8-like	0
			c119812_g1	586	-608.719.163.925.867	0.005983914	1,21E-12	kelch diablo	0
			c119980_g3	451	-721.185.989.350.552	0.000660477	1.00E-17	carbonic anhydrase 12 precursor	0
			c121080_g1	2570	-582.098.468.465.284	0.00651554	1.70E-33	Deleted in malignant brain tumors 1	C:GO:0016020; F:GO:0005044; P:GO:0006898
			c121154_g1	712	-593.495.840.702.028	0.005165969	1.60E-39	EF-hand calcium-binding domain-containing 9-like	0
			c121506_g1	207	-592.837.195.340.443	0.005659409	4.20E-12	BTB POZ domain-containing 16-like isoform X2	0
			c121935_g2	2509	-564.091.929.737.176	0.00651554	3.90E-140	two pore calcium channel 1-like	0
			c121980_g3	1104	-635.307.617.031.239	0.0039063	8.00E-94	outer dense fiber 3-like	0
			c122717_g1	1565	-658.426.690.070.261	0.002125198	2,04E-14	amyotrophic lateral sclerosis 2 chromosomal region candidate gene 11	0
			c123333_g1	2848	-708.109.967.875.405	0.000806867	0.0	dynein heavy chain axonemal-like	0
			c123534_g1	2054	-563.953.799.415.764	0.008089422	0.00E+00	HORMA domain-containing 1-like	0
			c123750_g1	1625	-599.031.197.867.235	0.003567001	5.80E-87	cation channel sperm-associated 3-like	0
			c124672_g2	1269	-59.825.004.816.099	0.006445059	1,04E-39	fibrillin-2-like isoform X38	0
			c125809_g1	2163	-596.191.682.259.503	0.0039063	2,40E-126	adenylate cyclase type 10-like	F:GO:0016849; P:GO:0035556; C:GO:0005622; P:GO:0009190
			c126473_g1	2320	-610.145.640.895.197	0.004769017	3,34E-142	isocitrate dehydrogenase [NADP] cytoplasmic-like	F:GO:0005509
			c127028_g2	755	-599.994.752.654.467	0.006684702	2,05E-42	leucine-rich repeat-containing 74B-like	0
			c128039_g1	2526	-604.369.498.620.079	0.006478039	0.0	choline transporter 1 isoform X2	0
			c128206_g4	251	-627.234.976.769.725	0.004175699	7,29E-03	calcium-binding and coiled-coil domain-containing 1-like	0
			c128910_g2	1053	-674.257.902.962.411	0.001372311	1.40E-171	dynein heavy chain 5, axonemal-like, partial	0
			c130152_g1	2429	-621.872.170.715.459	0.005025261	1,34E-76	F-box only 39	0
			c130544_g1	1209	-664.352.858.588.492	0.002067647	1.30E-16	Brain-specific angiogenesis inhibitor 2	C:GO:0016020; C:GO:0016021
			c130856_g1	2935	-604.366.879.328.007	0.005865907	2.10E-81	zinc finger	0
			c130943_g2	1937	-60.819.562.188.369	0.005419599	2.20E-98	Histone-lysine N-methyltransferase PRDM9	0
			c131365_g1	4258	-582.478.442.822.678	0.006953593	1,68E-54	receptor-type tyrosine- phosphatase eta-like isoform X1	F:GO:0004721; F:GO:0004725; C:GO:0016020; P:GO:0006470; C:GO:0016021; F:GO:0016791; P:GO:0016311; P:GO:0035335; F:GO:0016787
			c131565_g3	653	-622.678.866.831.787	0.00436283	1.90E-11	DC-STAMP domain-containing 2-like	0
			c131847_g2	2508	-586.683.560.934.403	0.00651554	3,58E-12	nuclear mitotic apparatus 1-like isoform X2	0
			c132140_g1	3936	-641.448.251.687.119	0.002013336	5,43E-19	fibrous sheath-interacting 2	0
			c132709_g1	3980	-644.430.991.170.123	0.003186767	9.30E-50	WD repeat-containing on Y chromosome-like	P:GO:0055085; C:GO:0016020; C:GO:0016021
			c132820_g1	4288	-590.889.142.927.548	0.005865907	2,91E-133	WD repeat-containing 87	0
			c133022_g2	2156	-582.267.458.727.308	0.00605884	4.50E-39	sushi, nidogen and EGF-like domain-containing 1	0
			c133197_g1	4049	-618.000.390.040.996	0.002538364	1.20E-61	GBR-2A protein	0
			c133570_g1	306	-627.498.419.733.851	0.003290505	3,73E-12	cation channel sperm-associated 4-like	0
			c133602_g1	4691	-719.024.235.924.639	0.000660477	0.00E+00	neurogenic locus Notch -like	0
			c134319_g1	6378	-693.459.765.919.311	0.001135688	0.0	dynein heavy chain axonemal-like	0
			c134319_g2	1778	-625.460.929.852.748	0.00462824	0.00E+00	dynein heavy chain 8, axonemal-like, partial	0
			c134507_g1	6795	-612.215.661.174.436	0.00385621	7.10E-55	dentin sialophospho -like	0
			c134733_g1	2179	-604.480.804.722.079	0.00385621	2,23E-95	fibropellin-1-like isoform X14	0
			c134733_g2	3362	-550.958.517.935.305	0.007121611	4.70E-07	tolloid 1-like	0
			c134852_g1	2528	-647.866.431.087.141	0.002876222	1.20E-12	fibrous sheath-interacting 2	0
			c102789_g1	380	375.382.630.895.185	5,24E+01	1,15E-26	ras GTPase-activating -binding 1	F:GO:0097159; F:GO:1901363
			c103970_g2	587	194.873.206.595.554	0.007964079	9.20E-50	L27a	0
			c104239_g1	1189	29.423.361.503.357	0.007837637	4,22E-117	jmjC domain-containing 8-like	0
			c108985_g1	848	433.038.437.687.346	5,44E+00	2,94E-15	shisa-3 homolog	C:GO:0016020; C:GO:0016021
			c109691_g1	893	281.728.437.482.425	5,99E+09	5.20E-50	Small nuclear ribonucleo Sm D3	0
			c110036_g1	656	519.682.621.401.971	3,71E+07	3.50E-08	suppressor of tumorigenicity 14	0
			c110171_g7	422	287.174.525.958.718	0.006682091	2.70E-50	fatty acid desaturase 2-like	0
			c110813_g1	1117	303.419.519.233.785	0.005077183	4,03E-25	alpha-1D adrenergic receptor-like	F:GO:0004930; P:GO:0007186; P:GO:0007165; C:GO:0016020; C:GO:0016021; F:GO:0004871
			c111092_g1	681	231.120.708.012.886	0.001925668	3.90E-75	histone-arginine methyltransferase CARM1-like isoform X4	0
			c111102_g1	606	220.667.436.524.139	0.004862556	2.10E-62	ribosomal rpl24	C:GO:0005840
			c111174_g2	968	224.446.110.722.488	0.002005335	3.10E-98	Calmodulin	F:GO:0005509
			c111506_g3	597	249.345.966.764.158	2,62E+09	7,00E-32	seleno T2-	0
			c111715_g4	551	248.428.713.260.337	0.007557614	5.70E-23	ruvB-like 1-like	C:GO:0005634; F:GO:0005524; P:GO:0006281; F:GO:0043141; P:GO:0006351; P:GO:0032508; P:GO:0006355
			c112391_g1	1314	355.472.678.841.827	5,03E+09	1,34E-67	fructose-2,6-bisphosphatase TIGAR	0
			c112468_g1	1615	208.397.968.913.697	0.002947886	0.0	ryanodine receptor isoform X2	F:GO:0005219; F:GO:0005509; C:GO:0016021; P:GO:0051209; C:GO:0005622
			c112786_g2	623	25.614.930.256.388	0.004732939	3.60E-39	39S ribosomal L48, mitochondrial-like	0
			c112803_g1	714	293.487.539.641.098	0.002947886	7.40E-11	Neuroglobin, partial	0
			c112959_g1	1140	349.054.129.795.205	0.003682931	3.10E-30	NDUFAF4 homolog	0
			c113042_g1	558	372.004.172.164.789	0.000243863	5,55E-60	sodium- and chloride-dependent glycine transporter 1-like	0
			c113243_g4	833	312.662.161.781.032	6,32E+05	2.00E-104	Trpc4-associated protein	0
			c113358_g2	372	50.991.451.832.949	8,33E+06	3.10E-51	ribosomal rps19	F:GO:0003735; C:GO:0005840; P:GO:0006412
			c113529_g1	967	334.842.085.082.519	0.000240418	1.90E-79	NADH dehydrogenase subunit 1	0
			c113650_g2	596	349.621.857.577.685	2,17E+09	1.60E-21	28S ribosomal S16, mitochondrial	F:GO:0003735; P:GO:0032543; C:GO:0005763
			c113987_g3	1240	382.981.265.799.066	6,33E+06	9.10E-25	rRNA maturation factor homolog	0
			c113996_g4	472	25.914.879.641.521	4,02E+09	6,05E-12	nascent polypeptide-associated complex subunit	0
			c114223_g6	850	21.874.869.848.111	0.003028962	1.10E-44	ribosomal rpl34	F:GO:0003735; C:GO:0005840; P:GO:0006412
			c114324_g1	1698	343.358.823.794.018	0.000765161	1,05E-18	mammalian ependymin-related 1-like	0
			c114338_g1	2080	236.464.922.114.426	0.001151011	1,56E-149	3 (2),5 -bisphosphate nucleotidase 1-like isoform X1	P:GO:0046854
			c114371_g1	1884	383.581.860.273.357	6,16E+08	0.00E+00	beta-catenin	P:GO:0007165; P:GO:0034333; F:GO:0004871
			c114428_g1	1345	296.878.474.960.796	1,19E+09	2.70E-09	glutenin, high molecular weight subunit PW212-like	0
			c114463_g1	853	317.116.716.522.675	0.000472719	1.40E-45	glutathione S-transferase theta-1-like	0
			c114561_g4	846	271.959.794.494.302	2,44E+09	1.40E-91	alpha-tubulin	0
			c115173_g1	741	30.625.866.883.748	0.003230328	1,50E-28	RPA-interacting A-like isoform X1	0
			c115503_g2	710	172.109.803.943.176	0.009799652	1.80E-77	ribosomal rpl21	F:GO:0003735; C:GO:0005840; P:GO:0006412
			c115668_g3	458	221.570.250.889.804	5,14E+09	7.20E-44	40S ribosomal S25 isoform X1	0
			c115673_g1	228	375.420.254.921.104	1,65E+06	2.60E-07	zinc finger and btb domain containing	0
			c115722_g1	1396	25.661.011.343.408	0.006360219	4,32E-104	far upstream element-binding 1-like isoform X3	0
			c115736_g1	267	298.989.615.830.784	0.001917753	2,73E-34	lipoamide acyltransferase component of branched-chain alpha-keto acid dehydrogenase mitochondrial isoform X2	0
			c115750_g2	1149	308.603.497.562.658	4,62E+09	0.00E+00	Adenosylhomocysteinase A	0
			c115818_g1	1137	304.520.265.732.696	0.008958234	1,94E-118	transmembrane 144 isoform X3	C:GO:0016020
			c116031_g2	953	253.938.814.431.778	0.000633117	1,28E-60	TM2 domain-containing CG10795-like	C:GO:0016020
			c116289_g1	922	272.891.143.794.754	0.000460243	7,60E-138	myosin heavy	F:GO:0000166
			c116471_g4	568	157.266.116.905.291	0.007387901	2.70E-51	Small nuclear ribonucleo E	0
			c116495_g1	656	17.035.954.883.499	0.008085614	6.80E-15	beta-1,4 N-acetylgalactosaminyltransferase 2-like	0
			c116530_g3	498	234.121.930.133.357	0.002988631	6,65E-33	probable small nuclear ribonucleo G	0
			c116554_g1	880	277.727.987.740.985	0.001031031	4,08E-14	serum paraoxonase arylesterase 1-like isoform X2	0
			c116647_g1	1332	269.807.029.458.672	5,88E+08	2.40E-79	glutathione peroxidase 1	0
			c116754_g2	666	23.779.598.431.281	6,05E+08	3,60E-101	YTH domain-containing family 2-like isoform X2	F:GO:0003723
			c116935_g1	886	258.131.681.912.905	0.000110637	2.00E-10	plasminogen activator inhibitor 1 RNA-binding -like isoform X1	0
			c116967_g1	815	208.453.100.391.972	0.000918198	3,99E-131	14-3-3 epsilon	0
			c117041_g1	1640	298.017.701.384.834	3,67E+07	0.00E+00	vasa-like gene-2, partial	F:GO:0003676; F:GO:0005524; F:GO:0004386
			c117365_g1	569	23.863.719.943.952	7,69E+09	1.10E-28	H ACA ribonucleo complex subunit 3-like	P:GO:0001522; F:GO:0030515; P:GO:0042254
			c117384_g1	1970	213.643.559.966.073	0.004772888	1.40E-72	inositol 1,4,5-trisphosphate receptor type 3-like	0
			c117434_g1	717	247.753.511.880.476	5,15E+07	1,52E-39	beta-glucuronidase-like isoform X2	P:GO:0005975; F:GO:0004553
			c117673_g2	1216	439.492.333.638.209	4,95E+02	6,55E-97	glycine betaine transporter -like	0
			c117719_g1	556	237.313.505.404.236	0.00869003	2.40E-33	hypoxanthine-guanine phosphoribosyltransferase-like, partial	0
			c117796_g1	1743	151.934.382.895.155	0.007898611	0.0	NADP-dependent malic enzyme	P:GO:0006108; F:GO:0046872; F:GO:0051287; F:GO:0004471; P:GO:0055114
			c117801_g1	791	217.630.429.051.157	0.00818762	2,95E-27	glutathione S-transferase A-like	C:GO:0005737; P:GO:0006749; F:GO:0004364
			c117853_g1	213	210.754.998.261.244	0.003798546	6,20E-06	3-oxoacyl-[acyl-carrier-] reductase -like isoform X1	F:GO:0016491; P:GO:0055114
			c117914_g2	1178	205.626.258.999.001	0.001428578	5,43E-58	RWD domain-containing 1	0
			c118070_g1	263	33.764.434.423.408	0.001358009	2,47E-08	nucleoporin NUP53-like	F:GO:0000166; C:GO:0044615; C:GO:0031965; P:GO:0006999; P:GO:0055085; P:GO:0006607; P:GO:0006355; F:GO:0005543; F:GO:0005487; P:GO:0051028; C:GO:0044613; F:GO:0003697
			c118144_g1	641	431.772.966.776.446	1,34E+06	1,48E-23	fatty acid desaturase	C:GO:0016020
			c118219_g1	718	25.290.755.308.522	8,45E+09	1,27E-35	conserved regulator of innate immunity 3-like	C:GO:0005759
			c118259_g1	1856	400.943.860.341.497	1,11E+00	5,31E-90	transmembrane 201-like isoform X1	0
			c118450_g1	1897	348.763.504.487.445	1,89E+04	0.0	7-dehydrocholesterol reductase-like isoform X1	C:GO:0016020
			c118523_g2	2057	4.605.015.964.079	5,78E+05	2.90E-35	zinc transporter ZIP10-like	0
			c118581_g1	956	303.600.228.775.562	0.00256264	5.70E-84	enolase-phosphatase E1 isoform X2	0
			c118654_g1	220	345.055.022.515.416	4,63E+06	3.80E-35	arginine N-methyltransferase 1	F:GO:0008168; P:GO:0006479
			c118665_g1	594	243.769.597.970.997	0.000910108	4,02E-41	UPF0139 membrane C19orf56	C:GO:0016021
			c118776_g1	1116	21.770.329.161.324	0.001023371	1.50E-36	RNA-binding protein SmB	0
			c118879_g2	1315	273.710.271.341.571	3,28E+09	4,34E-49	inactive polypeptide N-acetylgalactosaminyltransferase 5	F:GO:0016757; C:GO:0016020; C:GO:0016021; P:GO:0006486; C:GO:0005794; F:GO:0016740; F:GO:0030246
			c118904_g1	569	487.494.530.387.722	4,11E+01	6,35E-40	SAM-dependent methyltransferase	F:GO:0008171; P:GO:0032259
			c118955_g1	1461	319.229.090.432.091	4,94E+04	5.20E-115	seleno J	0
			c119217_g2	876	441.114.717.890.598	7,66E+02	3,00E-58	methyltransferase-like protein 17, mitochondrial	0
			c119260_g1	855	38.268.619.951.768	5,04E+02	2,12E-25	glutathione S-transferase	C:GO:0005737; P:GO:0006749; F:GO:0004364
			c119303_g1	1506	309.195.707.673.486	0.000757491	1.70E-39	46 kDa FK506-binding nuclear -like	0
			c119329_g2	651	29.290.137.480.886	0.002174856	4.80E-48	H ACA ribonucleo complex subunit 1-like	0
			c119605_g1	928	208.127.905.927.142	8,80E+09	4,19E-07	basic proline-rich -like isoform X4	F:GO:0016757; F:GO:0016740
			c119632_g1	1973	373.654.021.502.648	4,13E+00	1.00E-06	glutenin, high molecular weight subunit PW212-like	0
			c119633_g1	520	213.686.220.841.534	0.000245624	4,70E-11	hyaluronan mediated motility receptor-like	F:GO:0005540
			c119643_g3	459	362.113.684.186.984	0.001706779	1.60E-64	ribosomal rpl27	0
			c119681_g1	321	235.433.009.277.484	3,66E+09	1,27E-24	cleft lip and palate transmembrane 1 homolog isoform X2	C:GO:0016020; C:GO:0016021
			c119744_g1	1375	44.021.467.746.545	1,26E+07	7.30E-24	PREDICTED: di-N-acetylchitobiase-like	0
			c119768_g1	1323	236.571.732.746.708	0.003029899	6,17E-88	insulin-like growth factor-binding -related 1	P:GO:0001558; C:GO:0005576; F:GO:0005520
			c119897_g2	323	443.159.897.713.292	0.000144991	6.70E-48	ribosomal L32	F:GO:0003735; C:GO:0005840; P:GO:0006412
			c119987_g1	854	17.592.603.263.899	0.00182336	1,40E-84	reticulon-1-A isoform X9	C:GO:0005789; C:GO:0016021
			c120006_g2	373	225.729.725.326.797	3,42E+09	1.80E-24	elongation factor 1-beta	F:GO:0003746; P:GO:0006414
			c120035_g2	359	226.685.440.268.941	0.000273275	2.10E-20	eukaryotic translation initiation factor 6	F:GO:0003743; F:GO:0043022; P:GO:0042256; P:GO:0006413
			c120093_g1	545	245.917.895.342.646	0.00295854	1.80E-09	dimethylaniline monooxygenase [N-oxide-forming] 4	0
			c120099_g1	214	246.548.516.918.609	0.000242321	1,57E-09	polymerase delta-interacting 2	F:GO:0003677
			c120105_g1	1144	277.553.657.914.184	0.001570463	1,70E-27	switch-associated 70-like	0
			c120164_g1	607	331.633.282.384.667	0.004523621	9,16E-59	serine threonine- kinase D3-like isoform X5	F:GO:0005524; F:GO:0046872; P:GO:0006468; C:GO:0005622; P:GO:0035556; F:GO:0004697
			c120224_g1	1597	293.077.510.392.933	7,71E+06	1.80E-14	Mid1-interacting ,	0
			c120255_g1	1776	480.090.579.428.324	2,17E+04	0.0	monocarboxylate transporter 13-like	P:GO:0055085; C:GO:0016021
			c120347_g2	620	298.701.199.814.426	6,76E+05	1.80E-86	3-hydroxyacyl- dehydrogenase type-2-like	0
			c120422_g1	1520	244.302.514.412.404	0.005763621	1.20E-37	N-terminal asparagine amidohydrolase	0
			c120445_g1	708	313.532.544.315.847	0.002588219	5.62E-22	Serine arginine-rich splicing factor	0
			c120528_g4	1847	284.268.005.672.878	0.000182205	9,92E-166	farnesyl pyrophosphate synthase-like	P:GO:0008299; F:GO:0016740
			c120624_g1	1090	276.104.139.234.041	0.008958234	2.10E-11	surfeit locus 6 homolog	0
			c120630_g1	575	200.538.303.260.908	0.002829048	1.60E-19	glucosidase 2 subunit beta	0
			c120786_g3	2091	244.112.188.404.937	0.000351494	9,70E-40	multiple PDZ domain -	0
			c120866_g1	1332	429.372.886.220.011	5,06E+08	3.90E-105	peroxiredoxin 6	P:GO:0098869; P:GO:0055114; F:GO:0051920
			c120880_g1	1196	212.389.400.051.613	0.002253429	4,11E-30	RNA-binding FUS-like isoform X2	0
			c120902_g1	2007	389.135.927.127.615	0.00032187	1,16E-64	unconventional prefoldin RPB5 interactor-like	0
			c120908_g3	1948	334.764.913.847.204	1,17E+08	7,47E-157	diphosphomevalonate decarboxylase-like	P:GO:0008299; F:GO:0016831
			c120986_g1	1967	37.559.498.805.089	1,65E+06	1,98E-38	collagen alpha-1(I) chain-like	F:GO:0005509; C:GO:0016020; C:GO:0016021; C:GO:0005578
			c120992_g2	876	193.615.927.168.029	0.001808575	7.30E-95	proteasome subunit alpha type-3 isoform X1	0
			c121004_g2	1661	329.965.137.561.651	2,47E+06	6.60E-06	glutenin, high molecular weight subunit PW212-like	0
			c121020_g1	1990	342.573.538.947.641	5,07E+09	2,13E-58	proton-coupled folate transporter-like	0
			c121067_g2	2248	326.471.491.771.234	5,85E+07	9,10E-59	Tumor necrosis alpha-induced 8	0
			c121111_g1	1064	164.434.744.749.895	0.002902996	3,64E-114	lysosomal protective -like isoform X2	F:GO:0004180
			c121146_g2	1204	161.654.685.787.447	0.00596723	5,97E-37	ATP synthase subunit mitochondrial-like	C:GO:0045261; F:GO:0046961; P:GO:0015986; F:GO:0046933
			c121150_g1	2259	160.766.542.107.557	0.009951048	3.30E-71	Translation machinery-associated 46	0
			c121161_g1	1313	235.728.458.598.803	1,32E+09	0.00E+00	beta-tubulin	0
			c121175_g1	473	345.713.671.474.079	0.004923277	6,26E-24	eukaryotic translation initiation factor 2 subunit 2	0
			c121309_g1	1871	220.000.774.311.323	0.000338265	1,66E-158	Serine threonine- phosphatase 6 regulatory ankyrin repeat subunit C	P:GO:0035556; C:GO:0005622
			c121360_g1	661	208.969.976.649.444	0.004816465	5.10E-62	60S ribosomal L13	0
			c121376_g1	479	314.689.518.465.662	6,37E+08	1.30E-07	Pulmonary surfactant-associated D	0
			c121435_g2	1394	331.932.588.340.258	9,56E+08	9.40E-131	transmembrane 147-like	0
			c121465_g1	1105	325.109.743.550.262	0.004303429	3,55E-07	tripartite motif-containing 52-like	F:GO:0008270; F:GO:0046872; C:GO:0005622
			c121482_g2	1636	256.374.327.173.608	0.00023874	0.0	serine--tRNA cytoplasmic-like	0
			c121575_g1	1109	150.907.503.430.924	0.008810356	6,48E-34	neurocalcin homolog [Orbicella faveolata]	0
			c121629_g1	1250	352.806.917.739.167	2,33E+08	4.20E-46	argininosuccinate lyase-like	0
			c121641_g3	2675	226.194.590.842.074	5,49E+09	3,34E-64	glycine receptor subunit alpha-3-like isoform X1	P:GO:0034220; C:GO:0016021; F:GO:0005230; C:GO:0005886
			c121683_g1	1929	354.777.647.000.389	6,89E+04	1,00E-47	glutathione S-transferase 1-like	0
			c121724_g1	1300	288.358.097.879.744	0.000101564	2,12E-103	aconitate mitochondrial	0
			c121743_g1	1863	333.451.296.835.617	2,04E+07	4,13E-79	transmembrane 246-like [Orbicella faveolata]	C:GO:0016020; C:GO:0016021
			c121807_g2	1926	224.651.573.833.985	0.001718058	2.90E-15	EMB-9, isoform b	0
			c121813_g2	1586	187.157.235.852.608	0.003650837	2.40E-103	Ubiquitin-conjugating enzyme E2-22 kDa	0
			c121873_g1	1986	265.289.633.127.566	0.000433329	0.00E+00	ribose-phosphate pyrophosphokinase 1-like isoform X1	0
			c121928_g1	1214	145.696.133.052.454	0.007607746	5,12E-135	inositol-3-phosphate synthase 1-A-like	0
			c121954_g2	368	281.861.375.862.985	0.000101361	6,04E-09	transcription initiation factor TFIID subunit 10-like	0
			c121958_g1	2135	328.824.704.384.528	0.002572875	1,29E-134	Prenylcysteine oxidase	0
			c121979_g1	280	272.486.964.436.012	0.003187753	1,96E-05	E3 ubiquitin- ligase TRIM33-like	F:GO:0008270; F:GO:0046872
			c122071_g1	631	526.853.577.318.513	5,77E+05	7.00E-24	1-phosphatidylinositol phosphodiesterase-like	0
			c122135_g1	1433	309.792.089.071.193	0.002902996	1.10E-145	Spermidine synthase	0
			c122150_g2	1683	161.238.401.498.021	0.007022832	7,10E-03	NADH dehydrogenase [ubiquinone] 1 beta subcomplex subunit mitochondrial	0
			c122157_g1	1846	352.323.951.371.363	9,08E+08	4.40E-163	CTNB_URECA ame: Full=Catenin beta ame: Full=Beta-catenin	C:GO:0005856; C:GO:0005737; P:GO:0007165; P:GO:0007155; P:GO:0034333; F:GO:0004871
			c122234_g1	763	212.549.927.311.832	0.001606257	3,80E-30	transmembrane 41B-like isoform X2	0
			c122237_g1	509	282.711.955.388.831	0.001638136	1,10E-27	bone morphogenetic 5-	F:GO:0008083; C:GO:0005576; P:GO:0040007
			c122265_g2	1353	382.211.828.478.585	3,35E+08	4.10E-28	Hyaluronan mediated motility receptor	0
			c122353_g3	1762	230.714.418.432.778	0.000709474	5,83E-170	mitochondrial 2-oxoglutarate malate carrier -like	C:GO:0016021; P:GO:0006810
			c122392_g1	1458	264.246.888.283.856	6,03E+08	1,90E-35	mitochondrial import inner membrane translocase subunit Tim13-like	F:GO:0046872; C:GO:0005739; P:GO:0015031
			c122438_g2	862	52.175.794.862.182	8,19E+07	2,19E-45	large neutral amino acids transporter small subunit 2 isoform X2	C:GO:0016020; C:GO:0016021; F:GO:0015171; P:GO:0003333
			c122447_g2	982	261.971.719.528.748	0.001309256	1,15E-123	cytosol aminopeptidase	0
			c122488_g1	1902	300.371.237.535.657	0.001010367	7.20E-50	axin , partial	0
			c122525_g1	2427	234.421.500.099.498	0.001872217	3,89E-45	phosphatase 1 regulatory subunit 3B-like isoform X2	0

STOLON FEMALE vs STOLON MALE

Upregulated in Female

c122561_g2	1346	238.168.482.258.341	0.001821378	2,30E-113	paramyosin isoform X3	0
c122562_g2	2233	329.524.296.630.575	0.002923145	3.40E-34	failed axon connections,	0
c122711_g1	792	316.330.518.357.713	0.005869874	7,05E-23	defender against apoptotic cell death 1	C:GO:0008250; F:GO:0004579; C:GO:0016021; P:GO:0006486
c122934_g1	510	201.494.124.810.477	0.007136015	1.90E-86	alcohol dehydrogenase class-3-like	0
c123035_g1	398	330.719.432.628.136	0.007978054	1.20E-15	cyclin-dependent kinase 6-like	0
c123221_g2	1463	573.693.692.822.029	1,11E+01	2.80E-151	G2 mitotic-specific cyclin-B	0
c123247_g1	2219	289.229.529.172.466	0.001515654	0.0	glutamate mitochondrial-like	0
c123295_g1	817	197.413.739.472.332	0.006781643	6.30E-32	glycine-rich cell wall structural -like isoform X3	0
c123307_g1	3754	351.959.570.740.927	6,87E+06	2.60E-64	collagen alpha-6(VI) chain-like isoform X1	0
c123312_g1	1908	215.266.885.367.833	4,84E+09	0.0	coiled-coil domain-containing 47-like	0
c123314_g1	1743	33.014.041.473.364	1,01E+08	3,23E-53	papilin-like isoform X1	F:GO:0008270; F:GO:0008061; P:GO:0006030; F:GO:0008233; C:GO:0005576; F:GO:0004222; C:GO:0005578; F:GO:0008237; F:GO:0030414; F:GO:0004867; P:GO:0006508; P:GO:0010951; C:GO:0031012; P:GO:0010466
c123355_g2	2174	246.406.233.588.539	0.004732939	1.50E-22	transcription factor Id	C:GO:0005634; P:GO:0045892; C:GO:0005737; F:GO:0046983
c123507_g1	2454	313.265.259.413.046	2,46E+08	8.20E-86	ubiquitin conjugating enzyme 2, isoform A	0
c123566_g1	2587	146.489.005.121.278	0.008466924	1.90E-180	COP9 signalosome complex subunit 6	0
c123601_g1	1038	20.177.603.262.607	8,12E+09	2,53E-15	myosin light chain smooth muscle-like isoform X3	0
c123646_g2	1382	232.488.748.243.544	0.000702827	3.50E-32	PREDICTED: nucleolin-like	0
c123662_g1	1416	308.984.629.433.975	1,45E+08	3.20E-14	zinc finger and SCAN domain-containing 2-like	0
c123673_g1	575	283.534.299.367.597	0.0013262	3,20E-16	4-trimethylaminobutyraldehyde dehydrogenase	F:GO:0016491; P:GO:0008152; P:GO:0055114; F:GO:0016620
c123675_g1	469	171.374.354.564.799	0.008256308	8,92E-72	40S ribosomal	F:GO:0003723; F:GO:0003735; C:GO:0005840; P:GO:0006412
c123684_g2	1232	273.370.752.478.313	0.000947799	9.40E-138	eukaryotic translation initiation factor 2A-like	0
c123833_g1	2315	211.544.002.089.274	0.007353564	1,06E-128	2-acylglycerol O-acyltransferase 1-like	0
c123834_g1	3182	159.514.958.117.849	0.005865627	1.20E-130	Mothers against decapentaplegic 4, partial	0
c123840_g1	2544	209.943.301.723.798	9,27E+09	6,74E-163	limb expression 1 homolog	0
c123850_g1	971	297.639.750.349.279	8,67E+08	1.10E-56	hydroxyacylglutathione hydrolase, mitochondrial-like isoform X3	0
c123905_g2	702	539.158.028.175.188	2,27E-03	1.10E-28	ribosomal L7Ae	0
c124061_g1	1705	353.654.949.837.668	0.00026355	1,57E-99	E3 ubiquitin- ligase RNF220-like isoform X1	0
c124279_g1	1076	298.613.429.421.482	0.001738481	8,05E-29	succinate dehydrogenase [ubiquinone] cytochrome b small mitochondrial isoform X2	C:GO:0016020; C:GO:0016021; C:GO:0005740
c124317_g2	869	236.353.320.259.822	0.00896138	3,54E-128	MOB phocein	0
c124338_g2	725	177.339.330.322.409	0.001093342	1,06E-97	mitochondrial manganese superoxide dismutase	F:GO:0046872; P:GO:0019430; P:GO:0055114; F:GO:0004784
c124402_g1	2586	147.101.499.928.588	0.007920413	0.0	Neuroligin- Y-linked	C:GO:0016020
c124497_g2	1596	200.179.840.272.785	0.000715874	1,41E-55	methyl transferase	F:GO:0008171; F:GO:0008168; F:GO:0016740; P:GO:0032259
c124562_g1	293	232.308.729.849.835	0.00918237	4,05E-19	nucleolar complex 2 homolog	0
c124569_g3	935	441.007.696.753.887	0.000464925	5.70E-39	thromboxane-A synthase	0
c124586_g1	1933	191.989.706.961.453	0.007889251	6,09E-97	stAR-related lipid transfer mitochondrial-like	F:GO:0008289
c124658_g1	2905	249.011.806.354.895	0.000255471	3.70E-138	F-box only 33	0
c124679_g1	1215	169.133.108.832.869	0.004911128	7,00E-89	valine--tRNA ligase-like	F:GO:0004832; F:GO:0005524; P:GO:0006438; C:GO:0005737; P:GO:0006450; F:GO:0002161
c124819_g2	2470	244.873.600.588.217	0.002642726	2,42E-110	leukocyte elastase inhibitor-like isoform X8	0
c124834_g1	2121	259.672.856.421.095	7,27E+09	0.0	twitchin isoform X1	0
c124885_g1	1871	237.322.885.295.907	0.000920187	1,30E-73	Carbonic anhydrase	0
c124899_g1	2301	203.630.216.670.626	0.001446702	0.00E+00	6-4 photolyase	0
c124911_g1	986	208.132.726.282.535	0.008186768	8,77E-80	28S ribosomal mitochondrial-like	C:GO:0005840
c124977_g1	2254	185.618.410.897.735	0.001260915	4,56E-115	39S ribosomal mitochondrial	F:GO:0003735; C:GO:0015934; P:GO:0006412
c125008_g1	2375	254.285.502.190.165	0.009094009	0.00E+00	Bifunctional ATP-dependent dihydroxyacetone kinase FAD-AMP lyase (cyclizing)	0
c125017_g3	410	269.259.182.187.088	1,64E+09	1.30E-08	S16	F:GO:0003735; C:GO:0030529; C:GO:0005840; P:GO:0006412
c125021_g1	3895	830.593.896.530.625	1,03E+00	1.10E-76	vitellogenin	P:GO:0006869; F:GO:0005319
c125025_g3	2493	290.230.398.225.149	2,44E+08	4.20E-20	two pore domain potassium channel number 2	0
c125086_g1	1340	185.478.534.586.517	0.001530053	2.60E-11	perilipin-2-like isoform X1	0
c125087_g1	1914	275.155.216.879.506	0.002947886	3.20E-85	PREDICTED: ik:tdsubc_1b12	0
c125131_g3	2176	203.700.123.583.494	0.001690972	2,97E-121	carnitine O-acetyltransferase-like isoform X2	F:GO:0016740
c125184_g1	1332	360.089.072.303.752	5,90E+08	5,19E-104	RTF2 homolog	C:GO:0005634; P:GO:1902979
c125242_g1	1999	208.744.664.544.504	0.000147241	1,56E-143	Solute carrier family 35 member C2	C:GO:0016021
c125256_g1	401	316.043.896.762.713	6,66E+06	3,34E-35	hydroxysteroid dehydrogenase 2 isoform X2	0
c125332_g1	482	345.223.017.119.209	2,90E+07	1.60E-46	methionine synthase-like	0
c125340_g1	347	333.239.809.732.647	0.00024058	6.20E-30	acyl-coenzyme A synthetase ACSM3	0
c125378_g1	1817	307.904.670.456.546	8,35E+05	1,70E-22	Flagellar hook-length control	0
c125452_g1	1386	253.137.098.317.278	0.007406674	3.40E-86	39S ribosomal L44, mitochondrial-like	0
c125473_g1	1488	333.618.284.264.087	2,03E+06	2,65E-168	alpha-L-fucosidase-like isoform X3	F:GO:0004560; P:GO:0006004
c125615_g2	1658	230.201.357.539.056	8,59E+09	2,79E-09	PAR domain subfamily bZIP	F:GO:0003700; P:GO:0006355; F:GO:0043565
c125738_g3	303	424.299.939.824.509	2,16E+06	2.20E-16	ATP-dependent DNA helicase PIF1	0
c125741_g2	2839	222.915.217.756.132	0.002124646	0.0	serine threonine- kinase PRP4 homolog	F:GO:0005524; F:GO:0004672; P:GO:0006468
c125892_g1	2798	335.038.928.653.203	2,44E+08	7.50E-08	methyl- -binding 2 isoform X1	0
c125958_g1	1871	33.540.290.282.045	0.007525163	0.0	farnesyltransferase subunit beta-like	C:GO:0005965; F:GO:0003824; P:GO:0018343; P:GO:0042127
c126003_g1	1458	343.969.010.876.238	0.008431044	1.40E-57	PREDICTED: beta-mannosidase-like	0
c126064_g1	1228	250.332.315.954.144	0.005619778	5.20E-19	transcription factor A, mitochondrial	0
c126070_g1	1163	201.603.226.340.319	0.000396286	8,63E-86	oncosphere Tso22e	P:GO:0006937; C:GO:0005861
c126090_g1	965	391.398.127.810.574	0.000333155	3,05E-28	kinesin KIF14 isoform X1	0
c126129_g1	1599	403.649.899.457.844	0.000130511	7.10E-66	UPF0587 v1g245604-like	0
c126180_g2	1384	236.481.299.788.662	0.006185369	3,49E-84	nuclear pore complex Nup88-like	0
c126228_g1	820	206.640.678.350.058	0.000203661	6,42E-28	elongation factor mitochondrial-like	F:GO:0000166
c126246_g1	3616	160.577.598.237.518	0.004576372	0.00E+00	Serine threonine kinase NLK	0
c126269_g2	2255	264.748.943.506.995	6,16E+07	0.0	nucleolar RNA helicase 2-like	0
c126275_g2	340	381.291.147.877.408	0.000465746	2.20E-10	Agmatinase, mitochondrial	0
c126323_g1	2056	161.241.232.520.068	0.004768619	5,95E-47	clathrin light chain A-like isoform X6	0
c126326_g1	2102	158.334.774.051.151	0.005997799	1.20E-147	F-actin-capping subunit alpha	0
c126371_g1	839	279.387.307.481.324	0.000953453	1,72E-56	probable UDP-sugar transporter SLC35A5	0
c126377_g1	2316	311.996.651.018.005	0.002996307	1.10E-178	ATP-dependent RNA helicase DDX51	0
c126389_g1	2589	290.944.461.724.163	0.007513094	1.60E-37	Usher syndrome type-1G homolog	0
c126393_g1	432	286.962.066.167.489	8,11E+06	1.00E-26	transmembrane 65	0
c126404_g2	943	186.847.545.259.873	0.007513094	1.30E-72	ubiquitin-conjugating enzyme E2-17 kDa-like	0
c126425_g2	724	28.216.487.127.006	2,97E+08	3,34E-142	isocitrate dehydrogenase [NADP] cytoplasmic-like	F:GO:0000287; F:GO:0051287; F:GO:0004450; P:GO:0006102; P:GO:0006099
c126428_g1	1445	28.866.706.992.043	0.004233234	1,53E-46	beta-1,4-N-acetylgalactosaminyltransferase bre-4-like	P:GO:0005975; F:GO:0016757; C:GO:0016020; C:GO:0016021
c126460_g1	549	250.453.192.563.807	4,41E+09	6.20E-27	Cell division cycle 5-related	0
c126490_g1	2799	369.154.169.494.962	1,31E+05	3.40E-130	Ankyrin repeat domain-containing 50	0
c126506_g3	1084	295.546.948.397.991	2,13E+07	1,85E-42	mitochondrial import inner membrane translocase subunit TIM14-like isoform X2	0
c126542_g1	2511	438.982.130.292.818	7,27E+06	1,91E-98	carbonic anhydrase	0
c126573_g2	932	369.132.685.986.234	0.000405369	2.60E-15	methyltransferase 21D	0
c126592_g2	1696	165.601.751.837.999	0.009224699	0.0	4-hydroxybutyrate coenzyme A transferase-like	F:GO:0003824; P:GO:0006084
c126653_g1	1251	318.846.259.727.947	0.00241773	3,69E-38	MAD2L1-binding -like	0
c126675_g1	1445	20.093.935.872.196	0.001793974	1,00E-31	elongation of very long chain fatty acids 2-like isoform X3	C:GO:0016020; P:GO:0006631
c126738_g1	663	215.770.311.763.561	0.00495662	8.00E-16	OTU domain-containing 6B	0
c126771_g1	2697	259.487.067.689.149	5,24E+09	1,86E-147	hexokinase-2-like isoform X3	P:GO:0005975; F:GO:0036094; F:GO:0016772; P:GO:0016310
c126816_g1	1481	256.440.806.164.241	1,99E+09	5,08E-95	poly(U)-specific endoribonuclease-A	F:GO:0016788
c126905_g1	1344	280.214.455.847.267	0.003513234	3.50E-70	Adenosine kinase 1	0
c126927_g3	2820	216.859.770.363.865	0.001609843	8.60E-96	Exosome complex exonuclease RRP41	0
c126981_g1	2131	276.273.367.971.345	4,00E+09	2,17E-146	beta-1,3-galactosyltransferase 1-like	C:GO:0016021; P:GO:0006486; F:GO:0008378; C:GO:0000139
c126998_g1	1494	31.083.768.118.242	6,56E+05	1.10E-157	kinase DC2	0
c127092_g1	3247	189.979.897.276.732	0.000347293	5.80E-168	WD repeat-containing 75-like	0
c127132_g1	1455	381.254.806.163.322	2,41E+07	0.0	60 kDa heat shock mitochondrial-like	F:GO:0005524; C:GO:0005737; P:GO:0042026
c127169_g2	492	399.810.161.475.606	4,94E+07	8,37E-50	Fatty acid desaturase 2	F:GO:0016491; P:GO:0006629; C:GO:0016020; C:GO:0016021; P:GO:0055114
c127173_g1	2651	199.253.941.791.848	0.001947034	0.0	V-type proton ATPase subunit d 1	0
c127178_g1	2003	427.350.489.419.367	9,34E+09	4,24E-55	moesin ezrin radixin homolog 1	0
c127210_g1	812	303.668.233.417.267	0.000304948	1,63E-66	DNA-directed RNA polymerase III subunit RPC5-like	C:GO:0005634; F:GO:0003899; P:GO:0006351
c127285_g2	2400	543.126.951.185.336	7,98E-06	2,64E-16	SH3 and PX domain-containing 2B	0
c127289_g3	415	268.905.792.692.341	0.002178131	2,32E-10	deleted in malignant brain tumors 1 -like isoform X8	0
c127365_g1	2038	434.491.019.049.428	9,14E+05	1.10E-42	carbohydrate sulfotransferase,	0
c127471_g1	1197	365.005.829.305.863	0.00194288	5.20E-69	omega class glutathione-s-transferase	0
c127478_g2	1017	309.116.169.850.425	6,11E+09	1,01E-33	histone-binding N1 N2-like isoform X2	0
c127500_g1	2053	299.944.983.118.324	0.007203831	0.00E+00	Rh-related protein	0
c127514_g1	1059	289.751.459.415.905	0.008335043	6.70E-99	cytochrome c oxidase assembly COX11, mitochondrial-like	0
c127566_g1	816	504.449.817.238.472	1,95E+05	1.40E-63	cyclin A	0
c127570_g1	339	191.719.919.058.585	0.00217632	7.50E-10	JC7958galaxin precursor - reef coral	0
c127588_g1	1939	208.687.388.281.488	0.007920413	5,48E-22	probable serine threonine- kinase kinX isoform X2	0
c127658_g1	2517	317.688.749.794.461	0.000511048	8,50E-71	transcription factor kayak-like	0
c127707_g2	4028	728.731.703.894.305	1,13E+04	1.80E-25	vitellogenin	0
c127742_g1	3001	151.322.542.862.277	0.005193168	3.60E-29	kunitz-type protease inhibitor 2-like isoform 2	0
c127794_g1	888	226.897.396.411.103	0.000495128	4,71E-65	PREDICTED: importin-11-like	0
c127801_g1	2011	194.907.594.679.766	0.001659945	1,86E-37	chitin synthase	C:GO:0016459; F:GO:0005524; F:GO:0016758; C:GO:0016020; C:GO:0016021; F:GO:0003774
c127811_g1	1802	247.543.057.883.585	0.00338867	6.20E-86	translocon-associated subunit alpha-like	0
c127827_g1	657	371.038.511.536.951	0.00213386	2.30E-23	synaptic vesicle 2-related -like, partial	0
c127877_g1	1967	220.528.871.828.256	0.000226688	1,25E-148	heparan sulfate glucosamine 3-O-sulfotransferase 3B1-like	C:GO:0016021; F:GO:0008146
c127935_g2	870	146.474.838.645.828	0.007406674	2.30E-180	chaperonin containing T-complex polypeptide subunit zeta	0
c127961_g1	1771	282.424.135.495.059	0.00034695	1.90E-22	heat shock 60	F:GO:0005524; C:GO:0005737; P:GO:0042026
c127996_g1	1152	39.198.559.955.391	2,19E+08	3.60E-33	arginine kinase	F:GO:0000166; F:GO:0016772
c128008_g1	533	190.667.332.497.247	0.003691513	2.20E-36	Serine threonine- phosphatase 4 regulatory subunit 2-A	0
c128019_g1	2121	315.693.650.285.492	5,07E+09	5,17E-33	methyltransferase DDB_G0268948 isoform X2	0
c128074_g3	2339	221.982.817.316.473	0.003000289	0.00E+00	actin-related 2 isoform X2	F:GO:0005524; P:GO:0034314; C:GO:0005885
c128114_g1	1657	235.324.066.545.202	0.007893289	0.00E+00	methylmalonate-semialdehyde dehydrogenase [acylating], mitochondrial	0
c128117_g1	1316	339.327.442.470.642	1,20E+09	1.90E-40	fem-1 C	0
c128117_g2	1712	300.041.355.862.037	5,82E+09	0.00E+00	fem-1 C	0
c128130_g1	2248	259.539.728.121.988	0.002114984	0.0	T-complex 1 subunit epsilon-like	F:GO:0005524; C:GO:0005737; F:GO:0051082; P:GO:0006457
c128146_g1	1788	354.197.423.230.619	0.001608852	1.30E-22	mpv17 -like isoform X1	0
c128239_g1	3864	174.599.080.053.433	0.005077296	3,46E-19	SERTA domain-containing 4	0
c128270_g1	2427	310.546.144.321.967	7,69E+09	3,10E-66	patatin-like phospholipase domain-containing 4 isoform X1	P:GO:0008152; P:GO:0006629
c128287_g4	1133	264.586.874.843.499	0.005563588	5.80E-80	nucleoside diphosphate kinase-like	0
c128323_g1	1835	313.639.131.996.434	1,35E+02	0.00E+00	homologue of Sarcophaga 26,29kDa ase	0
c128416_g1	2165	252.249.549.191.224	0.001867137	4.20E-172	PEPM_MYTED ame: Full=Phosphoenolpyruvate phosphomutase Short=PEP mutase Short=PEP phosphomutase Short=Phosphoenolpyruvate mutase	0
c128452_g4	1123	275.344.926.772.938	0.000867763	1,56E-53	heat shock 75 mitochondrial-like	F:GO:0005524; F:GO:0051082; P:GO:0006457; P:GO:0006950
c128466_g1	3016	25.429.929.230.601	1,51E+09	1.30E-167	Vacuolar 8	0
c128493_g1	1040	280.127.910.192.045	0.000501925	2.00E-115	RRM domain Musashi	F:GO:0000166; F:GO:0003676
c128509_g1	1228	21.796.654.807.834	0.007387901	0.00E+00	uroporphyrinogen decarboxylase isoform X10	0
c128544_g1	1394	191.468.487.093.767	0.000189709	3,68E-01	nidogen-1-like isoform X1	P:GO:0007160; F:GO:0005509
c128567_g1	706	310.504.115.043.498	4,27E+06	5,58E-24	glycosylated lysosomal membrane A-like	C:GO:0016020; C:GO:0016021
c128590_g1	673	372.722.304.839.262	0.000425483	1,01E-39	papilin isoform X4	F:GO:0008270; F:GO:0008061; P:GO:0006030; F:GO:0008233; C:GO:0005576; F:GO:0004222; C:GO:0005578; F:GO:0008237; F:GO:0030414; F:GO:0004867; P:GO:0006508; P:GO:0010951; C:GO:0031012; P:GO:0010466
c128590_g2	1233	433.433.681.288.018	4,60E+09	7.10E-83	papilin-like, partial	0
c128684_g1	2177	18.414.549.074.932	0.000474837	0.0	eukaryotic translation initiation factor 3 subunit L-like	0
c128716_g1	411	164.181.868.990.633	0.003366895	6.80E-20	ribosomal L29	F:GO:0003735; C:GO:0005840; P:GO:0006412
c128733_g2	1339	353.076.973.980.758	1,69E+06	6.40E-20	PREDICTED: protein scylla-like	P:GO:0009968; C:GO:0005737
c128877_g1	432	18.020.449.727.653	0.00925025	7.70E-50	staphylococcal nuclease domain-containing 1-like	0
c128884_g1	2580	289.942.883.693.377	0.006636476	7.10E-74	Tyrosine- phosphatase non-receptor type 6	0
c128890_g1	781	295.483.269.636.698	6,07E+08	5.30E-25	granulins-like, partial	0
c128902_g1	2527	375.703.971.007.937	0.000347293	8.40E-16	KLHL8_CAEEL ame: Full=Kelch 8	0
c128919_g1	413	271.027.671.340.795	0.003344121	5.80E-58	probable serine carboxypeptidase CPVL isoform X3	0
c128919_g2	982	244.016.295.611.217	0.00665126	8.70E-110	serine carboxypeptidase CPVL	0
c128928_g1	1872	241.128.150.105.034	0.002086198	4.00E-166	paramyosin-3, partial	0
c129085_g3	1761	181.195.777.445.573	0.001543799	5,95E-130	leupaxin-like isoform X2	P:GO:0007160; F:GO:0008270; C:GO:0005856
c129090_g1	626	166.742.909.544.702	0.002454564	6,31E-38	E3 ubiquitin- ligase UHRF1-like	0
c129100_g1	1921	202.479.958.167.286	0.000138509	0.00E+00	aminopeptidase -like	P:GO:0019538; C:GO:0005622; F:GO:0008238
c129109_g1	2677	343.484.482.162.564	7,69E+02	3.10E-06	collagen alpha-1(XVII) chain-like	0
c129124_g1	3036	198.102.519.703.111	0.000954722	8.80E-37	Insulin-like growth factor-binding complex acid labile chain	0
c129140_g1	3188	172.170.131.650.162	0.002642726	2,01E-32	E3 ubiquitin- ligase Mdm2-like isoform X3	P:GO:0000122; F:GO:0008270; F:GO:0046872; P:GO:0016567
c129141_g1	3679	341.268.286.956.961	0.001765139	8.10E-111	relaxin receptor 1 isoform X2	0
c129152_g1	1514	194.008.001.099.945	0.002572875	2,29E-47	double zinc ribbon and ankyrin repeat-containing 1-like	0
c129167_g4	278	238.883.191.063.226	6,46E+09	3,16E-13	asparagine synthetase [glutamine-hydrolyzing]-like	0
c129248_g4	2432	187.177.585.984.739	0.0025895	4,93E-152	eukaryotic translation initiation factor 3 subunit H-like	0
c129308_g2	1798	282.836.869.687.119	4,60E+09	6,55E-22	bcl-2 homologous antagonist killer-like	P:GO:0043065
c129334_g1	3585	313.046.349.774.182	0.002322975	0.0	importin-9-like isoform X1	0
c129351_g1	3621	289.423.112.057.785	0.000287668	4,30E-94	dentin sialophospho -like	0
c129383_g1	1752	215.368.429.688.069	0.000477577	2,76E-45	Group 3 secretory phospholipase	0
c129385_g1	767	221.564.248.929.902	0.00896138	5.00E-20	src-family tyrosine kinase	P:GO:0016310; F:GO:0016301
c129441_g2	1712	209.944.222.653.484	0.008527224	1,09E-70	dnaJ homolog subfamily B member 11-like	F:GO:0051082; P:GO:0006457
c129471_g1	3446	378.126.858.726.186	2,16E+03	2,39E-171	frizzled1 2 7	F:GO:0004888; C:GO:0016020; P:GO:0016055
c129503_g1	1176	231.990.799.091.717	0.001811517	1.70E-08	polyadenylate-binding 2 isoform X2	0
c129580_g1	244	36.675.226.954.554	0.000596765	8.90E-07	Carbonic anhydrase 13	0
c129586_g1	2705	641.355.097.048.756	8,14E+04	3.20E-10	transmembrane protease serine 2-like	0
c129596_g3	992	230.480.636.784.584	0.003819957	8.50E-61	PREDICTED: prohibitin-like	C:GO:0016020
c129642_g2	1392	193.772.744.075.232	0.008085614	0.00E+00	catalase	P:GO:0006979; F:GO:0046872; F:GO:0004096; P:GO:0098869; P:GO:0055114; F:GO:0020037; P:GO:0042744
c129643_g1	828	518.874.558.980.675	9,49E+05	8,63E-29	Multidrug-efflux transporter	P:GO:0006855; F:GO:0015297; C:GO:0016021; F:GO:0015238
c129692_g1	989	286.514.233.712.429	0.000369437	7,79E-03	probable G- coupled receptor 139	F:GO:0004930; P:GO:0007186; C:GO:0016020; C:GO:0016021
c129694_g2	1023	355.424.245.834.206	0.000743918	9,34E-87	transcription elongation factor B polypeptide 3-like	0
c129795_g1	2063	18.536.371.187.799	0.00318004	1.70E-64	Protein dodo	F:GO:0003755; P:GO:0000413
c129809_g1	3193	164.985.305.864.233	0.008110352	3,84E-155	phosphatidylinositol 3-kinase regulatory subunit gamma-like isoform X2	0
c129822_g1	1351	368.120.156.918.922	1,25E+09	3,19E-07	eva-1 homolog C isoform X1	0
c129906_g1	1576	379.228.766.875.287	0.000240168	1,62E-57	High-affinity choline transport	0
c129965_g1	4247	453.381.125.743.225	1,59E-04	0.0	papilin-like isoform X14	F:GO:0008270; F:GO:0008061; P:GO:0006030; F:GO:0008233; C:GO:0005576; F:GO:0004222; C:GO:0005578; F:GO:0008237; F:GO:0030414; F:GO:0004867; P:GO:0006508; P:GO:0010951; C:GO:0031012; P:GO:0010466
c129977_g2	2335	291.400.044.944.314	0.000229756	8,56E-138	DUF2961 domain-containing	0
c130027_g1	2988	197.426.133.320.687	0.001481361	1,72E-34	EF-hand calcium-binding domain-containing 6	0
c130074_g1	717	243.833.810.466.372	0.000171866	2,09E-80	3-hydroxy-3-methylglutaryl-coenzyme A reductase-like	C:GO:0005789; F:GO:0050661; C:GO:0016021; P:GO:0015936; P:GO:0055114; P:GO:0008299; F:GO:0004420
c130092_g1	1846	357.159.536.291.107	7,52E+07	0.00E+00	TGF beta signaling pathway factor SMAD5	0
c130097_g1	215	312.754.424.585.486	0.002251712	5.70E-22	DEAD box ATP dependent RNA helicase, partial	F:GO:0003676; F:GO:0005524; F:GO:0004386
c130118_g3	587	197.816.126.374.095	0.005563588	3.40E-75	ribosomal rps20	F:GO:0003723; F:GO:0003735; C:GO:0015935; P:GO:0006412
c130136_g2	1752	317.295.594.874.375	0.007167145	2.90E-06	GH3 domain-containing isoform X1	0
c130188_g2	1252	380.830.719.358.693	1,63E+06	3.40E-80	PREDICTED: neurotactin-like	0
c130293_g1	726	202.591.534.591.068	0.005748206	1,17E-62	glycerol-3-phosphate acyltransferase mitochondrial-	0
c130373_g2	1745	316.948.234.356.169	0.000914339	8.20E-17	malignant T-cell-amplified sequence 1-B-like	0
c130404_g1	397	402.473.418.291.582	3,27E+06	1,49E-29	PREDICTED: cyclin-J-like	0
c130413_g1	2053	192.096.242.044.116	0.00999295	1,84E-172	succinate-- ligase [ADP GDP-forming] subunit mitochondrial-like isoform X2	0
c130447_g1	664	272.021.307.068.255	0.00099891	1.30E-44	Mastl , partial	0
c130537_g1	4675	175.058.557.892.867	0.001171193	7.20E-60	Forkhead box N4	0
c130619_g1	3274	217.016.446.564.827	0.008810356	1.50E-52	nuclear receptor coactivator 5-like	0
c130622_g1	2018	212.887.644.328.521	0.001769927	0.00E+00	cytosolic nonspecific dipeptidase	0
c130630_g1	1920	249.250.063.140.979	0.003836731	0.00E+00	Aldehyde dehydrogenase, mitochondrial	0
c130701_g1	579	290.309.107.721.543	1,69E+07	5,04E-30	nicotinamide mononucleotide adenylyltransferase 1-like	F:GO:0005524; F:GO:0004515; P:GO:0034628; F:GO:0000309; C:GO:0005739
c130709_g1	706	164.333.400.598.753	0.007889251	1.10E-76	DNA-directed RNA polymerase II subunit RPB2	0
c130711_g1	1072	357.204.799.337.166	8,19E+07	1.20E-24	glutaredoxin	0
c130717_g2	2029	269.844.429.518.525	0.008308837	6.90E-176	chondroitin sulfate synthase 1	0
c130720_g1	1849	299.693.454.410.256	4,43E+06	6,10E-165	homogentisate 1,2-dioxygenase	P:GO:0006559; P:GO:0006570; P:GO:0055114; F:GO:0004411
c130730_g2	1852	210.806.845.290.236	5,52E+09	0.00E+00	apoptosis inhibitor 5	0
c130752_g1	3013	302.524.173.347.475	6,97E+08	2.70E-108	DNA replication factor Cdt1	F:GO:0003677; C:GO:0005634; P:GO:0000076; P:GO:0030174; P:GO:0033262
c130758_g3	399	257.912.977.135.194	0.001769225	2.90E-46	myosin essential light chain	F:GO:0005509
c130774_g2	2035	154.650.186.829.903	0.00355871	1,59E-25	C-myc promoter-binding -like	0
c130834_g1	2616	253.829.589.347.484	6,56E+08	0.0	acyl-coenzyme A peroxisomal-like isoform X2	0
c130868_g2	3245	265.662.561.952.116	0.000715874	1.60E-13	Nuclear respiratory factor 1	0
c130879_g1	2234	265.315.104.929.884	3,43E+09	0.00E+00	nicalin-1-like isoform X1	0
c130930_g1	1659	271.061.048.667.109	2,56E+07	0.0	glycine betaine transporter -like	0
c130939_g1	2374	191.835.863.464.756	0.000205365	0.0	succinyl- ligase [GDP-forming] subunit mitochondrial-like	0
c130948_g1	2176	290.871.796.192.173	0.002873395	4.00E-20	serine threonine- kinase 40, partial	0
c130963_g1	3602	164.172.632.953.628	0.005897654	3.60E-11	SERTA domain-containing 2 isoform X1	0
c130968_g1	2605	16.678.431.372.605	0.003742213	0.0	ral guanine nucleotide dissociation stimulator-like 1	P:GO:0007165
c131036_g1	852	21.591.208.424.978	0.000747789	9.20E-55	ribosomal L8e	F:GO:0003735; C:GO:0005840; P:GO:0006412
c131040_g2	1399	228.415.103.795.883	2,65E+09	1,50E-112	scavenger receptor class B type I	C:GO:0016020; C:GO:0016021
c131090_g1	2272	346.739.990.058.035	0.001537214	1,76E-83	somatomedin-B and thrombospondin type-1 domain-containing -like	P:GO:0006955; F:GO:0005044; P:GO:0006898; F:GO:0030247
c131093_g2	525	274.165.036.107.253	0.003414939	7.40E-33	pre-rRNA-processing TSR1 homolog isoform X1	C:GO:0005634; P:GO:0042254
c131094_g2	386	278.188.149.292.513	0.00032187	7.20E-44	AGAP003428-PA	0
c131138_g1	1454	218.786.184.400.269	0.007920413	2,17E-15	calpain-B-like isoform X4	F:GO:0008234
c131141_g2	1982	195.211.365.812.258	0.000236013	3.60E-129	casein kinase II subunit beta-like	C:GO:0005956; P:GO:0045859; F:GO:0019887
c131153_g2	621	439.559.498.908.791	0.000131891	5.10E-11	zinc finger 68 isoform 1	0
c131201_g1	4825	166.829.239.352.138	0.005629127	0.0	retinoblastoma 1	0
c131222_g2	4246	194.607.066.765.717	0.000657363	4.30E-129	cGMP-specific 3 ,5 -cyclic phosphodiesterase-like	0
c131251_g1	2481	298.463.317.456.618	0.00141625	0.00E+00	glutaminase kidney isoform, mitochondrial	0

c131278_g1	374	233.129.444.736.831	0.002086198	3,41E-15	probable ATP-dependent RNA helicase DHX37	0
c131334_g1	4006	238.449.435.976.145	5,12E+08	1.20E-177	ATPase WRNIP1-like	0
c131576_g1	1412	295.941.468.880.363	0.000114941	2.30E-57	fatty acid desaturase 1-like isoform X1	0
c131582_g1	3500	244.295.885.626.149	0.000790805	8,06E-140	proteasome subunit beta type-5-like	0
c131587_g1	240	178.326.126.903.462	0.008256308	1.90E-36	U65476_1phosphoglucose isomerase	F:GO:0004347; P:GO:0006094; P:GO:0006096
c131617_g1	2060	253.149.137.379.608	0.000152138	0.0	long-chain-fatty-acid-- ligase ACSBG2-like isoform X2	F:GO:0003824; P:GO:0008152
c131622_g1	2578	401.835.565.932.342	0.000271124	1,39E-46	dolichol kinase-like	0
c131681_g1	2962	195.301.047.104.319	0.00940442	0.0	dolichyl-diphosphooligosaccharide-- glycosyltransferase subunit STT3A	0
c131682_g1	1052	211.772.005.312.157	0.002762017	3.00E-94	methyltransferase 13 isoform X2	0
c131737_g1	255	21.189.880.583.792	0.000119807	4,84E-14	neuroendocrine convertase 2-like	0
c131741_g1	3188	264.828.939.846.355	0.003947984	6,20E-113	quinone oxidoreductase 1	0
c131795_g1	687	167.292.129.232.367	0.002599536	2,35E-08	calcium-dependent kinase SK5-like	F:GO:0005509
c131825_g1	3346	631.578.508.155.476	7,69E-06	1.70E-92	Tolloid 2	0
c131859_g1	3178	244.890.366.783.752	0.002897611	0.0	ATP-binding cassette sub-family B member mitochondrial	0
c131861_g1	2525	297.850.487.310.216	0.003028675	1.00E-122	Peptide-N(4)-(N-acetyl-beta-glucosaminyl)asparagine amidase	P:GO:0006516; C:GO:0005737
c131879_g1	2691	228.037.952.029.217	0.000457196	3,13E-48	Ubiquitin family	C:GO:0016020
c131892_g1	346	643.653.276.161.302	6,23E-13	5,95E-21	H ACA ribonucleo complex non-core subunit NAF1-like	0
c131896_g1	282	165.188.083.690.644	0.002534852	4,24E-21	limb region 1 -like	0
c131920_g1	3952	306.427.167.506.646	6,01E+09	9.70E-13	glutenin, high molecular weight subunit PW212-like	0
c131923_g2	1920	262.900.421.189.358	9,04E+09	3,66E-26	LIM domain-binding 3-like isoform X3	0
c131944_g1	2827	408.769.197.592.509	4,78E+05	0.0	sodium- and chloride-dependent neutral and basic amino acid transporter B(0+)-like	C:GO:0016020; F:GO:0015293; P:GO:0006810
c131969_g1	890	281.906.557.415.229	2,49E+08	4.60E-26	insulin-like growth factor-binding complex acid labile subunit-like	0
c132038_g1	2395	240.854.346.577.801	7,09E+08	3,20E-72	tethering factor for nuclear proteasome sts1-like isoform X1	0
c132088_g2	3675	171.101.968.957.347	0.007964079	2.50E-14	Collagen triple helix repeat and Type 4 procollagen domain containing	C:GO:0005581
c132119_g2	2038	331.569.458.765.243	2,10E+05	0.0	phosphoribosylformylglycinamidine synthase-like	0
c132129_g1	3520	288.802.566.432.365	6,19E+09	0.0	origin recognition complex subunit 1-like	0
c132148_g1	3619	377.716.742.726.357	5,85E+06	5.10E-12	fibrous sheath CABYR-binding -like isoform X1	0
c132158_g1	498	243.170.882.650.234	4,60E+09	2.20E-49	interferon-related developmental regulator 2-like	0
c132196_g2	2481	276.680.554.932.159	0.00226162	0.0	Sodium- and chloride-dependent glycine transporter 2	0
c132214_g1	1824	213.063.003.787.128	0.006366629	0.0	UDP-glucose 6-dehydrogenase-like isoform X2	F:GO:0051287; F:GO:0003979; P:GO:0055114
c132215_g1	4149	241.503.816.329.949	0.000747481	3.10E-42	midnolin-B-like isoform X2	0
c132249_g3	455	213.597.930.370.811	0.00070366	1.90E-18	ERI1 exoribonuclease 3-like	0
c132261_g1	1854	234.065.327.561.202	6,34E+09	0.00E+00	signal recognition particle subunit SRP68-like	0
c132294_g2	1877	17.566.597.173.181	0.003006073	4,85E-148	rac GTPase-activating 1-like	0
c132322_g4	860	276.281.299.036.268	0.001567552	1,53E-94	annexin A13	0
c132342_g1	3021	176.469.807.748.677	0.0025895	4,05E-89	platelet-activating factor acetylhydrolase IB subunit gamma-like	0
c132345_g1	2901	225.592.933.579.467	0.003038291	1,20E-60	ceramide synthase 1-like	F:GO:0050291; P:GO:0046513; C:GO:0005783
c132346_g1	276	259.533.882.826.091	4,73E+09	8.00E-21	glutamyl-tRNA(Gln) amidotransferase subunit B, mitochondrial	0
c132363_g3	502	234.033.081.186.448	0.005897654	6.10E-07	Nucleolin	0
c132413_g1	2580	167.685.958.109.286	0.007353564	0.0	inositol 1,4,5-trisphosphate receptor type 1-like	0
c132414_g2	1024	318.378.678.474.688	1,17E+08	1.90E-19	RNA-binding 26	0
c132414_g3	1111	365.596.052.958.125	4,52E+07	2,31E-20	RNA-binding 26-like isoform X2	F:GO:0005515; P:GO:0010923; F:GO:0044822
c132437_g1	3111	285.149.584.543.488	0.006804336	4.90E-11	GG18972	0
c132461_g1	2933	356.689.954.160.142	2,85E+05	9.10E-92	egl nine 1	0
c132465_g1	2780	319.489.800.536.621	0.001341986	5,44E-30	galactosylceramide sulfotransferase-like	0
c132477_g1	2462	186.145.054.739.151	0.005439984	0.00E+00	Lodestar protein	0
c132515_g1	627	206.764.737.765.274	6,02E+09	1,19E-38	peroxisome assembly 26-like	0
c132518_g1	400	167.351.681.192.714	0.0080232	3,11E-23	helicase domino-like isoform X5	F:GO:0005524
c132567_g1	224	216.947.661.788.215	0.001162011	3.80E-14	ABC transporter, ATP-binding	0
c132591_g1	3288	190.735.623.765.407	0.004470543	0.0	RRP12 isoform X2	0
c132600_g2	2041	202.070.397.513.006	0.00159481	1.30E-81	UMP-CMP kinase	C:GO:0005634; F:GO:0004127; F:GO:0005524; F:GO:0009041; P:GO:0006207; C:GO:0005737; P:GO:0009142; P:GO:0006165; F:GO:0004550; P:GO:0006221
c132606_g2	1514	156.421.780.355.938	0.005353612	1,93E-103	proteasome subunit beta type-1-B-like	C:GO:0005634; C:GO:0005737; C:GO:0005839; F:GO:0004298; P:GO:0051603
c132614_g1	5334	299.644.001.823.904	0.005004005	0.00E+00	myosin heavy chain isoform A	F:GO:0000166; F:GO:0043168
c132621_g1	365	273.876.764.575.063	1,28E+07	5.30E-10	Cyclin-O	0
c132633_g2	1735	448.056.081.801.923	4,14E+07	7,04E-148	kelch 18 isoform X3	0
c132681_g2	397	268.989.058.435.729	1,14E+09	3.40E-35	Bifunctional polynucleotide phosphatase kinase	0
c132693_g1	911	199.622.553.834.366	0.00169141	5.20E-37	PREDICTED: ubinuclein-2-like	0
c132743_g1	3114	273.490.404.422.933	5,99E+09	0.0	lamin Dm0-like	F:GO:0005198; C:GO:0005882
c132772_g2	2601	204.174.321.502.592	0.002685612	4.70E-103	catecholamines up-like	0
c132822_g1	3270	235.038.646.880.443	0.005180828	4,86E-43	nucleoside diphosphate-linked moiety X motif 6-like	F:GO:0016787
c132883_g1	4801	243.830.031.760.667	0.003051818	0.0	titin-like isoform X5	0
c132916_g1	4918	225.460.740.172.316	0.002263301	5,78E-121	RNA-binding 15B	0
c132920_g2	448	388.669.429.728.001	3,15E+08	9,21E-38	Methylenetetrahydrofolate reductase	0
c132965_g1	624	203.728.874.117.825	0.002642726	5,56E-36	probable cation-transporting ATPase 13A3	0
c132995_g2	2840	345.173.686.141.936	9,63E+09	1.30E-148	eukaryotic translation initiation factor 4 gamma 2-like	0
c133023_g1	4779	174.495.175.955.045	0.001690972	0.00E+00	Protein lap4	0
c133024_g2	1772	210.234.217.438.519	0.006014227	0.0	kelch 13	0
c133055_g1	4078	185.229.325.529.113	0.007397721	0.0	fatty acid synthase-like	F:GO:0016417; F:GO:0016491; P:GO:0008152; F:GO:0016297
c133077_g3	552	233.269.827.370.275	0.008014541	1,04E-15	lysosome-associated membrane glyco 1 isoform X2	0
c133079_g2	2551	280.955.399.372.294	0.001617487	0.0	clustered mitochondria homolog isoform X2	F:GO:0003723; C:GO:0005737; P:GO:0048312
c133093_g2	2643	240.135.938.101.988	2,24E+09	9,48E-143	probable deferrochelatase peroxidase	0
c133117_g3	1264	279.667.600.774.378	0.002251712	3,18E-10	carboxypeptidase N subunit 2	0
c133160_g1	806	180.471.930.434.134	0.009716875	6,13E-80	Ceramide glucosyltransferase	C:GO:0016020
c133164_g1	2998	1.763.720.661.944	0.007246522	1,31E-52	zinc finger CCCH domain-containing 14-like isoform X1	0
c133177_g1	2204	183.880.800.149.517	0.008151354	0.0	probable ATP-dependent RNA helicase DDX27	0
c133193_g1	4031	155.795.498.919.354	0.008351282	0.0	afadin-like isoform X5	0
c133251_g1	3521	256.344.446.563.429	0.006072313	0.00E+00	Pre-mRNA-splicing factor CWC22	0
c133279_g1	245	177.115.374.096.911	0.004862556	3.20E-19	GPI inositol-deacylase-like	0
c133352_g1	4627	193.977.541.394.919	0.004061023	0.00E+00	DNA-directed RNA polymerase, mitochondrial	0
c133359_g1	5521	234.344.320.430.771	0.000298538	0.0	HEAT repeat-containing 1	0
c133421_g1	2903	151.589.584.592.359	0.0080232	0.00E+00	Long-chain specific acyl- dehydrogenase, mitochondrial	0
c133432_g2	1861	419.606.851.610.523	9,35E+09	1,96E-83	probable transporter MCH1	P:GO:0055085; C:GO:0016020; C:GO:0016021
c133439_g4	2167	414.657.152.782.947	4,13E+09	0.00E+00	protoflagellar creatine kinase	F:GO:0005524; C:GO:0031514; P:GO:0016310; F:GO:0016301
c133495_g1	2636	154.087.363.819.485	0.004683438	0.00E+00	ATP-binding cassette sub-family F member 2-like	0
c133568_g1	2789	238.665.532.146.426	0.008874376	3.30E-74	ras-related Rab-27A-like	0
c133622_g1	3205	224.005.702.944.541	8,64E+09	1,14E-75	roquin-1 isoform X1	0
c133624_g1	3021	196.466.796.647.975	0.000266191	0.00E+00	chromodomain-helicase-DNA-binding Mi-2 homolog isoform X3	0
c133626_g1	4070	292.349.877.887.085	0.001232351	0.0	NAD(P) mitochondrial-like	0
c133728_g2	814	256.243.531.750.275	0.006042367	2,14E-06	Transmembrane 151B	0
c133746_g1	5941	189.204.393.312.735	0.003650837	3,07E-28	titin isoform X10	0
c133773_g1	329	229.228.718.936.109	0.006550672	6.00E-15	lysosomal thioesterase PPT2-A-like isoform X3	0
c133781_g1	606	274.558.462.331.951	2,52E+09	3,71E-115	tryptophan--tRNA cytoplasmic-like	F:GO:0005524; C:GO:0005737; P:GO:0006436; F:GO:0004830
c133811_g1	3958	249.074.223.444.098	0.002664087	1,25E-170	NCK-interacting with SH3 domain-like	0
c133818_g1	6154	202.626.502.996.762	0.000828427	1,31E-164	sialin-like isoform X1	0
c133829_g1	4757	2.479.723.816.441	2,46E+08	0.0	twitchin isoform X1	0
c133848_g2	4425	735.483.846.346.988	2,59E-06	3,59E-37	multiple coagulation factor deficiency 2 homolog	0
c133916_g4	2841	192.856.164.541.189	0.003157818	3,00E-47	calcium channel flower homolog isoform X1	C:GO:0016020
c133940_g1	4751	247.052.588.453.579	0.000350398	1.00E-151	CWF19 2	0
c133944_g1	4088	360.518.841.238.929	0.000330036	4,90E-67	G- coupled receptor Mth-like	0
c134003_g1	4034	168.501.579.736.506	0.00221006	2.60E-76	oral-facial-digital syndrome 1 -like	0
c134010_g1	3572	263.860.236.936.832	1,94E+07	0.0	ryanodine receptor-like isoform X6	F:GO:0005219; F:GO:0005509; C:GO:0016021; P:GO:0051209; C:GO:0005622
c134010_g2	3943	256.799.608.147.233	0.000922901	0.0	ryanodine receptor 44F-like	C:GO:0005623; F:GO:0005262; C:GO:0016020; P:GO:0070588; P:GO:0006874
c134082_g2	4634	210.653.067.662.075	0.000415983	0.0	twitchin-like isoform X13	F:GO:0016740
c134146_g1	1182	34.426.300.504.792	9,74E+08	9.80E-11	PREDICTED: septin-4-like	0
c134226_g2	1344	345.486.596.079.749	0.001031031	1,03E-84	alpha-1,3-mannosyl-glyco 4-beta-N-acetylglucosaminyltransferase C-like	P:GO:0005975; F:GO:0016758; C:GO:0016020
c134240_g1	1632	192.813.145.419.614	0.00032187	3.80E-48	hsc70-interacting isoform X2	0
c134267_g3	612	203.472.397.529.378	0.007353564	6.30E-34	3-mercaptopyruvate sulfurtransferase	F:GO:0016740
c134274_g1	5537	234.791.616.303.523	0.000449632	0.0	transmembrane 168 isoform X1	0
c134296_g1	5941	168.690.329.411.372	0.003415171	0.00E+00	PREDICTED: protein ELYS	0
c134297_g1	1300	219.888.474.891.344	0.000948353	0.0	fructose-1,6-bisphosphatase 1-like	P:GO:0005975; F:GO:0042132; P:GO:0016311
c134329_g2	848	327.308.779.782.676	0.003795747	7.40E-26	deoxyribonuclease TATDN2	0
c134329_g3	1893	19.829.707.278.731	0.002235807	6,72E-33	proton myo-inositol cotransporter-like isoform X1	F:GO:0022891; P:GO:0055085; C:GO:0016021
c134365_g2	1203	200.637.353.828.791	0.008651354	2.30E-14	collagen alpha-5(IV) chain-like	C:GO:0005581; F:GO:0005201
c134393_g2	740	177.129.867.188.894	0.008743557	1.50E-69	ribosomal rpl30	F:GO:0003735; C:GO:0005840; P:GO:0006412
c134440_g1	4557	209.199.798.258.627	0.000492649	8.00E-164	lipoyl synthase, mitochondrial-like	0
c134474_g1	3693	399.198.648.530.747	2,24E+00	3,35E-153	Protocadherin Fat 4	F:GO:0005509; C:GO:0016020; C:GO:0016021; P:GO:0007155; P:GO:0007156; C:GO:0005886
c134474_g3	665	334.133.868.587.411	8,64E+09	5,35E-16	cadherin-related tumor suppressor	0
c134499_g1	4117	19.524.675.581.889	0.000325053	0.00E+00	PAPSS_URECA ame: Full=Bifunctional 3 -phosphoadenosine 5 -phosphosulfate synthase Short=PAPS synthase Short=PAPSS ame: Full=Sulfurylase kinase Short=SK Includes: ame: Full=Sulfate adenylyltransferase ame: Full=ATP-sulfurylase ame: Full=Sulfate adenylate transferase Short=SAT Includes: ame: Full=Adenylyl-sulfate kinase ame: Full=3 -phosphoadenosine-5 -phosphosulfate synthase ame: Full=APS kinase ame: Full=Adenosine-5 -phosphosulfate 3 -phosphotransferase ame: Full=Adenylylsulfate 3 -phosphotransferase	F:GO:0005524; F:GO:0004020; F:GO:0004781; P:GO:0016310; P:GO:0000103
c134500_g1	3773	228.053.991.053.684	0.001429894	0.0	kelch 8 isoform X2	C:GO:0031463; P:GO:0016567
c134504_g2	3543	261.048.813.698.082	0.000251892	0.0	fatty acid synthase-like isoform X1	0
c134556_g1	2961	472.724.681.251.415	1,60E+02	0.0	ras and EF-hand domain-containing -like isoform X1	0
c134633_g3	4441	25.343.245.182.281	7,49E+08	5,84E-125	cytokine receptor-like factor 3 isoform X2	0
c134662_g1	956	215.336.883.861.066	2,66E+09	4.30E-51	Serrate RNA effector molecule	0
c134666_g3	5181	323.974.601.269.164	0.00013983	1,57E-49	serrate RNA effector molecule homolog isoform X2	0
c134690_g1	5520	170.926.554.634.811	0.006062314	0.0	glycogen debranching enzyme-like	P:GO:0005977; F:GO:0004133
c134721_g2	1317	195.630.608.472.097	0.006689471	2,21E-165	saccharopine dehydrogenase-like oxidoreductase isoform X1	C:GO:0016020
c134727_g3	4814	268.126.001.511.018	0.004590669	6,83E-157	BTB POZ domain-containing 17-like	0
c134821_g1	1696	171.779.620.678.381	0.009828933	0.0	small subunit processome component 20 homolog	C:GO:0044424
c134826_g1	5433	299.701.989.473.325	9,61E+09	0.00E+00	Titin	0
c134826_g2	4175	253.985.639.387.877	0.003612313	0.0	twitchin isoform X2	0
c134850_g1	5097	268.726.599.344.732	0.006371312	3.30E-54	PHD and RING finger domain-containing 1-like	0
c134854_g1	4086	241.551.719.706.564	0.006302753	3,46E-66	PHD and RING finger domain-containing 1-like	F:GO:0008270; F:GO:0046872
c134855_g2	1343	282.063.142.330.825	0.000156937	2,42E-142	patched domain-containing 3-like	0
c134857_g2	8323	196.112.600.798.291	0.007406674	1,16E-20	organic solute transporter subunit alpha-like isoform X1	0
c134885_g1	3470	172.699.469.202.259	0.004732939	5,65E-129	triblock copolymer TP12T	F:GO:0005509; C:GO:0016020; C:GO:0016021; C:GO:0005578
c134895_g2	905	310.283.366.622.551	0.001778793	0.0	sodium potassium-transporting ATPase subunit alpha-like isoform X1	P:GO:0006813; F:GO:0005524; P:GO:0006814; F:GO:0005391; F:GO:0046872; P:GO:0090662; C:GO:0016021; P:GO:0010248
c134954_g1	5117	332.875.835.905.197	5,09E+09	0.0	laminin subunit alpha	P:GO:0007155
c134975_g1	3104	192.333.568.248.785	0.001866202	0.0	pre-mRNA-processing-splicing factor 8	0
c134987_g1	5148	155.618.234.552.937	0.007525163	0.0	filamin-A-like isoform X2	0
c134998_g2	10013	262.270.187.736.866	0.000219598	0.0	laminin subunit gamma-1-like	P:GO:0044699
c135028_g1	5618	32.928.306.493.044	0.000955254	0.00E+00	CD109-like molecule	F:GO:0005524; F:GO:0046872; P:GO:0006556; F:GO:0004478; P:GO:0006730
c135045_g1	5579	169.243.083.620.766	0.003142538	0.0	thioester-containing 1	C:GO:0005615; F:GO:0004866; P:GO:0010951; C:GO:0005576
c135094_g1	4790	38.860.152.692.512	7,02E+07	0.00E+00	Titin	F:GO:0008061; P:GO:0006030; P:GO:0006508; C:GO:0005576; F:GO:0004222; F:GO:0008237
c135111_g1	9340	219.119.968.081.633	0.000322858	6.10E-126	FH2 domain-containing 1	0
c135130_g1	8632	222.572.330.157.053	0.000670128	0.00E+00	Lon protease , mitochondrial, partial	F:GO:0008270
c135200_g1	4062	214.087.648.628.687	0.004917945	1.80E-12	Collagen alpha-5(VI) chain	0
c135240_g1	4212	236.737.699.092.541	0.008308837	0.0	titin isoform X2	0
c135240_g2	8464	226.181.288.853.704	0.003006073	1,39E-44	titin isoform X1	0
c135245_g1	216	216.398.038.385.096	1,94E+09	0.0	inverted formin-2	0
c135250_g1	890	225.119.473.583.078	1,19E+09	8.10E-07	glutenin, high molecular weight subunit PW212-like	F:GO:0005524; P:GO:0070407; P:GO:0006515; C:GO:0005759; P:GO:0051131; F:GO:0004252; P:GO:0090296; P:GO:0034599; F:GO:0043565; F:GO:0004176
c135253_g2	1761	312.730.110.487.206	4,86E+04	6.30E-15	glutenin, high molecular weight subunit PW212-like	0
c135288_g1	246	446.176.414.411.931	1,41E+05	9.70E-19	arginine N-methyltransferase 1-like, partial	0
c135329_g2	634	370.478.859.009.857	2,50E+02	8.80E-88	ribosomal rpl26	F:GO:0005509; C:GO:0016020; C:GO:0016021; P:GO:0007155; P:GO:0007156; C:GO:0005886
c135340_g1	216	47.540.374.082.648	1,90E+08	1,14E-03	pyruvate dehydrogenase phosphatase regulatory mitochondrial	0
c135355_g1	890	240.776.256.652.778	0.002543213	8.1E-7	glutenin, high molecular weight subunit PW212-like	0
c135355_g3	980	338.613.080.038.946	2,78E+05	2.6E-12	glutenin, high molecular weight subunit PW212-like	0
c88695_g1	246	3.341.300.416.621	1,20E+07	9.7E-19	arginine N-methyltransferase 1-like, partial	0
c98826_g2	634	255.464.260.075.739	0.000393686	8.8E-88	ribosomal rpl26	F:GO:0003735; C:GO:0015934; P:GO:0006412
c106837_g1	1068	-312.800.747.802.994	0.00475201237697481	8.2E-94	WD repeat-containing C10orf79	0
c109662_g1	1679	-424.107.788.295.617	1,94E+09	9.9E-82	Peptidyl-prolyl cis-trans isomerase B	0
c110171_g1	735	-34.701.385.415.326	0.00129116968900715	2,09E-87	kinesin KIF9	F:GO:0000166
c112841_g1	279	-416.778.253.623.714	6,46E+09	3,68E-20	dynein heavy chain axonemal-like isoform X2	F:GO:0000166; F:GO:0016887; F:GO:0005524; F:GO:0003777; C:GO:0030286; P:GO:0007018
c113239_g1	1532	-430.265.684.248.105	0.000612516864175112	1,22E-06	sperm flagellar 1-like	0
c113343_g2	1498	-361.000.954.029.168	1,07E+09	0.0	coiled-coil domain-containing 65-like isoform X1	0
c114011_g1	717	-320.618.243.821.192	0.00104185958979501	2,59E-107	radial spoke head 14 homolog	0
c114358_g1	1073	-409.805.168.920.956	0.00059156403683411	1,40E-171	Dynein heavy chain axonemal	F:GO:0016887; F:GO:0005524; F:GO:0003777; C:GO:0030286; P:GO:0007018
c116103_g1	1087	-366.652.988.983.397	6,37E+08	0.0	dynein heavy chain axonemal-like	F:GO:0016887; F:GO:0005524; F:GO:0003777; C:GO:0030286; P:GO:0007018
c116389_g1	1251	-270.927.387.365.783	0.00360569960652419	9,62E-104	enkurin-like isoform X1	0
c116557_g1	1493	-233.173.416.841.432	0.00999294971490554	4,35E-83	calpain-B isoform X12	F:GO:0008234
c116860_g1	356	-285.800.231.270.686	0.00955516394891333	2.4E-20	MORN repeat-containing 3	0
c116977_g1	1902	-240.929.302.278.769	0.00130026908373923	0.0E0	coiled-coil domain-containing 176-like	0
c117573_g2	2100	-330.982.759.419.076	2,74E+09	0.0E0	adenylate kinase 7	0
c117751_g1	2164	-229.032.108.304.484	0.00248744917402163	3,56E-131	G1 S-specific cyclin-D2-like	0
c117828_g1	1345	-341.264.776.946.713	0.00990619574509934	1,77E-148	dynein heavy chain axonemal-	F:GO:0016887; F:GO:0005524; F:GO:0003777; C:GO:0005858; P:GO:0003341
c118344_g5	1228	-347.085.746.644.773	0.00922469897239842	6,14E-52	short transient receptor potential channel 4-like	0
c118422_g1	1279	-337.143.515.227.494	0.000896279114999271	5.5E-109	coiled-coil domain-containing 42 homolog	0
c118709_g1	1276	-328.847.317.443.819	9,24E+09	1,15E-16	UPF0691 C9orf116 homolog	0
c118745_g1	715	-370.683.329.217.376	0.000388151149666925	3,00E-10	protein-tyrosine kinase 6-like	F:GO:0000166; F:GO:0005524; F:GO:0004672; F:GO:0004674; P:GO:0006468
c119136_g1	632	-237.937.029.006.181	0.000683079291029482	3,38E-94	EF-hand domain-containing family member C2-like	0
c119722_g2	412	-331.891.273.101.538	0.000340314368552772	2.4E-25	ropporin-1-like protein	0
c119797_g2	1834	-37.530.608.915.722	0.00127326599264529	4.8E-48	PREDICTED: myosin-9-like	0
c120136_g2	1840	-473.833.198.206.667	4,36E+05	9,20E-81	hemocyte -glutamine gamma-glutamyltransferase-like	P:GO:0018149
c120216_g3	1320	-278.973.822.410.236	4,53E+09	9,08E-71	cilia- and flagella-associated 77-like isoform X6	0
c120731_g1	2388	-288.138.488.289.031	6,38E+06	0.0	dynein heavy chain axonemal-like	F:GO:0003777; F:GO:0005509; C:GO:0030286; P:GO:0007018
c121080_g1	2570	-403.298.608.650.762	0.00147144871449322	1.7E-33	Deleted in malignant brain tumors 1	C:GO:0016020; F:GO:0005044; P:GO:0006898
c121159_g1	2237	-404.097.252.748.044	1,71E-02	1,38E-08	glutamate-rich 6-like	0
c121222_g2	598	-487.699.584.211.961	1,31E+09	2,72E-30	reticulocalbin-2	0
c121254_g2	1645	-259.731.280.889.792	0.00398839520483548	0.0	RIB43A-like with coiled-coils 2	0
c121647_g1	1177	-367.164.060.835.932	6,55E+08	8.8E-43	amphicalbin	0
c121907_g2	2063	-332.446.596.803.747	3,90E+01	2.6E-168	Leucine-rich repeat-containing 63	0
c121930_g1	207	-359.364.136.479.781	0.00027567878061738	2.4E-17	kinesin KIF9-like isoform X1	0
c121935_g2	2509	-73.284.371.131.621	5,45E-08	3.9E-140	two pore calcium channel 1-like	0
c121948_g1	1567	-287.447.255.302.013	6,03E+07	4.7E-114	Radial spoke head 9	0
c121972_g1	1693	-289.420.529.080.816	4,02E+09	1.7E-107	calumenin-like isoform X1	0
c122919_g3	1977	-622.085.814.619.431	7,48E+08	3.5E-84	probable G- coupled receptor 157 isoform X1	0
c122962_g1	1713	-240.244.617.566.183	0.000194626661896178	8.9E-124	Cytochrome b5 domain-containing 1	0
c122998_g1	2153	-29.206.106.883.933	8,14E+09	8,49E-146	mechanosensory 2-like isoform X3	C:GO:0016021
c123299_g1	603	-393.763.563.845.017	1,86E+00	2.4E-50	radial spoke head 3 homolog	0
c123333_g1	2848	-715.205.574.745.755	1,69E-07	0.0E0	dynein heavy chain axonemal-like	0
c123432_g1	2500	-43.075.529.531.284	9,61E+01	7,26E-49	ATPase family AAA domain-containing 2 isoform X3	0
c123534_g1	2054	-460.313.696.417.544	0.000124112346615926	0.0E0	HORMA domain-containing 1-like	0
c123577_g3	1257	-289.145.311.442.747	0.00820041581562974	0.0E0	Coiled-coil domain-containing 39	0
c123627_g3	1449	-262.741.760.118.978	0.000891110255782384	9.1E-72	dynein light chain Tctex-type 1-like	0
c123711_g1	323	-417.975.557.583.943	4,10E+09	4.0E-10	tetratricopeptide repeat 27-like	0
c124162_g1	1142	-417.748.489.141.364	1,04E+07	1,70E-34	Coiled-coil domain-containing 79	0
c124760_g2	2238	-248.992.099.803.868	0.000392748309039287	0.0E0	coiled-coil domain-containing 146-like	0
c125307_g1	4174	-327.401.758.509.365	0.000279598168970699	0.0E0	WD repeat-containing 65-like	0
c125318_g1	3254	-337.200.722.547.884	1,11E+02	0.0	ankyrin repeat and EF-hand domain-containing 1-like isoform X1	F:GO:0005509
c125360_g1	1875	-274.090.670.151.819	0.00256894605718704	8.4E-28	PREDICTED: formimidoyltransferase-cyclodeaminase	0
c125459_g1	2549	-340.942.166.714.526	4,01E+08	1.8E-163	Sperm-associated antigen 17	0
c125571_g1	4032	-426.179.276.968.123	1,99E+05	3,78E-110	G2 mitotic-specific cyclin-B3-like isoform X2	0
c126013_g2	3435	-456.755.696.566.258	3,75E+08	0.0	dynein beta flagellar outer arm-like	0
c126027_g2	2454	-35.071.738.318.981	0.00128691641384298	0.0	malate dehydrogenase 1B	0
c126193_g1	1884	-261.565.295.773.242	0.000709811016047326	0.0	IQ domain-containing H	0
c126473_g1	2320	-729.553.518.937.126	1,08E-04	1,53E-46	beta-1,4-N-acetylgalactosaminyltransferase bre-4-like	F:GO:0005509
c126499_g1	1231	-286.208.971.465.464	0.00995104802958446	0.0	zinc finger MYND domain-containing 12-like	0
c126508_g1	2847	-369.471.404.649.792	0.00025170991296679	0.0E0	axonemal dynein light chain domain-containing 1-like	0
c126534_g1	642	-349.725.192.193.259	2,52E+08	2,30E-107	axonemal dynein heavy chain	0
c126585_g1	796	-416.657.257.579.001	4,00E+08	2,87E+00	dynein heavy chain axonemal-like isoform X3	F:GO:0003777; F:GO:0005509; C:GO:0030286; P:GO:0007018
c126659_g1	857	-341.013.610.221.504	0.000418382942632593	1,39E-102	translin-associated factor X-interacting 1	0
c126659_g2	1269	-325.705.940.671.084	7,31E+09	2,15E-07	translin-associated factor X-interacting 1-like	0
c126721_g2	2651	-454.989.075.166.446	4,14E+05	1,23E-46	Ig-like V-type domain-containing FAM187A	0
c126772_g1	1775	-562.264.986.613.004	1,65E+00	4,50E-122	PREDICTED: protein FAM166B-like	0
c127027_g1	4131	-573.549.419.030.552	2,89E-01	1.7E-100	Sodium hydrogen exchanger 10	0
c127180_g1	670	-489.812.477.475.032	5,77E+07	2.3E-76	mitochondrial uncoupling 2	0
c127208_g1	3599	-248.896.717.177.597	0.00156755170733448	1.6E-165	Retinoblastoma-associated protein	0
c127539_g1	204	-337.145.892.746.316	0.00476334469536481	1.4E-13	Dynein heavy chain 1, axonemal	F:GO:0005524; F:GO:0003777; C:GO:0030286; P:GO:0007018

	Upregulated in Male	c127815_g2	681	-633.848.177.973.243	4,94E-13	2,10E-33	leucine-rich repeat soc-2-like	0
		c128079_g1	861	-252.179.544.960.423	0.00806948030594032	7,55E-136	axonemal dynein heavy chain	F:GO:0003777; C:GO:0030286; P:GO:0007018
		c128152_g2	869	-34.897.410.466.004	7,85E+09	2,50E-84	magnesium proton exchanger 1-like isoform X9	P:GO:0055085; C:GO:0016020; P:GO:0030001
		c128184_g2	1714	-418.976.731.936.536	0.000196904462763116	8.3E-91	Rhomboid family member 1	0
		c128203_g1	2173	-374.759.064.082.203	0.00025187075364425	0.0	dynein heavy chain axonemal	F:GO:0003777; F:GO:0005509; C:GO:0030286; P:GO:0007018
		c128242_g1	1333	-343.004.224.724.897	3,35E+08	9,16E-05	leucine-rich repeat-containing 56-like	0
		c128275_g1	472	-295.501.554.722.407	7,71E+06	2.6E-12	ribosomal L4,	0
		c128430_g1	727	-326.193.416.257.574	6,84E+09	7,13E-16	interaptin-like isoform X3	0
		c128601_g1	4348	-209.274.962.246.651	0.00451904306613639	0.0E0	DNA topoisomerase 2-beta	0
		c128910_g2	1053	-659.320.225.285.299	2,23E-05	1.4E-171	dynein heavy chain 5, axonemal-like, partial	0
		c129069_g1	2770	-304.155.628.101.928	4,46E+06	0.0E0	dynein heavy chain 3, axonemal-like	F:GO:0016887; F:GO:0005524; F:GO:0003777; C:GO:0030286; P:GO:0007018
		c129183_g1	1645	-376.278.490.854.658	0.000227748672373039	0.0	dynein heavy chain axonemal-like	F:GO:0016887; F:GO:0005524; F:GO:0003777; C:GO:0005858; P:GO:0003341
		c129183_g2	2680	-384.116.863.152.856	2,70E+08	0.0	dynein heavy chain axonemal-like	0
		c129243_g1	2251	-432.297.005.755.661	4,55E+05	1,52E-152	dynein beta ciliary isoform X6	F:GO:0003777; C:GO:0030286; P:GO:0007018
		c129261_g3	2583	-373.578.719.360.724	2,48E-01	2,62E-42	opioid growth factor receptor 1	C:GO:0016020; F:GO:0004872
		c129271_g1	1601	-290.924.829.628.476	0.0028440094057934	0.0	coiled-coil domain-containing 108-like	0
		c129279_g1	1960	-398.353.146.259.226	0.000139424579896786	0.0	coiled-coil domain-containing protein 37-like isoform X1	0
		c129304_g1	952	-32.610.213.292.723	0.00950792823194067	1.6E-12	Zinc finger 26	0
		c129407_g1	2442	-357.562.098.569.746	9,27E+09	0.0	EF-hand calcium-binding domain-containing 12-like	F:GO:0005509
		c129664_g2	2933	-346.953.690.595.607	0.00115951576601081	0.0	abnormal spindle-like microcephaly-associated homolog	0
		c129666_g1	3959	-256.143.128.902.962	0.00922103272583724	0.0	serine threonine- kinase PLK4-like	F:GO:0000166; F:GO:0005524; F:GO:0004672; P:GO:0006468
		c129739_g3	1658	-327.276.798.325.312	0.0020371660552937	1.1E-37	cation channel sperm-associated 2-like	0
		c129755_g2	2205	-255.824.316.582.191	0.00123202112737333	0.0	fizzy-related homolog	0
		c129791_g1	4034	-230.368.650.561.828	0.00379574664402056	0.0E0	Coiled-coil domain-containing 40	0
		c129803_g1	4335	-514.687.755.640.285	7,62E+03	0.0E0	dynein beta chain, flagellar outer arm-like	0
		c129936_g1	305	-310.465.805.201.316	0.000115296427604337	9,00E-16	daf-12-interacting protein 1-like isoform X2	0
		c129957_g1	1314	-475.165.828.445.966	1,17E+08	8,19E-07	coiled-coil domain-containing 73-like	0
		c130044_g1	2105	-237.033.228.898.706	0.00356159215337634	0.0	Dynein heavy chain axonemal	F:GO:0016887; F:GO:0005524; F:GO:0003777; C:GO:0030286; P:GO:0007018
		c130119_g3	2211	-295.981.753.667.436	5,08E+09	0.0	cilia- and flagella-associated 74-like	0
		c130127_g1	1059	-330.649.474.843.833	0.00628798096886634	3,84E-98	E3 ubiquitin- ligase CCNB1IP1-like	0
		c130176_g2	3911	-438.562.238.717.942	3,26E+04	0.0	dynein heavy chain axonemal-like isoform X2	0
		c130334_g1	945	-214.384.623.954.155	0.00186944684099667	2.2E-125	parkin coregulated gene homolog	0
		c130420_g2	268	-391.902.480.166.267	3,36E+06	3.4E-15	probable peptidyl-tRNA hydrolase	C:GO:0005739; P:GO:0032543; F:GO:0004045
		c130491_g1	2930	-338.348.679.440.803	2,00E+09	0.0E0	Sperm-associated antigen 17	0
		c130802_g1	1460	-346.142.142.014.645	0.00025170991296679	5.02E-115	coiled-coil domain-containing 178-like isoform X2	0
		c130991_g1	2556	-437.798.348.825.002	5,12E+08	0.0E0	Dynein-1-beta heavy chain, flagellar inner arm I1 complex	0
		c130991_g2	1196	-442.470.479.206.069	3,28E+08	1,23E-173	dynein heavy chain axonemal-like	F:GO:0005524; F:GO:0003777; C:GO:0030286; P:GO:0007018
		c131065_g2	866	-414.053.015.807.137	0.00248744917402163	9,06E-23	potassium channel subfamily K member 5-like	0
		c131149_g1	1001	-388.961.738.591.343	0.00196926957714458	4.7E-61	leucine-rich repeat-containing 72-like	0
		c131188_g1	2024	-353.591.931.092.395	0.00210927757103624	1,47E-64	sex determining Fem-1 like	0
		c131189_g2	442	-496.966.717.491.846	6,22E+01	3,83E-70	dynein beta ciliary-like	F:GO:0016887; F:GO:0005524; F:GO:0003777; C:GO:0030286; P:GO:0007018
		c131326_g1	1893	-337.125.751.655.167	2,97E+04	3.1E-161	furin-like convertase	F:GO:0016787
		c131369_g1	3131	-282.624.521.155.624	4,46E+09	0.0	cilia- and flagella-associated 157	0
		c131446_g1	243	-415.118.068.131.961	1,62E+08	4,30E-11	cilia- and flagella-associated 69 isoform X1	0
		c131475_g1	2733	-415.044.849.443.751	0.00263687295434905	2,32E-02	c-binding -like	C:GO:0016020; C:GO:0016021
		c131495_g1	2009	-267.235.254.328.322	0.00120221626513809	0.0	cilia- and flagella-associated 43-like isoform X2	0
		c131551_g1	3479	-342.121.436.595.761	0.000802154821144589	0.0	cilia- and flagella-associated 44-	0
		c131736_g1	303	-249.908.585.087.948	0.000682076255223332	3,89E-27	cilia- and flagella-associated 221-like	0
		c131778_g1	263	-303.388.490.346.994	2,57E+09	5,43E-07	adenylate kinase 9-like	0
		c131811_g1	2720	-354.367.564.469.344	0.00546367867344397	0.0E0	dynein heavy chain 17, axonemal-like	0
		c131819_g1	3273	-258.091.182.831.972	0.00711127268420958	0.0	MAATS1 isoform X1	0
		c131931_g1	3737	-45.357.700.896.385	1,43E+06	0.0	dynein heavy chain axonemal-like	F:GO:0016887; F:GO:0005524; F:GO:0003777; C:GO:0030286; P:GO:0007018
		c131931_g2	931	-39.225.548.420.463	5,20E+09	2,64E-163	dynein heavy chain axonemal-like isoform X1	F:GO:0016887; F:GO:0005524; F:GO:0003777; C:GO:0030286; P:GO:0007018
		c132002_g1	532	-350.718.144.702.607	0.0028440094057934	9.1E-17	cAMP-dependent kinase type II regulatory subunit	P:GO:0016310; F:GO:0016301
		c132164_g1	1036	-354.119.156.037.594	5,07E+09	2,80E-92	adenylate kinase	F:GO:0004017; F:GO:0005524; P:GO:0046939; C:GO:0005737; P:GO:0046034
		c132246_g1	2105	-492.918.446.820.975	4,44E+07	1,17E-89	sodium potassium calcium exchanger 3-like isoform X1 [Orbicella faveolata]	P:GO:0055085; C:GO:0016021
		c132246_g2	2190	-3.721.777.099.663	2,39E+06	2,00E-82	sodium potassium calcium exchanger 3-like	P:GO:0055085; C:GO:0016021
		c132420_g2	3981	-322.884.182.366.936	3,08E+08	0.0	WD repeat-containing 66	0
		c132427_g1	4147	-323.686.828.427.872	0.00477288766055564	0.0	hydrocephalus-inducing -like	0
		c132492_g1	1961	-258.505.985.899.352	0.000116512937808963	0.0E0	WD repeat-containing 16-like	0
		c132547_g1	4407	-270.128.313.636.421	0.000108279301348378	3,25E-152	actin-binding anillin-like isoform X5	0
		c132637_g1	1738	-335.294.874.129.888	1,07E+08	2,94E-70	Golgi pH regulator-like	C:GO:0016021
		c132787_g1	1315	-48.877.416.304.129	1,64E-02	1,33E-35	coiled-coil domain-containing 62-like isoform X2	0
		c132820_g1	4288	-546.624.149.101.941	3,70E+02	1,00E+00	WD repeat-containing 88	0
		c132839_g1	2648	-247.287.348.648.799	0.00264545110422331	2.5E-34	Nucleolar and spindle-associated 1	0
		c132925_g1	552	-386.535.591.162.014	3,27E+07	5.4E-47	WD repeat-containing 17	0
		c133022_g2	2156	-41.988.294.283.377	0.00618536859221418	4.5E-39	sushi, nidogen and EGF-like domain-containing 1	0
		c133138_g1	3259	-409.905.587.701.655	3,38E+06	0.0	dynein heavy chain axonemal-like	F:GO:0005524; F:GO:0003777; C:GO:0030286; P:GO:0007018
		c133138_g2	1625	-351.736.466.392.708	5,15E+07	8,23E-142	dynein heavy chain axonemal-like	0
		c133197_g1	4049	-586.334.581.951.636	6,38E+06	1.2E-61	GBR-2A protein	0
		c133214_g1	4925	-246.480.692.281.154	0.0010199080683281	0.0	structural maintenance of chromosomes 3-like isoform X1	C:GO:0005634; F:GO:0005524; P:GO:0006281; P:GO:0007064; C:GO:0005694; F:GO:0003682
		c133229_g1	225	-398.004.549.037.343	6,66E+09	6,32E-18	WD repeat-containing 63 isoform X1	0
		c133283_g1	533	-196.713.374.936.273	0.007301390742693	1.7E-84	Dynein intermediate chain 2, ciliary	0
		c133332_g3	2644	-377.261.434.629.546	1,26E+08	0.0	ankyrin repeat and MYND domain-containing 1-like isoform X11	F:GO:0046872
		c133428_g1	1003	-355.282.216.115.329	2,56E+07	2.3E-133	Dynein heavy chain 3, axonemal	0
		c133528_g1	1468	-322.977.997.842.836	0.00757007159847768	3.2E-121	predicted protein	0
		c133575_g1	6146	-311.828.293.219.692	4,56E+09	1.6E-39	serine threonine- phosphatase 6 regulatory ankyrin repeat subunit A-like	0
		c133602_g1	4691	-852.073.990.539.738	3,80E-10	0.0E0	neurogenic locus Notch -like	0
		c133721_g3	4869	-247.655.433.047.331	0.000387723517487575	0.0E0	Tetratricopeptide repeat 6	0
		c133935_g1	4016	-286.573.186.396.388	0.000100242025163749	3,74E-06	zona pellucida-binding 2 isoform X4	P:GO:0007339; C:GO:0016020; C:GO:0016021; C:GO:0005576
		c133942_g2	6310	-481.862.976.962.074	2,10E+05	0.0	dynein heavy chain domain-containing 1-like	F:GO:0000166; F:GO:0005524; F:GO:0003777; P:GO:0007018
		c133986_g1	2837	-374.075.149.054.102	9,59E+07	0.0	dynein heavy chain axonemal-like	F:GO:0016887; F:GO:0005524; F:GO:0003777; C:GO:0030286; P:GO:0007018
		c134000_g1	2417	-440.725.391.669.289	4,53E+06	0.0	WD repeat-containing KIAA1875-like isoform X3	0
		c134000_g2	2577	-319.545.285.981.943	0.00970111115963902	2,19E-110	WD repeat-containing	0
		c134176_g3	606	-384.679.707.736.825	3,50E+07	5.7E-48	coiled-coil domain-containing 11-like	0
		c134288_g1	5557	-326.842.653.033.092	0.00903161904926594	0.0	hydrocephalus-inducing isoform X8	0
		c134289_g1	6258	-283.752.912.952.989	4,55E+08	8,24E-118	cyclic AMP-dependent transcription factor ATF-6 beta-like isoform X2	0
		c134317_g1	3505	-351.528.347.034.999	3,75E+09	0.0E0	Deleted in lung and esophageal cancer 1	0
		c134319_g1	6378	-684.827.895.846.988	1,42E-03	0.0	dynein heavy chain axonemal-like	0
		c134326_g1	2952	-361.433.174.082.339	2,44E+00	0.0	hydrocephalus-inducing -like	P:GO:0003341
		c134426_g2	1984	-601.570.663.477.448	3,71E+07	0.0E0	tubulin beta chain-like isoform 1	0
		c134630_g1	3596	-491.823.517.209.697	1,34E+06	0.0	dynein-1-beta heavy flagellar inner arm I1 complex	0
		c134630_g2	1929	-362.956.334.081.292	0.00343516182628184	4.7E-108	Dynein-1-beta heavy chain, flagellar inner arm I1 complex	F:GO:0003777; C:GO:0031514; C:GO:0030286; P:GO:0007018
		c134671_g1	6740	-475.041.446.151.564	2,24E+00	6,54E-147	kelch diablo	0
		c134688_g3	923	-339.482.087.195.583	4,66E+09	7.0E-29	IQ domain-containing D-like	0
		c134733_g2	3362	-604.431.382.766.696	5,47E+00	4.7E-7	tolloid 1-like	0
		c134780_g2	1913	-307.678.559.038.272	0.00481646455975457	8,90E-109	SUN domain-containing 2-like isoform X6	0
		c134883_g1	1762	-228.218.182.307.968	0.00530119944543002	9,44E-64	pericentriolar material 1 -like isoform X3	0
		c134968_g1	5928	-437.417.715.241.304	0.00324603460955053	0.0	Cyclic nucleotide-gated cation channel beta-1	F:GO:0005216; P:GO:0034220; C:GO:0016021
		c135075_g1	3472	-479.460.791.560.174	1,84E+07	0.0	cilia- and flagella-associated 54-like	0
		c135075_g2	6755	-385.340.198.482.384	2,66E+09	0.0	cilia- and flagella-associated 54	0
		c135140_g1	3772	-262.032.803.285.096	3,72E+09	0.0	dynein heavy chain axonemal	F:GO:0000166; F:GO:0017111
		c135140_g2	1526	-400.828.876.084.682	9,44E+07	0.0E0	dynein beta chain, ciliary-like	0
		c135143_g1	4551	-304.106.667.358.937	0.000195919511773513	0.0E0	Armadillo repeat-containing 4	0
		c135152_g1	2447	-307.952.624.205.068	0.000183241826628635	9,17E-17	Fanconi anemia group M	0
		c135231_g1	5264	-372.548.632.886.786	1,36E+06	0.0	dynein heavy chain axonemal-like	0
		c135231_g2	2988	-477.479.667.234.351	6,95E+03	0.0	dynein heavy chain axonemal-like isoform X2	F:GO:0016887; F:GO:0005524; F:GO:0003777; C:GO:0005858; P:GO:0003341
		c135231_g3	2733	-458.873.199.048.737	3,15E+04	0.0	dynein heavy chain axonemal-like isoform X2	0
		c135304_g1	7227	-372.260.641.315.466	3,02E+08	0.0	dynein heavy chain axonemal-like isoform X3	F:GO:0016887; F:GO:0005524; F:GO:0003777; C:GO:0030286; P:GO:0007018
		c135304_g2	4168	-372.965.552.835.066	3,20E+08	0.0	dynein heavy chain axonemal-	0
		c135306_g1	4893	-264.328.680.925.745	1,09E+09	0.0	dynein heavy chain axonemal-like	F:GO:0016887; F:GO:0005524; F:GO:0003777; C:GO:0005858; P:GO:0001539; P:GO:0007018
		c135306_g3	8688	-38.133.848.691.997	0.000274267998376684	0.0E0	Dynein heavy chain 6, axonemal	0
		c135307_g1	355	-171.877.385.075.521	0.00353455960687908	3.8E-22	ankyrin repeat domain-containing 26-like	0
		c99358_g1	726	-274.463.201.983.496	0.00754864299282619	2.8E-73	radial spoke head 4 homolog A-like isoform X2	0
**ANTERIOR STOLON vs POSTERIOR STOLON**	Upregulated in Anterior Stolon	c114841_g1	1171	428.220.265.231.915	3,55E+05	1,80E-99	retinal-binding -like	C:GO:0016021; P:GO:0006810
		c125824_g1	232	29.726.359.840.674	0.00342548372608737	4.6E-12	rhabdomeric opsin	F:GO:0004930; P:GO:0007186; F:GO:0009881; C:GO:0016021; P:GO:0018298; P:GO:0007601; P:GO:0007602
		c132451_g1	2527	195.196.697.991.964	0.00342548372608737	4.9E-91	TRPC channel, partial	F:GO:0005262; C:GO:0016020; P:GO:0070588
		c133468_g1	504	1.826.465.802.219	0.00751213498061012	3,00E-28	deleted in autism protein 1 homolog [Lingula anatina]	0
