# Supplementary material for: Delegating Sex: Differential Gene Expression in Stolonizing Syllids Uncovers the Hormonal Control of Reproduction
Source: Genome Biol Evol. 2018 Dec 11;11(1):295–318. doi: 10.1093/gbe/evy265 (PMC6350857; doi:10.1093/gbe/evy265)
Supplement: Supplementary Data [file evy265_supp.zip › Supplementary Material legends.docx]

**Supplementary Material**

**Supplementary File S1.** General statistics of the ‘reference’ de novo transcriptomes REFSOM and REFTOTOREP. Total number of trimmed reads used for the assemblies (Total Reads), number of aligned reads for each tissue (Aligned Reads), percentage of aligned reads for each tissue, number of aligned transcripts (Align. Transcr.: including genes and isoforms collapsed into genes), number of genes, percentage og genes, parameter N50, number of transcripts with blast hit against proteins of metazoans (Hit_Mtz), number of annotated transcripts from metazoans (Annot), percentage of GC (GC%), median transcript length (MTL), average transcript length (ATL) and number of assembled bases expressed as Mb.

**Supplementary File S2.** (A) Assigned Gene Ontology (GO) terms for each transcriptome including 3 different levels: CC, BP and MF. (B) GO-term enrichment analysis based on the Fisher’s tests results of pairwise comparisons between both transcriptomes, showing the 36 processes overrepresented in REFSOM, and (C) the 8 processes overrepresented in REFTOTREPRO.

**Supplementary File S3**. BUSCO (Benchmarking Universal Single-Copy Orthologs) analysis for the quality of *Syllis magdalena* *de novo* transcriptome assemblies and their annotation completeness, using 65 metazoan species (metazoan_odb9 database) and 978 Single-Copy orthologs for each. (A) REFSOM and (B) REFTOTREPRO

**Supplementary File S4.** (A) Heatmap and (B) correlation matrix of differentially expressed genes from all the tissues and conditions analyzed.

**Supplementary File S5**. Volcano plots (left) and principal component analyses (PCAs) (right) showing the differentially expressed genes (annotated and not annotated genes) from pairwise comparisons of somatic tissues between reproductive and non-reproductive individuals (A) and somatic and reproductive tissues between females and males (B).

**Supplementary File S6.** Differentially expressed genes with blast hit, and further putative annotations (GO terms), from the pairwise comparisons of somatic tissues (anterior part, proventricle, final segments) between reproductive and non-reproductive individuals (REFSOM transcriptome).

**Supplementary File S7.** Differentially expressed genes with blast hit, and putative annotations (GO terms), from the pairwise comparisons of somatic tissues (anterior part, proventricle, final segments) and reproductive tissues (stolons) between female and male individuals (REFTOTREPRO transcriptome).

**Supplementary File S8.** Phylogenetic reconstruction of the protein alignment for all the paralogs of the genes *ovochymase* and *chymotripsin* found in our samples. *Ovochymase 1* was differentially expressed in the final segments of reproductive females, and *ovochymase 2* (clearly a *chymotrypsin* in this hypothesis) was differentially expressed in P of reproductive females.

**Supplementary File S9.**Phylogenetic reconstruction of the protein alignment for the DEAD-box helicases *vasa* and *PL10* showing the two homologs of *vasa* (*vasa 1* differentially expressed) and one for *PL10*.

**Supplementary File S10.** Phylogenetic reconstruction of the protein alignment for the Farnesoate receptor (A) and the different enzymes involved in the synthesis of methyl farnesoate (B–E). *Farnesoid X receptor* (FXr) (A), *Farnesyl pyrophosphate synthase* (FPPS) (B), Farnesyl phosphatase (FP) (C), *Farnesol oxidase/dehydrogenase* (SDR11) (D), and *Farnesal dehydrogenases* (ALDHE3) (D).
