# Supplementary figures and images for: Delegating Sex: Differential Gene Expression in Stolonizing Syllids Uncovers the Hormonal Control of Reproduction
Source: Genome Biol Evol. 2018 Dec 11;11(1):295–318. doi: 10.1093/gbe/evy265 (PMC6350857; doi:10.1093/gbe/evy265)

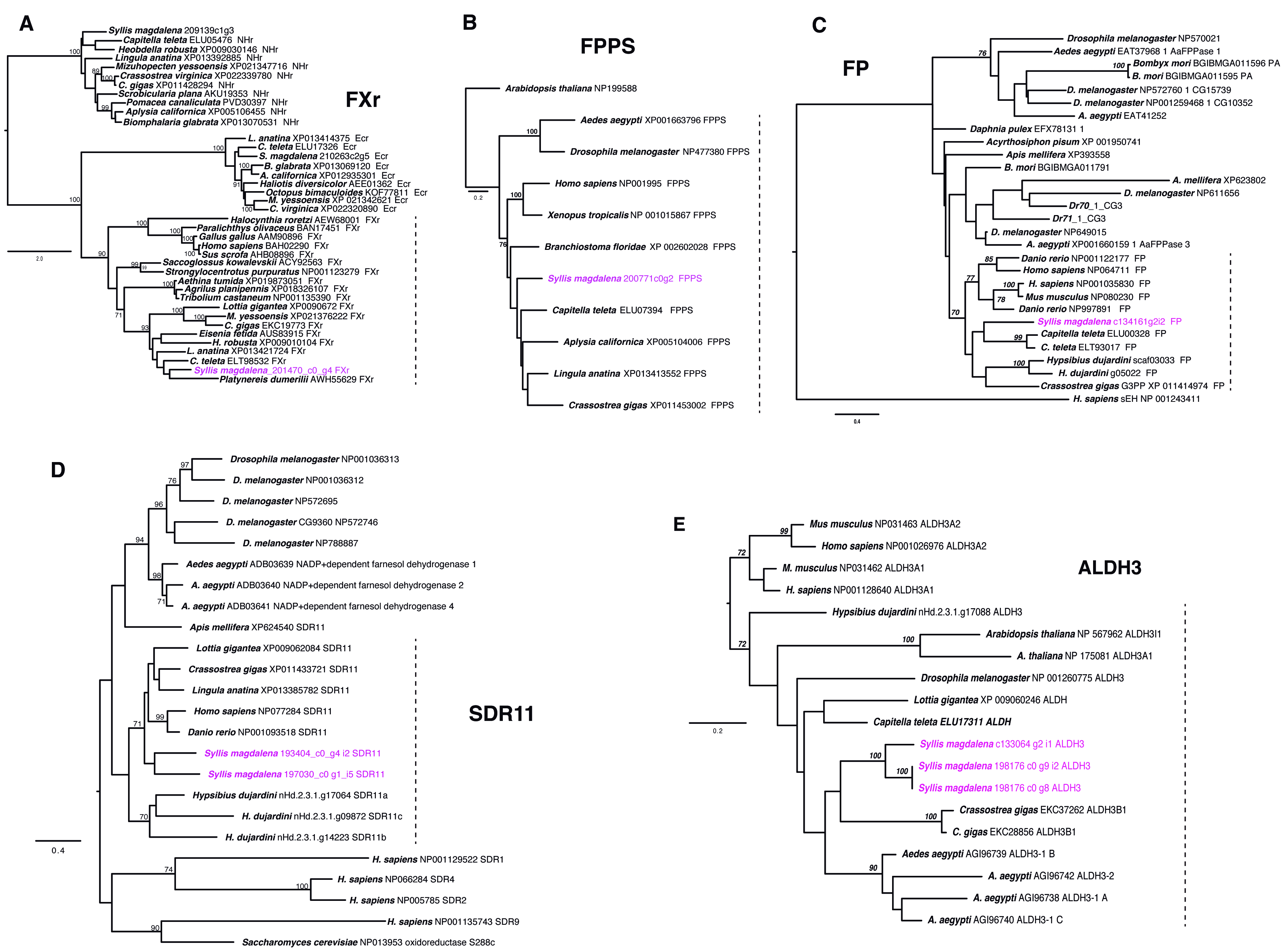

Supplement: Supplementary Data [file evy265_supp.zip › SuppFile_S10_MF_trees.tif]

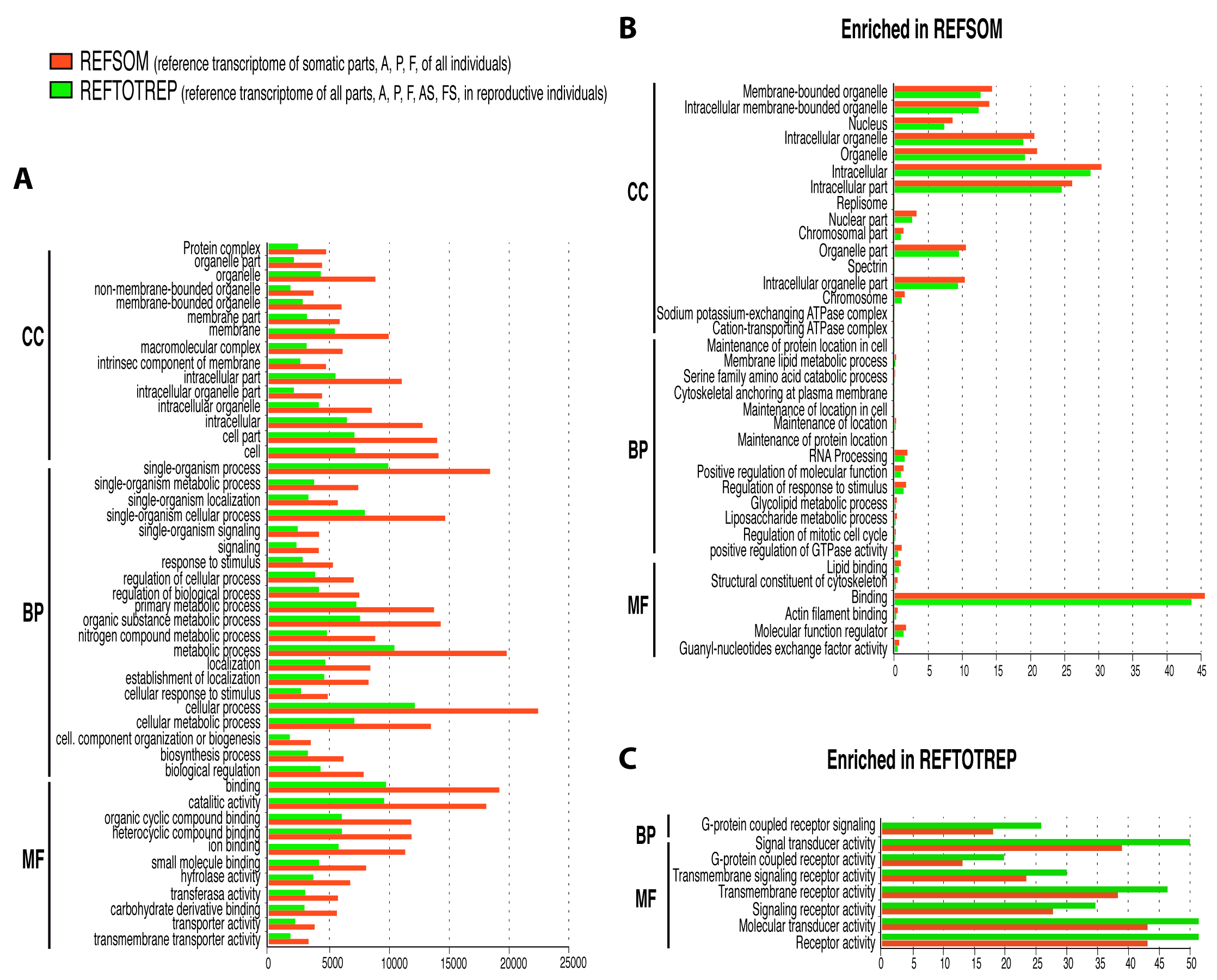

Supplement: Supplementary Data [file evy265_supp.zip › SuppFile_S2_Enrichment_Graph_REFSOM&REFTOTREP_GOOD2.tif]

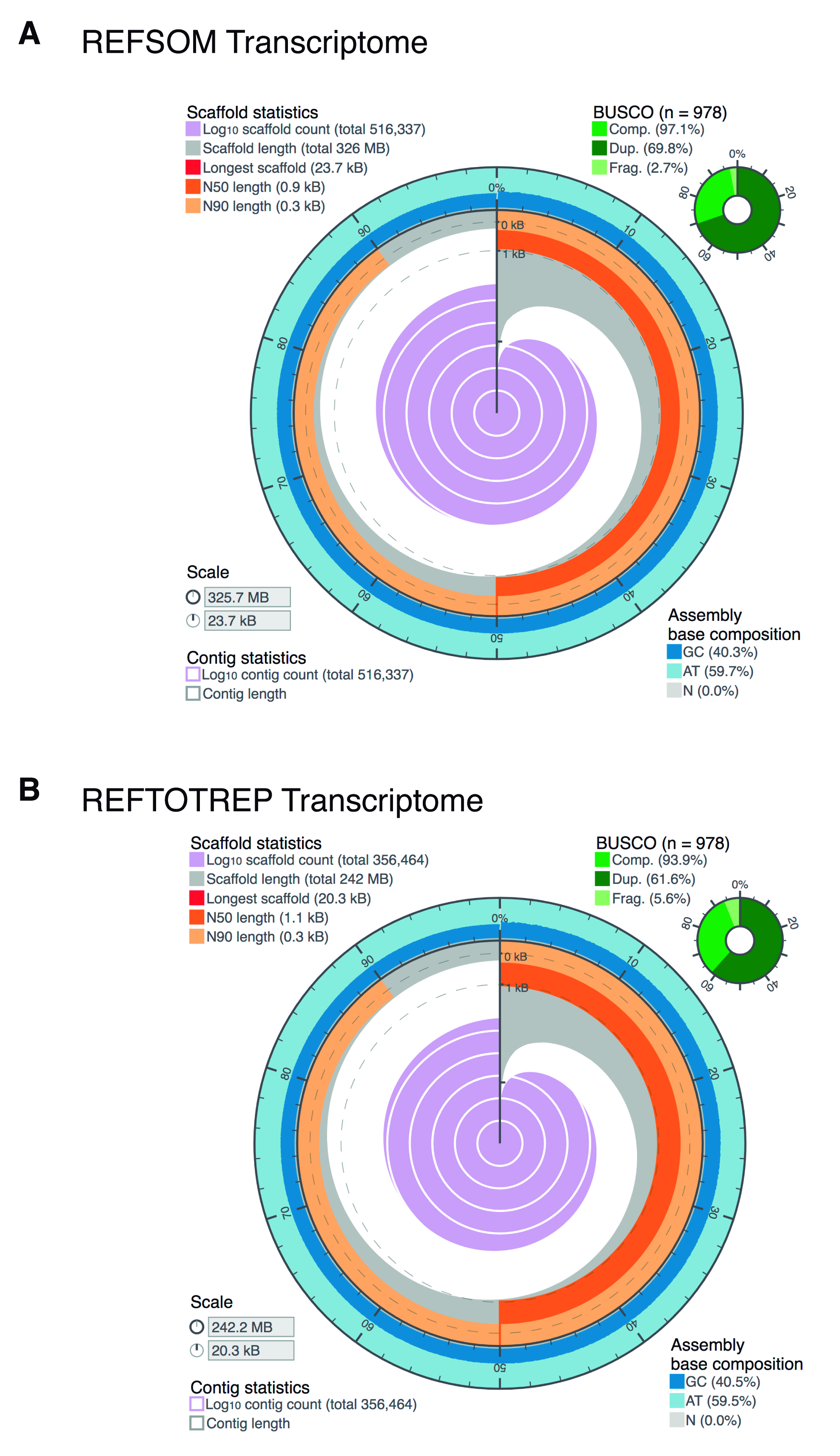

Supplement: Supplementary Data [file evy265_supp.zip › SuppFile_S3_BUSCO.tif]

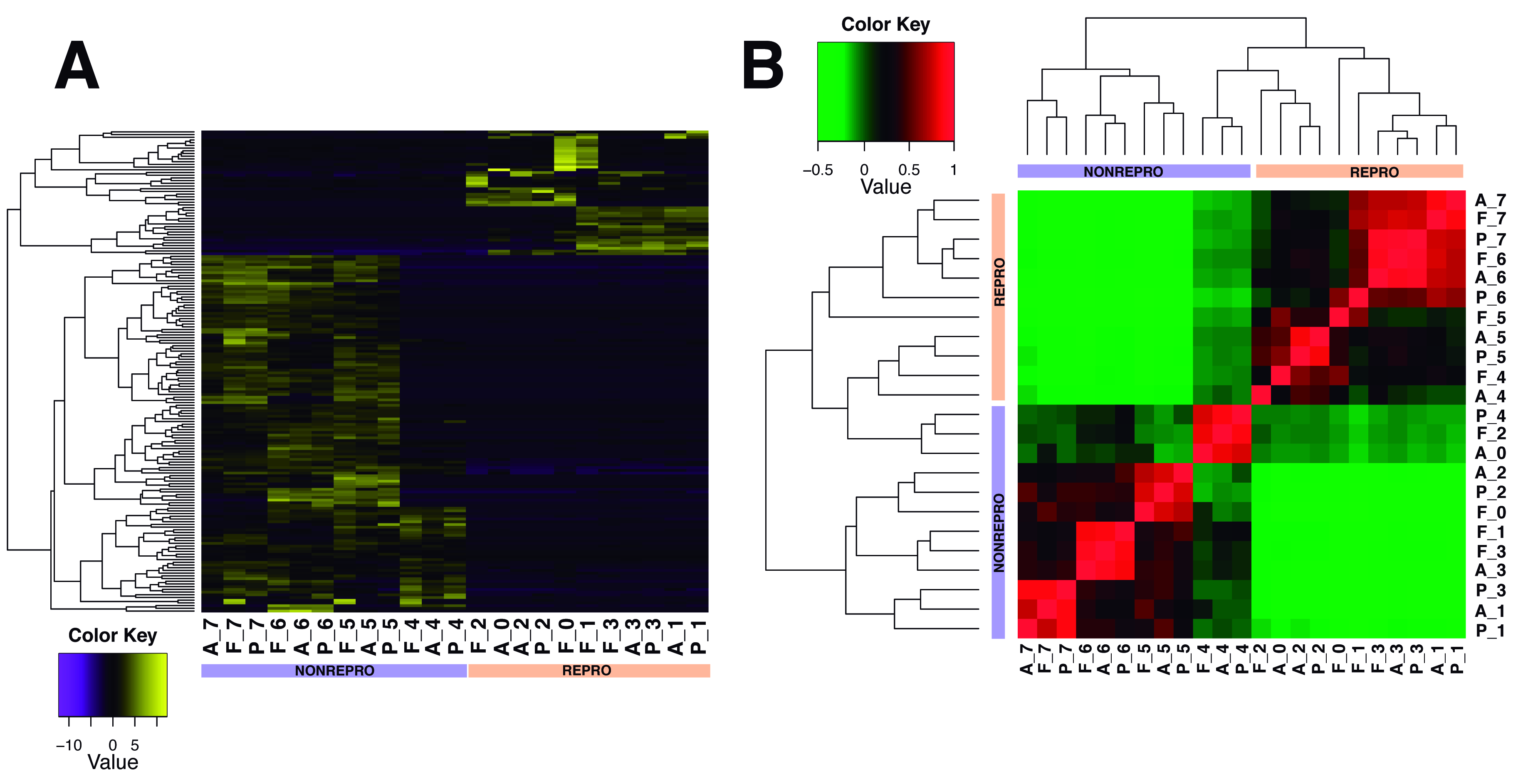

Supplement: Supplementary Data [file evy265_supp.zip › SuppFile_S4_REFSOM_difexpression.tif]

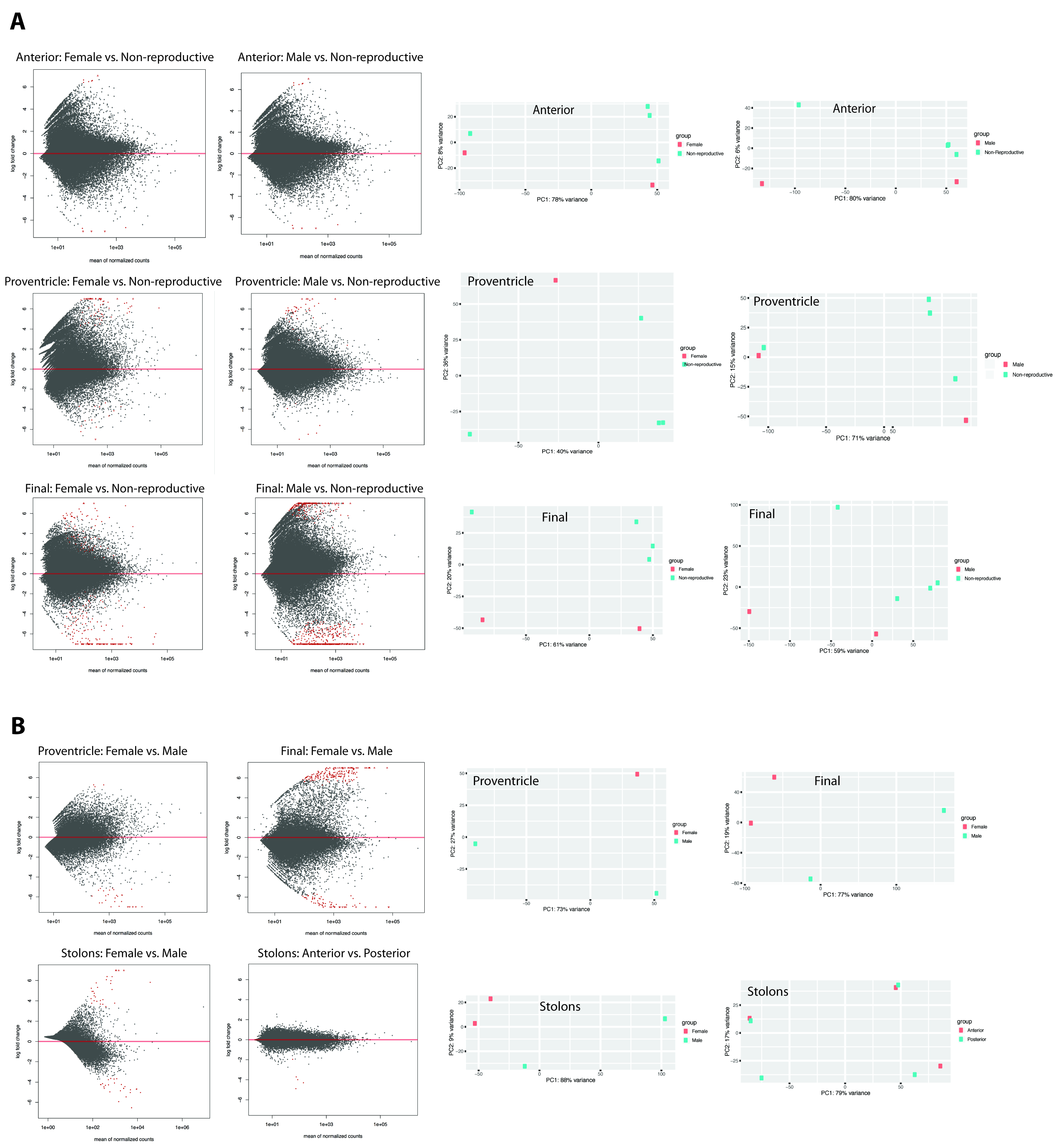

Supplement: Supplementary Data [file evy265_supp.zip › SuppFile_S5_volcanos_pcas.tif]

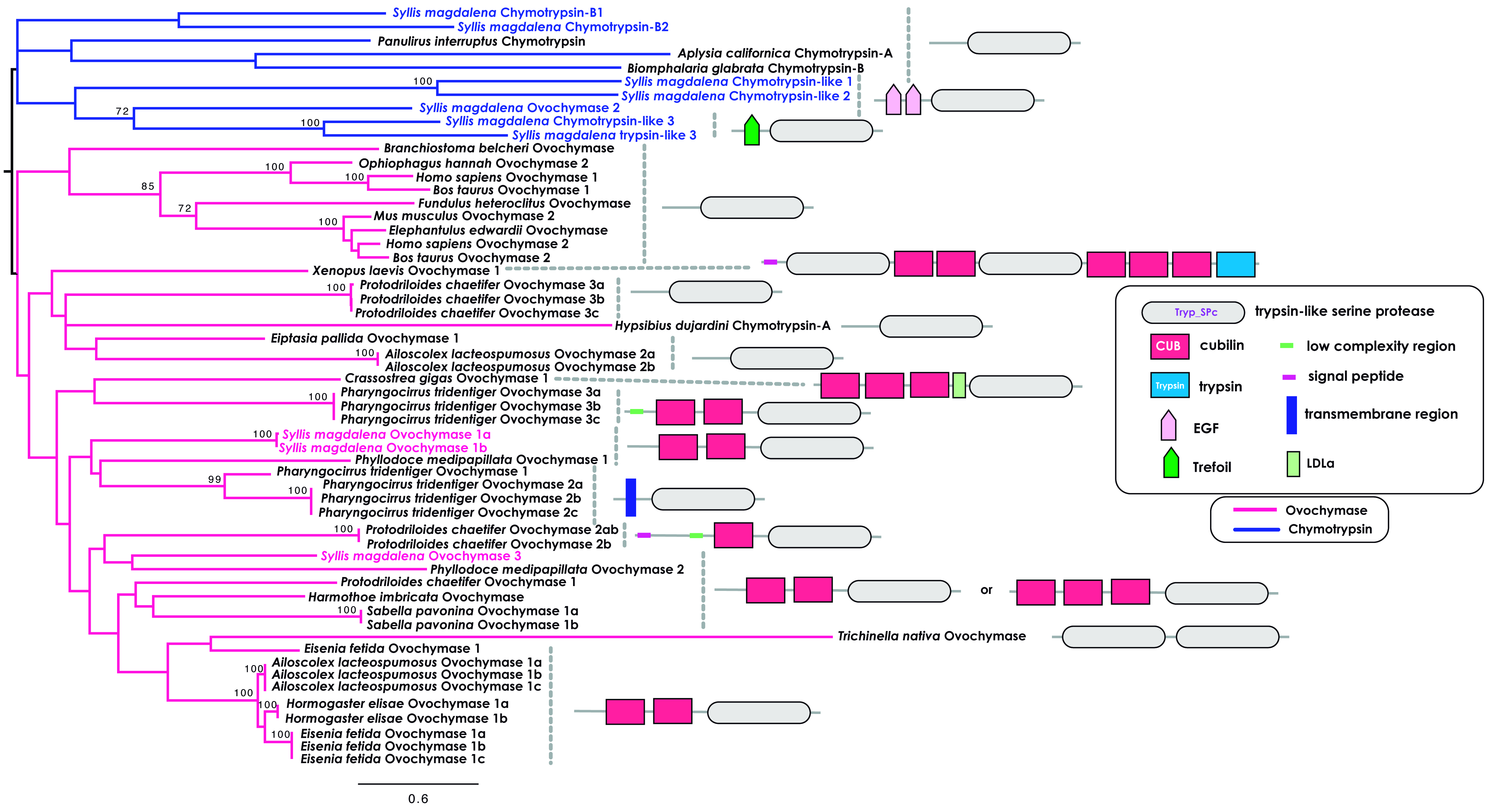

Supplement: Supplementary Data [file evy265_supp.zip › SuppFile_S8_Tree_OVOCH-CHYMOT.tif]

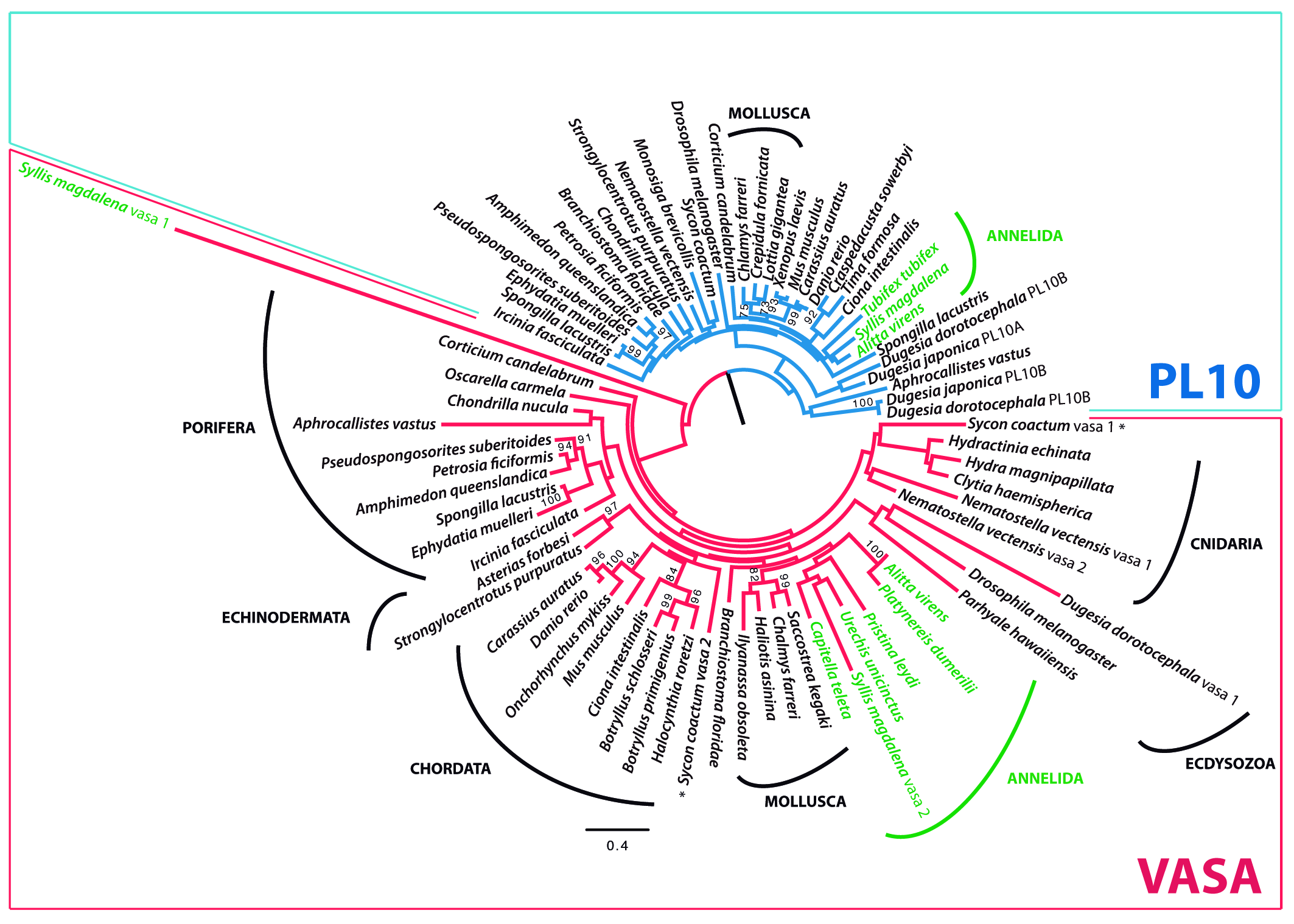

Supplement: Supplementary Data [file evy265_supp.zip › SuppFile_S9_Figurevasa_PL10.tif]
